# Lymantria Dispar Iflavirus 1 RNA Comprises a Large Proportion of RNA in Adult *L. dispar* Moths

**DOI:** 10.3390/insects14050466

**Published:** 2023-05-15

**Authors:** Michael E. Sparks, Yi-Ming Wang, Juan Shi, Robert L. Harrison

**Affiliations:** 1 Invasive Insect Biocontrol and Behavior Laboratory, USDA-ARS, Beltsville, MD 20705, USA; michael.sparks2@usda.gov; 2 Sino-France Joint Laboratory for Invasive Forest Pests in Eurasia, Beijing Forestry University, Beijing 100083, China; iris_wym@163.com

**Keywords:** iflavirus, *Lymantria dispar*, *Lymantria dispar dispar*, *Lymantria dispar asiatica*, spongy moth, flighted spongy moth, insect virus, Iflavirus lineacelli

## Abstract

**Simple Summary:**

Spongy moth, formerly known as European gypsy moth, is an invasive forest pest in the United States and is under quarantine in the eastern part of the country. The flighted spongy moth complex, a group of related spongy moths formerly known as Asian gypsy moth, are pests that are not currently present in the United States. As part of our research to characterize spongy moth pathogens, we determined the genome sequences and relative amounts of genomes of an iflavirus, an RNA-based virus, from spongy moths in two locations in the USA and flighted spongy moths in two locations in China. The iflaviruses from the USA and China spongy moth populations were distinguishable from each other on the basis of their genome sequences. Iflavirus genome levels in all moth samples were very high, but these high genome levels were not associated with any obvious disease symptoms. The information in this study raises questions about the effect of RNA viruses such as iflaviruses on their hosts, and will be of interest to scientists who work with iflaviruses, spongy moth, and other forest pests.

**Abstract:**

The spongy moth virus Lymantria dispar iflavirus 1 (LdIV1), originally identified from a *Lymantria dispar* cell line, was detected in 24 RNA samples from female moths of four populations from the USA and China. Genome-length contigs were assembled for each population and compared with the reference genomes of the first reported LdIV1 genome (Ames strain) and two LdIV1 sequences available in GenBank originating from Novosibirsk, the Russian Federation. A whole-genome phylogeny was generated for these sequences, indicating that LdIV1 viruses observed in North American (flightless) and Asian (flighted) spongy moth lineages indeed partition into clades as would be expected per their host’s geographic origin and biotype. A comprehensive listing of synonymous and non-synonymous mutations, as well as indels, among the polyprotein coding sequences of these seven LdIV1 variants was compiled and a codon-level phylogram was computed using polyprotein sequences of these, and 50 additional iflaviruses placed LdIV1 in a large clade consisting mostly of iflaviruses from other species of Lepidoptera. Of special note, LdIV1 RNA was present at very high levels in all samples, with LdIV1 reads accounting for a mean average of 36.41% (ranging from 1.84% to 68.75%, with a standard deviation of 20.91) of the total sequenced volume.

## 1. Introduction

The application of high-throughput sequencing methods to RNA samples from invertebrates has revealed a previously unknown degree of RNA virus diversity, with an expansion of many lineages specific to insects and related arthropods [1,2,3]. Among these lineages, iflaviruses (genus *Iflavirus*, family *Iflaviridae*) are highly represented in insects [4]. These viruses form non-enveloped icosahedral virions containing a genome consisting of a single, positive-sense RNA strand, approximately 9800–10,200 nt in size. Most of the genome encodes a single large open reading frame (ORF) from which a polyprotein is synthesized. The N-terminal portion of the polyprotein is processed into the structural components of the virion, which is 30–40 nm in diameter. The C-terminal portion contains non-structural proteins of the virus, including an RNA-dependent RNA polymerase (RdRp) involved in genome replication as well as an RNA helicase and processing cysteine protease. Both vertical and horizontal transmission of iflavirus infection have been documented [5,6]. Iflavirus infection can lead to overt pathology and death of the host. Diseases of honey bees (*Apis mellifera*) caused by iflaviruses have received much attention; for example, infections with sacbrood virus (*Iflavirus sacbroodi*) result in the failure of larvae to pupate, followed by death [7,8], while transmission of deformed wing virus (*Iflavirus aladeformis*) by varroa mites results in pupal death or deformities in emerging adult bees and has been associated with honey bee colony collapse [5]. Infection of the Chinese oak silkmoth, *Antheraea pernyi*, with Antheraea pernyi iflavirus (*Iflavirus vomitus*) causes a lethal disease among late-instar larvae characterized by vomiting, cessation of feeding, and falling of larvae from their host plant to the ground [9]. However, not all iflavirus infections result in disease, as covert and apparently symptomless infections have also been reported for many iflaviruses [10,11].

In a previous study, a cell line of the spongy moth, *Lymantria dispar* (Linnaeus, 1758), was found to be infected with a novel iflavirus, Lymantria dispar iflavirus 1 (LdIV1; *Iflavirus lineacelli*) [12]. Iflavirus virions of approximately 30 nm diameter were visualized in cells of the *L. dispar* ovarian cell line IPLB-Ld-652Y and found to contain an iflavirus genome-length RNA of 10,144 nt with an ORF encoding a 2980-amino acid polyprotein [12]. LdIV1 sequences were also detected in other *L. dispar* cell lines and in RNA samples from eggs and from larval and adult tissues of New Jersey Standard Strain specimens of spongy moth. No cytopathic effect (CPE) or other observable pathology was identified in cell lines containing LdIV1 or in spongy moth larvae and adults, but infection of LdIV1-free *L. dispar* cell lines was accompanied by replication of LdIV1 and CPE in the form of swollen, vacuolated cells [12].

*Lymantria dispar* has been classified into three subspecies: *L. dispar dispar*, typically found in Europe and also in North America as an invasive forest pest; *L. dispar asiatica*, typically found in China, South Korea, the Russian Far East, and Siberia; and *L. dispar japonica*, typically found in Japan [13]. The latter two subspecies, formerly known collectively as the “Asian gypsy moth” biotype, are now referred to as the “flighted spongy moth complex” along with the *Lymantria* species *L. albescens*, *L. postalba*, and *L. umbrosa* by USDA APHIS to reflect the capacity of female moths of these species to fly (https://www.aphis.usda.gov/aphis/ourfocus/planthealth/plant-pest-and-disease-programs/pests-and-diseases/sa_insects/spongy-moth (accessed on 7 March 2023)). In contrast, adult females of *L. dispar dispar*, formerly known as “European gypsy moth,” represent a separate biotype characterized by a diminished or absent flight capability. We recently reported a transcriptomic survey of adult spongy moths from four different populations, including moths from Connecticut, USA (CT); New Jersey, USA (NJ); Zunyi, Guizhou, China (ZY); and Jingeshan, Hebei, China (JGS) [14]. On the basis of geographic location, the CT and NJ populations correspond to *L. dispar dispar*, while the ZY and JGS populations correspond to *L. dispar asiatica*. During transcriptome analysis, LdIV1 sequences were detected in moths from all four populations. Here, RNA-Seq data from that study were inspected for LdIV1 viral content: geographical isolate-specific consensus genome sequences were computed and compared among themselves and with other representative LdIV1 genomes for purposes of cataloging genetic diversity within this virus species, and for performing a comprehensive phylogenetic analysis incorporating 50 additional genomes of RNA viruses isolated from arthropod hosts. The relative quantities of LdIV1 in the RNA samples were also assessed.

## 2. Materials and Methods

### 2.1. RNA-Seq Data, LdIV1 Contig Assembly, and Genome Sequence Phylogeny

RNA-Seq data recently generated from four geographical isolates of spongy moth by the authors [14] were retrieved from NCBI SRA (see Table 1). Three reference LdIV1 genome sequences were also retrieved: the original IPLB-Ld-652Y cell-line-derived sequence (“Ames strain”, NCBI reference sequence NC_024497.1, GenBank identifier: KJ629170.1) [12] and two additional LdIV1 genomes originating from Russia (MN938851.1, “Siberia” [15] and MT753155.1, “Koltsovo”). For each of the JGS, ZY, CT, and NJ datasets, reads were independently aligned in paired-end mode to the Koltsovo genome (which yielded more contiguous assemblies, results not shown) using Bowtie2 v2.4.5 [16]. Reads correctly mapping as pairs were identified by SAMtools v1.14 [17], extracted using BEDTools v2.28.0 [18], and independently assembled using SPAdes v3.15.5 [19] with MT753155.1 provided as a trusted contig. Resultant consensus genome sequences for CT, NJ, JGS, and ZY were deposited in GenBank under accession identifiers OP895018, OP895019, OP895020, and OP895021, respectively. These isolate-specific genomes were combined with the three reference whole-genome LdIV1 sequences mentioned above and multiply aligned at the nucleotide level using MUSCLE [20]. Multiple sequence alignment results were used as input to the 4 × 4 nucleotide model of DNA evolution implemented in MRBAYES [21], which was run for one million generations with 25% burn-in. The resulting tree was rendered using MEGA 11 [22].

### 2.2. Polyprotein Sequence Analysis, Codon Alignment Phylogeny, and IRES Prediction

Polyprotein annotation information for the Ames strain of LdIV1 [12] was used to infer polyprotein coding sequences (CDS) and peptide sequences for the JGS, ZY, CT, NJ, Siberia, and Koltsovo LdIV1 strains by means of tBLASTn alignments [23] and manual inspection. Polyprotein CDS and peptide sequences for JGS and ZY were identical and so were collapsed into a single entity for further analyses. The six non-redundant polyprotein amino acid sequences were multiply aligned at the protein level using MUSCLE, and the resultant multiple sequence alignment was used by EMBOSS’ tranalign utility [24] to guide a codon-level alignment of underlying CDSs. Custom scripts, available at https://github.com/scentiant/tranaln2muts (accessed on 20 March 2023), were written to exhaustively enumerate synonymous and non-synonymous mutations, and insertion–deletion (indel) mutations among these LdIV1 variants.

A set of 50 additional iflavirus genomes was obtained from the International Committee on Taxonomy of Viruses (ICTV) [25,26] and from selected sequences in the *Iflavirus* section of the NCBI Taxonomy Browser [27]. Polyprotein coding sequences (as well as translation products) were extracted on the basis of furnished annotations. These were pooled with the LdIV1 polyprotein data and globally aligned at the protein level using MUSCLE, the results of which guided a codon-level alignment of associated CDSs with tranalign. A phylogenetic tree was computed from this CDS alignment in MRBAYES using the codon model of DNA evolution presented by Goldman and Yang [28] with one million generations and a 25% burn-in, and a phylogram was rendered in MEGA 11. To detect putative internal ribosome entry sites (IRES), the 5′-untranslated regions (UTRs) of sampled iflavirus genomes were analyzed using IRESPred [29] and IRESpy [30]. Three iflavirus sequences (MH188008.1, AJ489744.2, and KF751885.1) had ambiguous nucleotide positions in their 5′-UTRs and were not able to be analyzed by IRESPred.

### 2.3. Assessing LdIV1 Transcript Levels

To provide an estimate of overall host cellular transcription dedicated to LdIV1 replication, for each of the JGS, ZY, CT, and NJ geographic isolates, their six respective RNA-Seq samples (three apiece for pre- and post-mating; see Table 1) were independently aligned to their consensus genome sequence using Bowtie2. Counts of reads properly mapping as pairs were determined using SAMtools. Pairwise comparison of percentages of reads mapping to LdIV1 in different geographic populations was carried out by unpaired *t*-test with an assumption of unequal variances and α = 0.05. This analysis was also carried out to determine if transcript levels differed between pre- and post-mating RNA samples of each geographic isolate.

## 3. Results

### 3.1. Genome Sequences of, and Relationships among, LdIV1 Variants from Different Host Spongy Moth Populations

**Figure 1:**
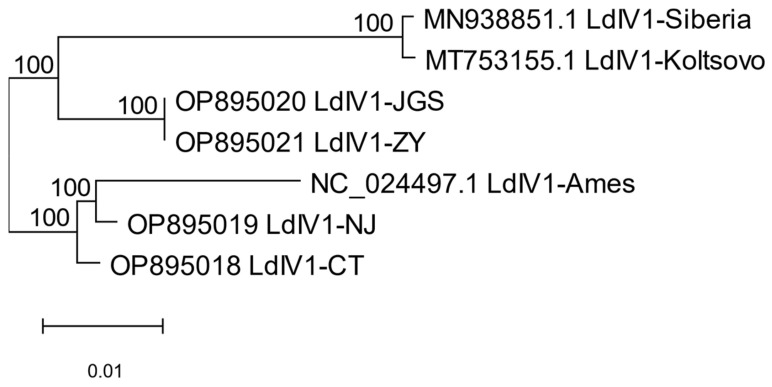
Phylogeny of LdIV1 isolates based on whole-genome alignments. GenBank accession numbers are shown for each taxon, and support for each branch is indicated.

The whole-genome phylogeny of the seven LdIV1 variants considered in this study indicates that LdIV1 viruses observed in different spongy moth lineages partitioned into clades corresponding to their respective host’s geographic origin (Figure 1). Although distinct from the other LdIV1 taxa analyzed, there were relatively few genetic differences observable between the JGS and ZY lineages at the whole-genome level; indeed, and as described above, their underlying coding sequences for the virus’ polyprotein were identical, so what few differences were present occurred in non-coding regions. The Koltsovo and Siberia strains were also highly similar at the whole-genome level, although these do exhibit differences in their respective polyprotein sequences.

### 3.2. Polyprotein Sequence Variation and Phylogeny

The peptide alignment-informed multiple codon sequence alignment for the six distinct LdIV1 polyprotein coding sequences is provided as Supplementary Information Figure S1. A total of 470 synonymous mutations, 127 non-synonymous mutations and four indels were observed among these CDSs (see Table S1, Table S2, and Table S3, respectively). A disproportionately large number of the non-synonymous mutations (78) occurred only in the sequence of the original Ames isolate. Ten of the variant amino acid positions arising from non-synonymous mutations occurred in conserved domains of the polypeptide sequence, including two mutations in the cricket paralysis virus (CRPV) capsid-like domain (accession cl07393), one in the RNA helicase domain (accession pfam00910), and seven in the RdRp catalytic core domain (accession cd23169). In addition, a two-residue deletion at polyprotein amino acid positions 1941-2 was observed in *L. dispar asiatica* iflavirus isolates JGS and ZY, and a four-residue deletion was present in the original Ames isolate (positions 1941-4) (Table S3).

**Figure 2:**
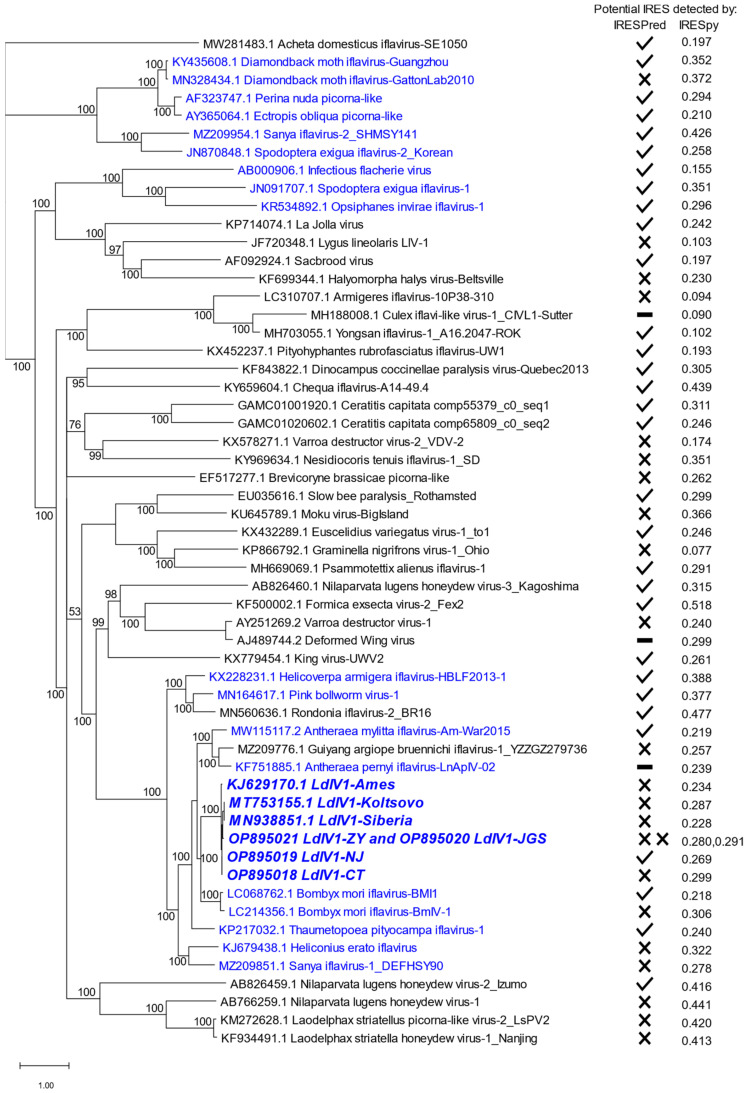
Phylogeny of LdIV1 isolates based on polyprotein sequences. LdIV1 isolates are in bold italics, and other iflaviruses originating from lepidopteran hosts are in blue type. GenBank accession numbers are shown for each taxon, and support for each branch is indicated. The prediction of a likely IRES in each taxon by IRESPred is indicated with a check mark, and the failure to detect a likely IRES is indicated with an X next to each taxon A dash (–) indicates inability to analyze the 5′ UTR sequence due to ambiguous nucleotides. The probability of a likely IRES as assessed by IRESpy is reported for each taxon next to the IRESPred results.

Within the codon-level polyprotein phylogeny for all 56 distinct iflaviral polyproteins (Figure 2), the LdIV1 variants were placed in a clade among eleven other iflaviruses isolated mostly from Lepidoptera, along with one iflavirus from a tick (Rondonia iflavirus-2) and one from a spider (Guiyang argiope bruennichi iflavirus-1). Nine additional iflaviruses from lepidopteran hosts occurred elsewhere in the phylogeny, with six of these viruses grouped in a terminal clade. In the whole-genome level phylogeny, the NJ and Ames variants are sister taxa (Figure 1), although in the polyprotein-based molecular phylogeny, NJ and CT are sister nodes (Figure 2 and Figure S2); however, at 51%, the posterior probability for the NJ-CT subtree is the lowest level of support observed in the overall tree, owing to limited divergence levels between these two LdIV1 variants (Figure S2).

Translation of the iflavirus polyprotein ORF appears to be mediated by initiation at an internal ribosome entry site (IRES) in the 5′-UTR of the genomic RNA [31,32]. Analysis of the 5′ UTR sequences of LdIV1 isolates with IRESPred predicted a potential IRES for LdIV1-NJ but not for the other LdIV1 isolates. Potential IRES were also predicted for 30 of 47 other 5′-UTRs of the other iflaviruses in Figure 2. The probability of a potential IRES in the 5′ UTRs of the LdIV isolates as determined by IRESpy ranged from 0.228 (LdIV1-Siberia) to 0.299 (LdIV1-CT). In comparison, the probabilities of predicted IRES assessed by IRESpy among all the iflavirus sequences in Figure 2 ranged from 0.077 (Graminella nigrifrons virus-1) to 0.518 (Formica exsecta virus-2).

### 3.3. Steady-State LdIV1 Transcript Levels in Adult Spongy Moths

**Figure 3:**
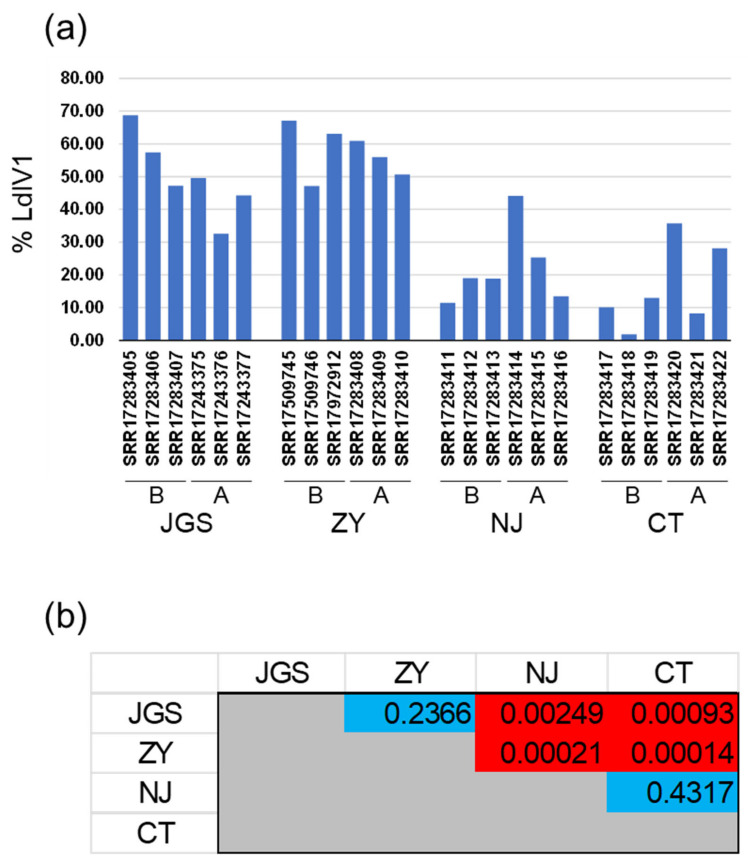
Relative levels of LdIV1 RNA in adult spongy moths from different populations. (**a**) Percentages of total RNA-Seq reads mapping to LdIV1 in different geographic populations. The percentage for each of the three RNA samples harvested before mating (B) and after mating and oviposition (A) is shown for each population. (**b**) *p*-values for unpaired *t*-tests between LdIV1 RNA percentages in (a), assuming unequal variances. Red-shaded cells indicate *p*-values < 0.05 for pairwise comparisons between samples from USA (NJ, CT) and China (JGS, ZY) populations.

LdIV1 RNA levels as a percentage of total mRNA (per RNA-Seq sequencing volume) ranged from a minimum of 1.84% (CT’s pre-mating SRR17283418 sample) to a maximum of 68.75% (JGS’s pre-mating SRR17283405 sample) and had a mean average of 36.41% (sample standard deviation = 20.91) over all 24 measurements (Figure 3, Table S4). Pairwise comparisons revealed that the LdIV1 RNA levels in the CT and NJ samples as a proportion of total RNA were significantly lower (*p* < 0.05) when compared with the ZY and JGS samples, but significant differences were not detected in comparisons between the two USA samples or between the two China samples (Figure 3, Table S4). No statistically significant differences in viral load associated with mating status were observed.

## 4. Discussion

As the seven LdIV1 variants addressed by this report are members of a single *Iflavirus* species, it would be expected that low levels of divergence exist among them, posing a challenge for phylogenetic inference methods. Indeed, low divergence levels were observed in this group, two members of which (specifically, JGS and ZY) exhibited 100% sequence identity between their respective polyprotein coding sequences. However, the whole-genome phylogeny grouped LdIV1 variants isolated from flighted spongy moths (JGS, ZY, Siberia and Koltsovo) and those isolated from flightless North American spongy moths (CT, NJ and Ames) into separate clades with 100% support for all internal nodes, suggesting these isolates have been proceeding along independent evolutionary courses corresponding to their insect host population’s respective biotype and geographical placement. Similar results have shown that isolates of the spongy moth alphabaculovirus, *Lymantria dispar* multiple nucleopolyhedrovirus (LdMNPV), also segregated into host subspecies/biotype- and geography-specific clades of core gene phylogenies [33]. The finding of the same spongy moth viruses in different geographic and taxonomic groupings of *L. dispar* and the evident co-evolution of virus populations with their host populations suggest that, for purposes of identifying the geographical origin of spongy moths intercepted during commerce-related transactions, use of diagnostic markers for identifying the strain of LdIV1, LdMNPV, or other viruses present in moths could be used either in conjunction with or as an alternative to genetic peculiarities of the host insect proper [34], although an exchange of viruses among mixing populations of *L. dispar* would tend to blunt the usefulness of such an approach over time.

The original Ames isolate of LdIV1 was obtained from the IPLB-Ld-652Y cell line, rather than from field-caught or laboratory-reared specimens. The IPLB-Ld-652Y cell line was prepared from immature ovarian tissue of spongy moth in Beltsville, MD, presumably from specimens originating from the spongy moth quarantine area in the northeastern USA [35]. However, LdIV1-Ames was separated from the other northeastern USA isolates by a relatively long branch in the whole-genome phylogeny (Figure 1), and was grouped separately from the other isolates in the polyprotein ORF phylogeny (Figure S2), indicating a greater degree of divergence than expected. This result is consistent with the 37 synonymous substitutions and the 78 non-synonymous substitutions that are unique to the LdIV1-Ames sequence (Tables S1–S3). Similarly, the LdMNPV-5/6 alphabaculovirus isolate, a plaque-purified clone produced by passage through the IPLB-Ld-652Y cell line [36,37], was also found to be separated from other *L. dispar dispar*-derived LdMNPV isolates by relatively long branches in phylogenies [33,38,39]. These relatively long branch lengths suggest a greater degree of divergence in viruses replicating in the IPLB-Ld-652Y cell line that is consistent with a host cell environment (e.g., a cultured cell line) that was markedly different than that encountered in intact *L. dispar* insects and thus may have exerted qualitatively different selection pressures on replicating viruses.

The results of IRES prediction software IRESPred and IRESpy to identify potential IRES in LdIV1 and other iflaviruses (Figure 2) highlighted the limitations of such software for unambiguous identification of potential IRES in iflaviruses. In three separate instances involving LdIV1-NJ, Bombyx mori iflavirus-BMI1, and diamondback moth iflavirus-Guangzhou, IRESPred identified a potential IRES in one isolate of a group of iflaviruses of the same species but failed to also identify an IRES in other isolates of the same group (Figure 2). IRESpy provided a probability of an IRES in a sequence, and while the distribution of probabilities among the iflaviruses in Figure 2 resembles the distribution of probabilities found among datasets of IRES-containing sequences employed by the developers of IRESpy to train and validate the program [30], only one iflavirus sequence (Formica exsecta virus-2_Fex2) was determined to have a potential IRES with a probability > 0.5. Viral IRES are difficult to identify because they are poorly conserved, both in sequence and length, and lack any structural features that are common among different groups of RNA viruses [40]. Thus, confirmation of potential IRES in any given virus requires experimentation.

The remarkably high levels of LdIV1 transcription observed in the spongy moth non-ribosomal RNA samples characterized here (Figure 3) make it evident that these iflaviruses are quite adept at exploiting cellular resources of their spongy moth hosts in pursuit of their own biological interests. LdIV1 viral load was high (>1% of non-ribosomal RNA) in every sample but varied among samples of the same population, particularly among samples of the North American isolates. Of note was the observation that LdIV1 expression levels found in the spongy moth populations from China were significantly higher than those in the USA populations. The results seen with the USA and China samples contrasted with the relative level of LdIV1 RNA observed by Pavlushin et al. [15] in a western Siberian population, suggesting the possibility that some populations of *L. dispar* do not contain high levels of LdIV1 RNA. However, the results presented in that study and those conveyed here are not directly comparable, owing to differing methodologies; as Pavlushin et al. did not publicly deposit the RNA-Seq data used to assemble the MN938851 LdIV1 genome consensus sequence, applying the methods of this study to those data was not possible.

High levels of RNA virus expression in arthropods have been observed more generally [1,2,41] but not understood in any depth. For example, in the Shi et al. study [2], 63 of 87 different RNA-Seq libraries from pools of insects and other invertebrates contained at least one virus whose RNAs made up >0.1% of non-ribosomal RNA reads. Thirteen distinct viral sequences accounting for 0.1–1% of reads and nine viral sequences constituting >1% of reads from this study were placed in an iflavirus-containing clade of an RdRp tree, indicating that the accumulation of very high levels of iflavirus RNA in insect hosts is not unique to LdIV1 [2]. It is unknown what impact such high levels of viral RNA are having on their hosts. In addition, a series of studies with iflaviruses and an alphabaculovirus of the beet armyworm, *Spodoptera exigua*, have described interactions between iflaviruses and baculoviruses that result in the modulation of pathogenicity of one virus by the other [42,43,44,45]. Further experiments are needed to understand the effect of high levels of LdIV1 RNA on spongy moth and the interactions between LdIV1 and LdMNPV. Furthermore, an exploration of the nature of LdIV1 transcriptional regulation could be of significant relevance to the biological control of *L. dispar*, a notorious worldwide pest of trees and forests.

## 5. Conclusions

The data in this paper indicate that the LdIV1 iflavirus is present in a wide range of populations of the host spongy moth. Different isolates of this virus group together in population-specific clades, suggesting that the virus co-evolves with its host population. The representative isolate of this virus, LdIV1-Ames, was originally identified from a spongy moth cell line and has diverged from LdIV1 isolates originally identified in moths to an extent that is consistent with replication in different environments (e.g., a cultured cell line, as opposed to a moth).

RNA-Seq reads mapping to the LdIV1 sequence make up a strikingly large percentage of reads in adult moths, and moth populations from China possessed a significantly larger viral load (in terms of the proportion of reads mapping to the LdIV1 sequence) than populations from the USA. This large viral load is not correlated with any obvious pathology. This phenomenon has been observed with RNA viruses identified in the transcriptomes of other invertebrates, but our paper is the first demonstration that very high loads of an RNA virus can be repeatedly and consistently observed with different populations of the same virus and host.

## Figures and Tables

**Table 1:** NCBI SRA accessions of *Lymantria dispar* RNA-Seq samples as previously reported in Wang et al. (2022) [14].

Spongy Moth Population	Time of RNA Harvest	SRA Identifiers
Jingeshan, Hebei, China (JGS)	Before mating	SRR17283405, SRR17283406, SRR17283407
	After mating and oviposition	SRR17243375, SRR17243376, SRR17243377
Zunyi, Guizhou, China (ZY)	Before mating	SRR17509745, SRR17509746, SRR17972912
	After mating and oviposition	SRR17283408, SRR17283409, SRR17283410
New Jersey, USA (NJ)	Before mating	SRR17283411, SRR17283412, SRR17283413
	After mating and oviposition	SRR17283414, SRR17283415, SRR17283416
Connecticut, USA (CT)	Before mating	SRR17283417, SRR17283418, SRR17283419
	After mating and oviposition	SRR17283420, SRR17283421, SRR17283422

## Data Availability

RNA-Seq data analyzed in this study are available at the NCBI Sequence Read Archive (https://www.ncbi.nlm.nih.gov/sra (accessed on 8 November 2022)) under BioProject accession numbers PRJNA789495 and PRJNA788963. Assembled sequences reported in this study have been deposited in NCBI GenBank (https://www.ncbi.nlm.nih.gov/genbank/ (accessed on 20 March 2023)) under accession numbers OP895018, OP895019, OP895020, and OP895021.

## Supplementary Materials

The following supporting information can be downloaded at: https://www.mdpi.com/article/10.3390/insects14050466/s1, Figure S1: LdIV1 polyprotein sequence alignment; Figure S2: Subtree of Figure 2 polyprotein ORF tree, showing relationships of LdIV1 isolates; Table S1: Polyprotein synonymous substitutions; Table S2: Polyprotein non-synonymous substitutions; Table S3: Polyprotein indels; Table S4: LdIV1 RNA read counts and percentages of total RNA.
