# Supplementary material for: Lymantria Dispar Iflavirus 1 RNA Comprises a Large Proportion of RNA in Adult L. dispar Moths
Source: Insects. 2023 May 15;14(5):466. doi: 10.3390/insects14050466 (PMC10231084; doi:10.3390/insects14050466)
Supplement: Supplementary file 1 [file insects-14-00466-s001.zip › Figure_S1.html]

LdIV1 polyprotein sequence alignment


- Alignment site positions (amino acids or, equivalently, codons) displayed at a given stanza are shown in square brackets.
- Taxa identifiers are placed on lines displaying codons; those codons' associated amino acids are shown on the line immediately above.
- Sites exhibiting non-synonymous and/or indel mutations are flagged with a '^' symbol; synonymous mutations are indicated using '='.

```
[1..20]
                M   A   S   F   N   V   S   F   L   Q   S   V   F   S   D   L   I   S   A   E  
MT753155.1     ATG GCT TCT TTT AAC GTA TCT TTT CTC CAA TCT GTT TTT AGC GAT TTG ATA TCG GCT GAA 
                M   A   S   F   N   V   S   F   L   Q   S   V   F   S   D   L   I   S   A   E  
MN938851.1     ATG GCT TCT TTT AAC GTA TCT TTT CTC CAA TCT GTT TTT AGC GAT TTG ATA TCG GCT GAA 
                M   A   S   F   N   V   S   F   L   Q   S   V   F   S   D   L   I   S   A   E  
LdIV1_JGS      ATG GCT TCT TTT AAC GTA TCT TTT CTC CAA TCT GTT TTT AGT GAT TTA ATT TCA GCT GAG 
                M   A   S   F   N   V   S   F   L   Q   S   V   F   S   D   L   I   S   A   E  
LdIV1_ZY       ATG GCT TCT TTT AAC GTA TCT TTT CTC CAA TCT GTT TTT AGT GAT TTA ATT TCA GCT GAG 
                M   A   S   F   N   V   S   F   L   Q   S   V   F   S   D   L   I   S   A   E  
LdIV1_NJ       ATG GCT TCT TTT AAC GTA TCT TTT CTC CAA TCT GTT TTT AGT GAT TTA ATT TCA GCT GAG 
                M   A   S   F   N   V   S   F   L   Q   S   V   F   S   D   L   I   S   A   E  
LdIV1_CT       ATG GCT TCT TTC AAT GTA TCT TTT CTC CAA TCT GTT TTT AGT GAT TTA ATT TCA GCT GAG 
                M   A   S   F   N   V   S   F   L   Q   S   V   F   S   D   L   I   S   A   E  
KJ629170.1     ATG GCT TCT TTY AAC GTA TCT TTT CTC CAA TCT GTT TTT AGT GAT TTA ATT TCA GCT GAG 
                            =   =                                   =       =   =   =       =  


[21..40]
                F   D   Y   E   V   T   L   L   R   K   I   F   D   G   K   Y   E   L   R   H  
MT753155.1     TTT GAC TAT GAA GTT ACC TTA CTT CGT AAA ATT TTT GAT GGA AAA TAT GAA TTG CGT CAT 
                F   D   Y   E   V   T   L   L   R   K   I   F   D   G   K   Y   E   L   R   H  
MN938851.1     TTT GAC TAT GAA GTT ACC TTA CTT CGT AAA ATT TTT GAT GGA AAA TAT GAA TTG CGT CAT 
                F   D   Y   E   V   T   L   L   R   K   I   F   D   G   K   Y   E   L   R   H  
LdIV1_JGS      TTT GAT TAC GAA GTT ACC TTA CTT CGT AAA ATT TTT GAT GGC AAA TAT GAA TTG CGT CAT 
                F   D   Y   E   V   T   L   L   R   K   I   F   D   G   K   Y   E   L   R   H  
LdIV1_ZY       TTT GAT TAC GAA GTT ACC TTA CTT CGT AAA ATT TTT GAT GGC AAA TAT GAA TTG CGT CAT 
                F   D   Y   E   V   T   L   L   R   K   I   F   D   G   K   Y   E   L   R   H  
LdIV1_NJ       TTT GAT TAC GAA GTT ACC TTA CTT CGT AAA ATT TTT GAT GGA AAA TAT GAA TTG CGT CAT 
                F   D   Y   E   V   T   L   L   R   K   I   F   D   G   K   Y   E   L   R   H  
LdIV1_CT       TTT GAT TAC GAA GTT ACC TTA CTT CGT AAA ATT TTT GAT GGA AAA TAT GAA TTG CGT CAT 
                F   D   Y   E   V   T   L   L   R   K   I   F   D   G   K   Y   E   L   R   H  
KJ629170.1     TTT GAT TAC GAA GTT ACC TTA CTT CGT AAA ATT TTT GAT GGA AAA TAT GAA TTG CGT CAT 
                    =   =                                           =                          


[41..60]
                S   Y   G   L   T   S   S   V   D   D   I   H   L   E   D   E   W   R   F   R  
MT753155.1     TCT TAT GGA CTG ACA TCA TCT GTG GAT GAT ATT CAT CTT GAG GAT GAA TGG CGC TTT AGA 
                S   Y   G   L   T   S   S   V   D   D   I   H   L   E   D   E   W   R   F   R  
MN938851.1     TCT TAT GGA CTG ACA TCA TCT GTG GAT GAT ATT CAT CTT GAG GAT GAA TGG CGC TTT AGA 
                S   Y   G   L   T   S   S   V   D   D   I   H   L   E   D   E   W   R   F   R  
LdIV1_JGS      TCT TAT GGA CTA ACA TCA TCT GTA GAC GAT ATT CAT CTT GAA GAT GAA TGG CGC TTT AGA 
                S   Y   G   L   T   S   S   V   D   D   I   H   L   E   D   E   W   R   F   R  
LdIV1_ZY       TCT TAT GGA CTA ACA TCA TCT GTA GAC GAT ATT CAT CTT GAA GAT GAA TGG CGC TTT AGA 
                S   Y   G   L   T   S   S   V   D   D   I   H   L   E   D   E   W   R   F   R  
LdIV1_NJ       TCT TAT GGA CTA ACA TCA TCT GTA GAT GAT ATT CAT CTT GAA GAT GAA TGG CGC TTT AGA 
                S   Y   G   L   T   S   S   V   D   D   I   H   L   E   D   E   W   R   F   R  
LdIV1_CT       TCT TAT GGA CTG ACA TCA TCT GTA GAC GAT ATT CAT CTT GAA GAT GAA TGG CGC TTT AGA 
                S   Y   G   L   T   S   S   L   D   D   N   H   L   E   D   E   W   R   F   R  
KJ629170.1     TCT TAT GGA CTG ACA TCA TCM TTA GAC GAT AAT CAT CTT GAA GAT GAA TGG CGC TTT AGA 
                            =           =   ^   =       ^           =                          


[61..80]
                Y   I   K   L   E   K   R   R   N   Y   L   W   Y   L   M   K   T   R   K   F  
MT753155.1     TAT ATT AAG TTA GAA AAA CGC CGT AAT TAT CTT TGG TAT TTA ATG AAA ACG CGA AAA TTT 
                Y   I   K   L   E   K   R   R   N   Y   L   W   Y   L   M   K   T   R   K   F  
MN938851.1     TAT ATT AAG TTA GAA AAA CGC CGT AAT TAT CTT TGG TAT TTA ATG AAA ACG CGA AAA TTT 
                Y   L   K   L   E   K   R   R   N   Y   L   W   Y   L   M   K   T   Q   K   F  
LdIV1_JGS      TAT CTT AAG TTA GAA AAA CGT CGT AAT TAT CTT TGG TAT TTA ATG AAA ACG CAA AAA TTT 
                Y   L   K   L   E   K   R   R   N   Y   L   W   Y   L   M   K   T   Q   K   F  
LdIV1_ZY       TAT CTT AAG TTA GAA AAA CGT CGT AAT TAT CTT TGG TAT TTA ATG AAA ACG CAA AAA TTT 
                Y   L   K   L   E   K   R   R   N   Y   L   W   Y   L   M   K   T   Q   K   F  
LdIV1_NJ       TAT CTT AAG TTA GAA AAA CGT CGT AAT TAT CTT TGG TAT TTA ATG AAA ACG CAA AAA TTT 
                Y   L   K   L   E   K   R   R   N   Y   L   W   Y   L   M   K   T   Q   K   F  
LdIV1_CT       TAT CTT AAG TTA GAA AAA CGT CGT AAT TAT CTT TGG TAT TTA ATG AAA ACG CAA AAA TTT 
                X   F   K   L   E   K   R   R   N   Y   L   W   Y   L   M   K   T   Q   K   F  
KJ629170.1     TWT TTT AAG TTA GAA AAA CGT CGC AAT TAT CTT TGG TAT TTA ATG AAA ACA CAA AAA TTT 
                ^   ^                   =   =                                   =   ^          


[81..100]
                V   E   L   D   D   H   F   N   T   D   Y   T   F   S   D   L   D   L   R   L  
MT753155.1     GTA GAA TTA GAT GAT CAT TTT AAT ACA GAT TAT ACC TTT AGT GAT TTA GAT TTA AGA TTG 
                V   E   L   D   D   H   F   N   T   D   Y   T   F   S   D   L   D   L   R   L  
MN938851.1     GTA GAA TTA GAT GAT CAT TTT AAT ACA GAT TAT ACC TTT AGT GAT TTA GAT TTA AGA TTG 
                V   E   L   D   D   H   F   N   T   D   Y   T   F   S   D   L   D   L   R   L  
LdIV1_JGS      GTA GAA TTA GAT GAT CAT TTT AAT ACG GAT TAT ACT TTT AGC GAT TTA GAT TTG AGA TTA 
                V   E   L   D   D   H   F   N   T   D   Y   T   F   S   D   L   D   L   R   L  
LdIV1_ZY       GTA GAA TTA GAT GAT CAT TTT AAT ACG GAT TAT ACT TTT AGC GAT TTA GAT TTG AGA TTA 
                V   E   L   D   D   H   F   N   T   D   Y   T   F   S   D   L   D   L   R   L  
LdIV1_NJ       GTA GAA TTA GAT GAT CAT TTT AAT ACG GAT TAT ACT TTT AGC GAT TTA GAT TTG AGA CTG 
                V   E   L   D   D   H   F   N   T   D   Y   T   F   S   D   L   D   L   R   L  
LdIV1_CT       GTA GAA TTA GAT GAT CAT TTT AAT ACG GAT TAT ACT TTT AGC GAT TTA GAT TTG AGA CTG 
                V   E   L   D   D   H   F   N   T   D   Y   T   F   G   D   L   D   L   R   L  
KJ629170.1     GTA GAA TTA GAT GAT CAT TTT AAT ACG GAT TAT ACC TTT GGC GAT TTA GAT TTG AGA TTG 
                                                =           =       ^               =       =  


[101..120]
                R   V   E   F   S   R   H   V   K   E   H   N   D   R   F   K   Y   I   N   R  
MT753155.1     CGA GTA GAA TTT AGT AGA CAT GTT AAA GAA CAT AAT GAT AGA TTT AAA TAT ATT AAC CGT 
                R   V   E   F   S   R   H   V   K   E   H   N   D   R   F   K   Y   I   N   R  
MN938851.1     CGA GTA GAA TTT AGT AGA CAT GTT AAA GAA CAT AAT GAT AGA TTT AAA TAT ATT AAC CGT 
                R   V   E   F   S   R   H   V   K   E   H   N   D   R   F   K   Y   I   N   R  
LdIV1_JGS      CGA GTA GAA TTT AGT AGA CAT GTT AAA GAA CAT AAT GAT AGA TTT AAA TAT ATT AAC CGT 
                R   V   E   F   S   R   H   V   K   E   H   N   D   R   F   K   Y   I   N   R  
LdIV1_ZY       CGA GTA GAA TTT AGT AGA CAT GTT AAA GAA CAT AAT GAT AGA TTT AAA TAT ATT AAC CGT 
                R   V   E   F   S   R   H   V   K   E   H   S   D   R   F   K   Y   I   N   R  
LdIV1_NJ       CGA GTA GAA TTT AGT AGA CAT GTT AAA GAA CAT AGT GAT AGA TTT AAA TAT ATT AAC CGT 
                R   V   E   F   S   R   H   V   K   E   H   S   D   R   F   K   Y   I   N   R  
LdIV1_CT       CGA GTA GAA TTT AGT AGA CAT GTT AAA GAA CAT AGT GAT AGA TTT AAA TAT ATT AAC CGT 
                R   V   E   F   S   R   X   V   K   E   H   S   D   R   Y   K   Y   I   N   R  
KJ629170.1     CGA GTA GAA TTT AGT AGA YAC GTT AAA GAA CAT AGT GAT AGA TAT AAA TAT ATT AAC CGT 
                                        ^                   ^           ^                      


[121..140]
                K   S   R   F   V   P   F   S   K   S   S   L   P   V   E   N   I   E   T   E  
MT753155.1     AAA TCG CGA TTT GTG CCG TTT AGT AAG AGT AGC CTC CCC GTT GAA AAT ATT GAA ACG GAA 
                K   S   R   F   V   P   F   S   K   S   S   L   P   V   E   N   I   E   T   E  
MN938851.1     AAA TCG CGA TTT GTG CCG TTT AGT AAG AGT AGC CTC CCC GTT GAA AAT ATT GAA ACG GAA 
                K   S   R   F   V   P   F   S   K   S   S   L   P   V   E   N   I   E   T   E  
LdIV1_JGS      AAA TCG CGA TTC GTG CCG TTT AGT AAG AGT AGC CTC CCC GTT GAA AAT ATT GAA ACG GAA 
                K   S   R   F   V   P   F   S   K   S   S   L   P   V   E   N   I   E   T   E  
LdIV1_ZY       AAA TCG CGA TTC GTG CCG TTT AGT AAG AGT AGC CTC CCC GTT GAA AAT ATT GAA ACG GAA 
                K   S   R   F   V   P   F   S   K   S   S   L   P   V   E   N   I   E   T   E  
LdIV1_NJ       AAA TCA CGA TTT GTG CCG TTT AGT AAG AGT AGT CTC CCC GTT GAG AAT ATT GAA ACG GAA 
                K   S   R   F   V   P   F   S   K   S   S   L   P   V   E   N   I   E   T   E  
LdIV1_CT       AAA TCA CGA TTT GTG CCG TTT AGT AAG AGT AGT CTC CCC GTT GAG AAT ATT GAA ACG GAA 
                K   S   R   F   V   P   F   S   K   S   S   L   P   V   E   D   I   E   T   E  
KJ629170.1     AAA TCA CGA TTT GTT CCR TTT AGT AAA AGT AGT CTC CCC GTT GAG GAT ATT GAA ACG GAA 
                    =       =   =   =           =       =               =   ^                  


[141..160]
                H   Y   D   N   N   D   I   D   I   D   F   S   T   N   S   V   S   F   K   K  
MT753155.1     CAT TAT GAT AAT AAT GAT ATA GAT ATT GAT TTT TCG ACA AAT TCT GTT AGT TTT AAA AAG 
                H   Y   D   N   N   D   I   D   I   D   F   S   T   N   S   V   S   F   K   K  
MN938851.1     CAT TAT GAT AAT AAT GAT ATA GAT ATT GAT TTT TCG ACA AAT TCT GTT AGT TTT AAA AAG 
                H   Y   D   N   N   D   I   D   I   D   F   S   T   N   S   V   S   F   R   K  
LdIV1_JGS      CAT TAT GAT AAT AAT GAT ATA GAT ATT GAT TTT TCT ACA AAT TCT GTT AGT TTT AGA AAG 
                H   Y   D   N   N   D   I   D   I   D   F   S   T   N   S   V   S   F   R   K  
LdIV1_ZY       CAT TAT GAT AAT AAT GAT ATA GAT ATT GAT TTT TCT ACA AAT TCT GTT AGT TTT AGA AAG 
                Y   Y   D   N   N   D   I   D   I   D   F   S   T   N   S   V   S   F   R   K  
LdIV1_NJ       TAT TAT GAT AAT AAT GAT ATA GAT ATT GAT TTC TCT ACA AAT TCT GTT AGT TTT AGA AAG 
                Y   Y   D   N   N   D   I   D   I   D   F   S   T   N   S   V   S   F   R   K  
LdIV1_CT       TAT TAT GAT AAT AAT GAT ATA GAT ATT GAT TTC TCT ACA AAT TCT GTT AGT TTT AGA AAG 
                Y   Y   D   N   N   D   I   D   I   D   F   S   T   N   S   V   S   F   R   K  
KJ629170.1     TAT TAT GAT AAT AAT GAT ATA GAT ATT GAT TTC TCA ACA AAT TCT GTT AGT TTT AGA AAG 
                ^                                       =   =                           ^      


[161..180]
                K   R   S   V   S   F   E   S   L   V   D   N   N   R   I   Y   K   K   C   E  
MT753155.1     AAA CGA AGT GTT TCC TTT GAA TCA TTA GTA GAT AAT AAT CGT ATA TAT AAA AAA TGT GAG 
                K   R   S   V   S   F   E   S   L   V   D   N   N   R   I   Y   K   K   C   E  
MN938851.1     AAA CGA AGT GTT TCC TTT GAA TCA TTA GTA GAT AAT AAT CGT ATA TAT AAA AAA TGT GAG 
                K   R   S   V   S   F   E   S   L   V   D   N   N   R   I   Y   R   K   C   E  
LdIV1_JGS      AAA CGA AGT GTT TCT TTT GAA TCA TTA GTT GAT AAT AAT CGT ATA TAT AGA AAA TGT GAA 
                K   R   S   V   S   F   E   S   L   V   D   N   N   R   I   Y   R   K   C   E  
LdIV1_ZY       AAA CGA AGT GTT TCT TTT GAA TCA TTA GTT GAT AAT AAT CGT ATA TAT AGA AAA TGT GAA 
                K   R   S   V   S   F   E   S   L   V   D   N   N   R   I   Y   R   K   C   E  
LdIV1_NJ       AAA CGA AGT GTT TCT TTT GAA TCA TTA GTT GAT AAT AAT CGT ATA TAT AGA AAA TGT GAA 
                K   R   S   V   S   F   E   S   L   V   D   N   N   R   I   Y   R   K   C   E  
LdIV1_CT       AAA CGA AGT GTT TCT TTT GAA TCA TTA GTT GAT AAT AAT CGT ATA TAT AGA AAA TGT GAA 
                K   R   S   V   S   F   E   S   L   V   D   N   N   R   V   Y   R   K   C   E  
KJ629170.1     AAA CGA AGT GTT TCT TTT GAA TCA TTA GTT GAT AAT AAT CGT GTA TAT AGA AAA TGT GAA 
                                =                   =                   ^       ^           =  


[181..200]
                V   D   N   R   D   Y   N   L   L   V   S   Y   A   L   R   L   R   E   L   L  
MT753155.1     GTA GAT AAT AGA GAT TAT AAT CTT TTA GTT AGT TAT GCT TTA CGT TTA AGA GAA TTA TTA 
                V   D   N   R   D   Y   N   L   L   V   S   Y   A   L   R   L   R   E   L   L  
MN938851.1     GTA GAT AAT AGA GAT TAT AAT CTT TTA GTT AGT TAT GCT TTA CGT TTA AGA GAA TTA TTA 
                V   D   N   R   D   Y   N   L   L   V   S   Y   A   L   R   L   R   E   L   L  
LdIV1_JGS      GTA GAT AAT AGA GAT TAT AAC CTT TTA GTT AGT TAT GCT TTA CGT TTA AGA GAA TTA TTA 
                V   D   N   R   D   Y   N   L   L   V   S   Y   A   L   R   L   R   E   L   L  
LdIV1_ZY       GTA GAT AAT AGA GAT TAT AAC CTT TTA GTT AGT TAT GCT TTA CGT TTA AGA GAA TTA TTA 
                V   D   N   R   D   Y   N   L   L   V   S   Y   A   L   R   L   R   E   L   L  
LdIV1_NJ       GTA GAT AAT AGA GAT TAT AAC CTT TTA GTT AGT TAT GCT TTA CGT TTA AGA GAA TTA TTA 
                V   D   N   R   D   Y   N   L   L   V   S   Y   A   L   R   L   R   E   L   L  
LdIV1_CT       GTA GAT AAT AGA GAT TAT AAC CTT TTA GTT AGT TAT GCT TTA CGT TTA AGA GAA TTA TTA 
                V   D   N   R   D   Y   N   L   L   V   S   Y   A   L   R   L   R   E   L   I  
KJ629170.1     GTA GAT AAT AGA GAT TAT AAT CTT TTA GTT AGT TAT GCT TTA CGT TTA AGA GAA TTA ATA 
                                        =                                                   ^  


[201..220]
                E   N   A   V   S   L   R   L   P   R   R   I   S   K   I   F   Q   N   K   R  
MT753155.1     GAA AAT GCA GTG TCG TTG CGT CTT CCA AGA CGT ATT AGT AAA ATT TTC CAA AAT AAA CGA 
                E   N   A   V   S   L   R   L   P   R   R   I   S   K   I   F   Q   N   K   R  
MN938851.1     GAA AAT GCA GTG TCG TTG CGT CTT CCA AGA CGT ATT AGT AAA ATT TTC CAA AAT AAA CGA 
                Q   N   A   V   S   L   R   L   P   R   R   I   S   K   I   F   Q   K   K   R  
LdIV1_JGS      CAG AAT GCA GTT TCA TTA CGT CTT CCA AGA CGA ATT AGT AAA ATT TTT CAA AAG AAA CGA 
                Q   N   A   V   S   L   R   L   P   R   R   I   S   K   I   F   Q   K   K   R  
LdIV1_ZY       CAG AAT GCA GTT TCA TTA CGT CTT CCA AGA CGA ATT AGT AAA ATT TTT CAA AAG AAA CGA 
                E   N   A   V   S   L   R   L   P   R   R   I   S   K   I   F   Q   K   K   R  
LdIV1_NJ       GAG AAT GCA GTT TCA TTA CGT CTT CCA AGA CGA ATT AGT AAA ATT TTT CAA AAG AAA CGA 
                E   N   A   V   S   L   R   L   P   R   R   I   S   K   I   F   Q   K   K   R  
LdIV1_CT       GAG AAT GCA GTT TCA TTA CGT CTT CCA AGA CGA ATT AGT AAA ATT TTT CAA AAG AAA CGA 
                E   N   A   V   S   L   R   L   P   R   R   I   S   K   I   F   R   K   K   R  
KJ629170.1     GAG AAT GCA GTT TCG TTA CGT CTT CCA AGA CGA ATT AGT AAA ATC TTT CGA AAG AAA CGA 
                ^           =   =   =                   =               =   =   ^   ^          


[221..240]
                K   T   E   W   V   E   M   I   P   L   Q   Y   Q   G   G   T   P   A   A   P  
MT753155.1     AAG ACA GAA TGG GTT GAA ATG ATA CCA TTG CAA TAT CAG GGT GGT ACT CCT GCA GCG CCC 
                K   T   E   W   V   E   M   I   P   L   Q   Y   Q   G   G   T   P   A   A   P  
MN938851.1     AAG ACA GAA TGG GTT GAA ATG ATA CCA TTG CAA TAT CAG GGT GGT ACT CCT GCA GCG CCC 
                K   A   E   W   V   E   M   I   P   L   Q   Y   Q   G   G   T   P   A   A   P  
LdIV1_JGS      AAG GCA GAA TGG GTT GAG ATG ATA CCA TTG CAA TAT CAG GGT GGT ACT CCT GCG GCA CCA 
                K   A   E   W   V   E   M   I   P   L   Q   Y   Q   G   G   T   P   A   A   P  
LdIV1_ZY       AAG GCA GAA TGG GTT GAG ATG ATA CCA TTG CAA TAT CAG GGT GGT ACT CCT GCG GCA CCA 
                K   A   E   W   V   E   M   I   P   L   Q   Y   Q   G   G   T   P   A   A   P  
LdIV1_NJ       AAG GCA GAA TGG GTT GAG ATG ATA CCA TTG CAA TAT CAG GGT GGT ACT CCT GCG GCA CCA 
                K   A   E   W   V   E   M   I   P   L   Q   Y   Q   G   G   T   P   A   A   P  
LdIV1_CT       AAG GCA GAA TGG GTT GAG ATG ATA CCA TTG CAA TAT CAG GGT GGT ACT CCT GCG GCA CCA 
                K   A   E   W   V   E   M   I   P   L   Q   Y   Q   G   G   T   P   A   A   P  
KJ629170.1     AAG GCA GAA TGG GTT GAG ATG ATA CCA TTG CAA TAT CAA GGT GGT ACT CCT GCG GCA CCA 
                    ^               =                           =                   =   =   =  


[241..260]
                I   T   K   G   I   D   P   I   R   D   L   T   Q   D   D   N   R   P   K   C  
MT753155.1     ATA ACT AAA GGT ATA GAC CCT ATA CGA GAT CTT ACA CAG GAT GAT AAT AGA CCT AAG TGT 
                I   T   K   G   I   D   P   I   R   D   L   T   Q   D   D   N   R   P   K   C  
MN938851.1     ATA ACT AAA GGT ATA GAC CCT ATA CGA GAT CTT ACA CAG GAT GAT AAT AGA CCT AAG TGT 
                I   T   K   G   I   D   P   I   R   D   L   T   Q   D   D   N   R   P   K   C  
LdIV1_JGS      ATA ACT AAA GGT ATA GAT CCT ATA CGA GAT CTT ACA CAA GAT GAT AAT AGA CCA AAG TGT 
                I   T   K   G   I   D   P   I   R   D   L   T   Q   D   D   N   R   P   K   C  
LdIV1_ZY       ATA ACT AAA GGT ATA GAT CCT ATA CGA GAT CTT ACA CAA GAT GAT AAT AGA CCA AAG TGT 
                I   T   K   G   I   D   P   I   R   D   L   T   Q   D   D   N   R   P   K   C  
LdIV1_NJ       ATA ACT AAA GGT ATA GAT CCT ATA CGA GAT CTT ACA CAA GAC GAT AAT AGA CCA AAG TGT 
                I   T   K   G   I   D   P   I   R   D   L   T   Q   D   D   N   R   P   K   C  
LdIV1_CT       ATA ACT AAA GGT ATA GAT CCT ATA CGA GAT CTT ACA CAA GAT GAT AAT AGA CCA AAG TGT 
                L   T   K   G   I   D   P   I   R   D   L   T   Q   D   D   N   R   P   K   C  
KJ629170.1     CTA ACT AAA GGT ATA GAT CCT ATA CGA GAT CTT ACA CAA GAC GAT AAT AGA CCA AAG TGT 
                ^                   =                           =   =               =          


[261..280]
                C   A   Y   C   P   E   I   V   C   M   K   C   Y   K   G   R   G   C   C   E  
MT753155.1     TGT GCT TAC TGT CCT GAA ATT GTA TGT ATG AAG TGT TAT AAA GGT AGA GGG TGT TGT GAG 
                C   A   Y   C   P   E   I   V   C   M   K   C   Y   K   G   R   G   C   C   E  
MN938851.1     TGT GCT TAC TGT CCT GAA ATT GTA TGT ATG AAG TGT TAT AAA GGT AGA GGG TGT TGT GAG 
                C   A   Y   C   P   E   I   V   C   M   K   C   Y   K   G   R   G   C   C   E  
LdIV1_JGS      TGT GCT TAC TGC CCC GAA ATT GTA TGT ATG AAG TGT TAT AAA GGT AGA GGG TGT TGT GAA 
                C   A   Y   C   P   E   I   V   C   M   K   C   Y   K   G   R   G   C   C   E  
LdIV1_ZY       TGT GCT TAC TGC CCC GAA ATT GTA TGT ATG AAG TGT TAT AAA GGT AGA GGG TGT TGT GAA 
                C   A   Y   C   P   E   I   V   C   M   K   C   Y   K   G   R   G   C   C   E  
LdIV1_NJ       TGT GCT TAC TGC CCC GAA ATT GTA TGT ATG AAG TGT TAT AAA GGT AGA GGG TGT TGT GAA 
                C   A   Y   C   P   E   I   V   C   M   K   C   Y   K   G   R   G   C   C   E  
LdIV1_CT       TGT GCT TAC TGC CCC GAA ATT GTG TGT ATG AAG TGT TAT AAA GGT CGA GGG TGT TGT GAA 
                C   A   Y   C   P   A   I   V   C   M   K   C   Y   K   G   R   G   C   C   E  
KJ629170.1     TGT GCT TAC TGC CCC GCA ATT GTA TGT ATG AAG TGT TAC AAA GGT AGA GGG TGT TGT GAA 
                            =   =   ^       =                   =           =               =  


[281..300]
                C   F   P   H   Y   Q   G   N   V   E   Q   D   V   G   G   D   T   S   V   V  
MT753155.1     TGT TTT CCT CAT TAT CAA GGC AAC GTT GAA CAA GAT GTT GGT GGT GAT ACG AGT GTT GTA 
                C   F   P   H   Y   Q   G   N   V   E   Q   D   V   G   G   D   T   S   V   V  
MN938851.1     TGT TTT CCT CAT TAT CAA GGC AAC GTT GAA CAA GAT GTT GGT GGT GAT ACG AGT GTT GTA 
                C   F   P   H   Y   Q   G   N   V   E   Q   D   V   G   G   D   T   S   V   V  
LdIV1_JGS      TGT TTT CCT CAT TAT CAA GGC AAC GTT GAA CAA GAT GTT GGT GGT GAT ACG AGT GTT GTA 
                C   F   P   H   Y   Q   G   N   V   E   Q   D   V   G   G   D   T   S   V   V  
LdIV1_ZY       TGT TTT CCT CAT TAT CAA GGC AAC GTT GAA CAA GAT GTT GGT GGT GAT ACG AGT GTT GTA 
                C   F   P   H   Y   Q   G   N   V   E   Q   D   V   G   G   D   T   S   V   V  
LdIV1_NJ       TGT TTT CCT CAT TAT CAA GGC AAC GTT GAA CAA GAT GTT GGT GGT GAT ACG AGT GTT GTA 
                C   F   P   H   Y   Q   G   N   V   E   Q   D   V   G   G   D   T   S   V   V  
LdIV1_CT       TGT TTT CCT CAT TAT CAA GGC AAC GTT GAA CAA GAT GTT GGT GGT GAT ACG AGT GTT GTA 
                C   F   P   H   Y   Q   G   N   V   E   Q   G   V   G   G   D   T   S   V   V  
KJ629170.1     TGT TTT CCT CAT TAT CAA GGC AAC GTT GAA CAA GGT GTC GGT GGT GAT ACG AGT GTT GTA 
                                                            ^   =                              


[301..320]
                Q   I   H   K   A   H   N   V   V   L   T   E   T   E   I   T   Q   S   D   T  
MT753155.1     CAA ATT CAC AAA GCA CAT AAT GTT GTT CTT ACT GAA ACT GAA ATT ACG CAA TCT GAT ACT 
                Q   I   H   K   A   H   N   V   V   L   T   E   T   E   I   T   Q   S   D   T  
MN938851.1     CAA ATT CAC AAA GCA CAT AAT GTT GTT CTT ACT GAA ACT GAA ATT ACG CAA TCT GAT ACT 
                Q   I   H   K   A   H   N   V   V   L   T   E   T   E   I   T   Q   S   D   T  
LdIV1_JGS      CAA ATT CAT AAA GCG CAT AAT GTC GTT CTT ACT GAA ACT GAA ATT ACA CAA TCT GAT ACT 
                Q   I   H   K   A   H   N   V   V   L   T   E   T   E   I   T   Q   S   D   T  
LdIV1_ZY       CAA ATT CAT AAA GCG CAT AAT GTC GTT CTT ACT GAA ACT GAA ATT ACA CAA TCT GAT ACT 
                Q   I   H   K   A   H   N   V   V   L   T   E   T   E   I   T   Q   S   D   I  
LdIV1_NJ       CAA ATT CAT AAA GCG CAT AAT GTC GTT CTT ACT GAA ACT GAA ATT ACA CAA TCT GAT ATT 
                Q   I   H   K   A   H   N   V   V   L   T   E   T   E   I   T   Q   S   D   T  
LdIV1_CT       CAA ATT CAT AAA GCG CAT AAT GTC GTT CTT ACT GAA ACT GAA ATT ACA CAA TCT GAT ACT 
                Q   I   H   K   A   H   N   V   V   L   T   E   T   E   I   T   Q   S   D   T  
KJ629170.1     CAA ATT CAT AAA GCG CAT AAT GTC GTT CTT ACT GAA ACT GAA ATT ACA CAA TCT GAT ACT 
                        =       =           =                               =               ^  


[321..340]
                T   A   I   S   N   P   K   W   G   S   Y   V   S   S   D   T   I   S   Q   M  
MT753155.1     ACA GCT ATA TCA AAT CCA AAG TGG GGT AGT TAT GTA AGT TCC GAT ACT ATA TCT CAA ATG 
                T   A   I   S   N   P   K   W   G   S   Y   V   S   S   D   T   I   S   Q   M  
MN938851.1     ACA GCT ATA TCA AAT CCA AAG TGG GGT AGT TAT GTA AGT TCC GAT ACT ATA TCT CAA ATG 
                T   A   I   S   N   P   K   W   G   S   Y   V   S   S   D   T   I   S   Q   M  
LdIV1_JGS      ACA GCC ATA TCA AAC CCA AAG TGG GGT AGT TAT GTG AGT TCT GAT ACT ATA TCT CAG ATG 
                T   A   I   S   N   P   K   W   G   S   Y   V   S   S   D   T   I   S   Q   M  
LdIV1_ZY       ACA GCC ATA TCA AAC CCA AAG TGG GGT AGT TAT GTG AGT TCT GAT ACT ATA TCT CAG ATG 
                T   A   I   S   N   P   K   W   G   S   Y   V   S   S   D   T   I   S   Q   M  
LdIV1_NJ       ACA GCC ATA TCA AAC CCA AAG TGG GGT AGT TAT GTG AGT TCT GAT ACT ATA TCT CAG ATG 
                T   A   I   S   N   P   K   W   G   S   Y   V   S   S   D   T   I   S   Q   M  
LdIV1_CT       ACA GCC ATA TCA AAC CCA AAG TGG GGT AGT TAT GTG AGT TCT GAT ACT ATA TCT CAG ATG 
                T   A   I   S   N   P   K   W   G   S   Y   V   S   S   D   T   I   S   Q   M  
KJ629170.1     ACA GCC ATA TCA AAC CCA AAG TGG GGT AGT TAT GTG AGT TCT GAT ACT ATA TCT CAG ATG 
                    =           =                           =       =                   =      


[341..360]
                D   T   L   V   N   R   W   F   R   V   G   T   Y   T   W   T   T   Q   M   N  
MT753155.1     GAT ACG TTA GTT AAC AGA TGG TTT CGT GTT GGT ACT TAT ACG TGG ACT ACC CAG ATG AAT 
                D   T   L   V   N   R   W   F   R   V   G   T   Y   T   W   T   T   Q   M   N  
MN938851.1     GAT ACG TTA GTT AAC AGA TGG TTT CGT GTT GGT ACT TAT ACG TGG ACT ACC CAG ATG AAT 
                D   T   L   V   N   R   W   F   R   V   G   T   Y   T   W   T   T   Q   M   N  
LdIV1_JGS      GAT ACA TTA GTT AAT AGA TGG TTT CGT GTT GGT ACT TAT ACG TGG ACT ACC CAG ATG AAT 
                D   T   L   V   N   R   W   F   R   V   G   T   Y   T   W   T   T   Q   M   N  
LdIV1_ZY       GAT ACA TTA GTT AAT AGA TGG TTT CGT GTT GGT ACT TAT ACG TGG ACT ACC CAG ATG AAT 
                D   T   L   V   N   R   W   F   R   V   G   T   Y   T   W   T   T   Q   M   N  
LdIV1_NJ       GAT ACA TTA GTT AAT AGA TGG TTT CGT GTT GGT ACT TAT ACG TGG ACT ACC CAG ATG AAT 
                D   T   L   V   N   R   W   F   R   V   G   T   Y   T   W   T   T   Q   M   N  
LdIV1_CT       GAT ACA TTA GTT AAT AGA TGG TTT CGT GTT GGT ACT TAT ACG TGG ACT ACC CAG ATG AAT 
                D   T   L   V   N   R   W   F   R   V   G   T   Y   T   W   T   T   Q   M   N  
KJ629170.1     GAT ACA TTA GTT AAC AGA TGG TTT CGT GTT GGY ACT TAT ACG TGG ACT ACC CAG ATG AAT 
                    =           =                       =                                      


[361..380]
                R   N   T   T   I   R   S   I   S   L   P   R   D   A   V   F   S   G   S   T  
MT753155.1     CGC AAT ACT ACT ATT AGA TCT ATT AGC TTA CCC AGA GAT GCT GTT TTT TCT GGC AGT ACT 
                R   N   T   T   I   R   S   I   S   L   P   R   D   A   V   F   S   G   S   T  
MN938851.1     CGC AAT ACT ACT ATT AGA TCT ATT AGC TTA CCC AGA GAT GCT GTT TTT TCT GGC AGT ACT 
                R   N   T   T   I   R   S   I   S   L   P   R   D   A   V   F   S   G   S   T  
LdIV1_JGS      CGT AAT ACT ACT ATT AGA TCT ATT AGT TTA CCT AGA GAT GCT GTT TTT TCT GGC AGC ACT 
                R   N   T   T   I   R   S   I   S   L   P   R   D   A   V   F   S   G   S   T  
LdIV1_ZY       CGT AAT ACT ACT ATT AGA TCT ATT AGT TTA CCT AGA GAT GCT GTT TTT TCT GGC AGC ACT 
                R   N   T   T   I   R   S   I   S   L   P   R   D   A   V   F   S   G   S   T  
LdIV1_NJ       CGT AAT ACT ACT ATT AGA TCT ATT AGT TTA CCT AGA GAT GCT GTT TTT TCT GGC AGC ACT 
                R   N   T   T   I   R   S   I   S   L   P   R   D   A   V   F   S   G   S   T  
LdIV1_CT       CGT AAT ACT ACT ATT AGA TCT ATT AGT TTA CCT AGA GAT GCT GTT TTT TCT GGC AGC ACT 
                R   N   T   T   I   K   S   I   S   L   P   R   D   A   V   F   S   G   S   T  
KJ629170.1     CGT AAT ACT ACT ATT AAA TCT ATT AGT TTA CCT AGA GAT GCT GTT TTT TCT GGC AGC ACT 
                =                   ^           =       =                               =      


[381..400]
                T   C   D   Q   P   N   K   I   P   F   R   I   H   R   Y   W   R   G   D   I  
MT753155.1     ACA TGT GAT CAG CCA AAT AAA ATT CCG TTC CGT ATA CAT AGA TAT TGG CGA GGA GAT ATT 
                T   C   D   Q   P   N   K   I   P   F   R   I   H   R   Y   W   R   G   D   I  
MN938851.1     ACA TGT GAT CAG CCA AAT AAA ATT CCG TTC CGT ATA CAT AGA TAT TGG CGA GGA GAT ATT 
                T   C   D   Q   P   N   K   I   P   F   R   I   H   R   Y   W   R   G   D   I  
LdIV1_JGS      ACG TGT GAT CAG CCA AAT AAA ATC CCA TTC CGT ATA CAT AGA TAT TGG CGA GGA GAT ATT 
                T   C   D   Q   P   N   K   I   P   F   R   I   H   R   Y   W   R   G   D   I  
LdIV1_ZY       ACG TGT GAT CAG CCA AAT AAA ATC CCA TTC CGT ATA CAT AGA TAT TGG CGA GGA GAT ATT 
                T   C   D   Q   P   N   K   I   P   F   R   I   H   R   Y   W   R   G   D   I  
LdIV1_NJ       ACA TGT GAT CAG CCA AAT AAG ATC CCA TTC CGT ATA CAT AGA TAT TGG CGA GGA GAT ATT 
                T   C   D   Q   P   N   K   I   P   F   R   I   H   R   Y   W   R   G   D   I  
LdIV1_CT       ACA TGT GAT CAG CCA AAT AAA ATC CCA TTC CGT ATA CAT AGA TAT TGG CGA GGA GAT ATT 
                T   C   D   Q   P   N   K   I   P   F   R   I   H   R   Y   W   R   G   D   I  
KJ629170.1     ACA TGT GAT CAG CCA AAT AAG ATC CCA TTC CGT ATA CAT AGA TAT TGG CGA GGA GAT ATT 
                =                       =   =   =                                              


[401..420]
                T   V   K   I   H   I   N   C   N   K   F   Q   I   G   Q   L   Q   C   S   W  
MT753155.1     ACT GTT AAA ATT CAT ATA AAC TGT AAT AAA TTT CAA ATA GGC CAA CTG CAA TGT AGC TGG 
                T   V   K   I   H   I   N   C   N   K   F   Q   I   G   Q   L   Q   C   S   W  
MN938851.1     ACT GTT AAA ATT CAT ATA AAC TGT AAT AAA TTT CAA ATA GGC CAA CTG CAA TGT AGC TGG 
                T   V   K   I   H   I   N   C   N   K   F   Q   I   G   Q   L   Q   C   S   W  
LdIV1_JGS      ACT GTT AAA ATT CAT ATA AAT TGC AAT AAA TTT CAA ATA GGC CAA TTA CAA TGT AGT TGG 
                T   V   K   I   H   I   N   C   N   K   F   Q   I   G   Q   L   Q   C   S   W  
LdIV1_ZY       ACT GTT AAA ATT CAT ATA AAT TGC AAT AAA TTT CAA ATA GGC CAA TTA CAA TGT AGT TGG 
                T   V   K   I   H   I   N   C   N   K   F   Q   I   G   Q   L   Q   C   S   W  
LdIV1_NJ       ACT GTT AAA ATT CAT ATA AAT TGC AAT AAA TTT CAA ATA GGC CAA TTA CAA TGT AGT TGG 
                T   V   K   I   H   I   N   C   N   K   F   Q   I   G   Q   L   Q   C   S   W  
LdIV1_CT       ACT GTT AAA ATT CAT ATA AAT TGC AAT AAA TTT CAA ATA GGC CAA TTA CAA TGT AGT TGG 
                T   V   K   I   H   I   N   C   N   K   F   Q   I   G   Q   L   Q   C   S   W  
KJ629170.1     ACT GTT AAA ATT CAT ATA AAT TGC AAT AAA TTT CAA ATA GGC CAA TTA CAG TGT AGT TGG 
                                        =   =                               =   =       =      


[421..440]
                Y   Y   Q   P   K   A   D   D   S   F   A   S   K   S   S   V   Y   T   R   S  
MT753155.1     TAT TAT CAA CCA AAA GCA GAT GAT TCA TTT GCT AGT AAG AGT AGT GTA TAT ACA CGA AGT 
                Y   Y   Q   P   K   A   D   D   S   F   A   S   K   S   S   V   Y   T   R   S  
MN938851.1     TAT TAT CAA CCA AAA GCA GAT GAT TCA TTT GCT AGT AAG AGT AGT GTA TAT ACA CGA AGT 
                Y   Y   Q   P   K   A   D   D   S   F   A   S   K   N   S   V   Y   T   R   S  
LdIV1_JGS      TAT TAT CAA CCA AAA GCA GAT GAT TCA TTT GCT AGT AAG AAT AGT GTA TAT ACA CGA AGT 
                Y   Y   Q   P   K   A   D   D   S   F   A   S   K   N   S   V   Y   T   R   S  
LdIV1_ZY       TAT TAT CAA CCA AAA GCA GAT GAT TCA TTT GCT AGT AAG AAT AGT GTA TAT ACA CGA AGT 
                Y   Y   Q   P   K   A   D   D   S   F   A   S   K   N   S   V   Y   T   R   S  
LdIV1_NJ       TAT TAT CAA CCA AAA GCA GAT GAT TCA TTT GCT AGT AAG AAT AGT GTA TAT ACA CGA AGT 
                Y   Y   Q   P   K   A   D   D   S   F   A   S   K   N   S   V   Y   T   R   S  
LdIV1_CT       TAT TAT CAA CCA AAA GCA GAT GAT TCA TTT GCT AGT AAG AAT AGT GTA TAT ACA CGA AGT 
                Y   Y   Q   P   K   A   D   D   S   F   A   S   K   N   S   V   Y   T   R   S  
KJ629170.1     TAT TAT CAA CCA AAA GCA GAT GAT TCA TTT GCT AGT AAG AAT AGT GTA TAT ACA CGA AGT 
                                                                    ^                          


[441..460]
                G   T   H   H   C   V   I   S   A   A   P   N   N   E   V   E   L   R   I   P  
MT753155.1     GGC ACT CAT CAT TGT GTT ATT TCG GCA GCC CCT AAC AAT GAG GTA GAG CTT CGT ATT CCA 
                G   T   H   H   C   V   I   S   A   A   P   N   N   E   V   E   L   R   I   P  
MN938851.1     GGC ACT CAT CAT TGT GTT ATT TCG GCA GCC CCT AAC AAT GAG GTA GAG CTT CGT ATT CCA 
                G   T   H   H   C   V   I   S   A   A   P   N   N   E   V   E   L   R   I   P  
LdIV1_JGS      GGT ACT CAT CAT TGT GTT ATT TCA GCA GCC CCT AAT AAC GAG GTA GAG CTC CGT ATT CCC 
                G   T   H   H   C   V   I   S   A   A   P   N   N   E   V   E   L   R   I   P  
LdIV1_ZY       GGT ACT CAT CAT TGT GTT ATT TCA GCA GCC CCT AAT AAC GAG GTA GAG CTC CGT ATT CCC 
                G   T   H   H   C   V   I   S   A   A   P   N   N   E   V   E   L   R   I   P  
LdIV1_NJ       GGT ACT CAT CAT TGT GTT ATT TCA GCA GCC CCT AAT AAC GAG GTA GAG CTC CGT ATT CCC 
                G   T   H   H   C   V   I   S   A   A   P   N   N   E   V   E   L   R   I   P  
LdIV1_CT       GGT ACT CAT CAT TGT GTT ATT TCA GCA GCC CCT AAT AAC GAG GTA GAG CTC CGT ATT CCC 
                G   T   H   H   C   V   I   S   A   A   P   N   N   E   V   E   L   R   I   P  
KJ629170.1     GGT ACT CAT CAT TGT GTT ATT TCA GCA GCC CCT AAT AAC GAG GTA GAG CTC CGT ATT CCC 
                =                           =               =   =               =           =  


[461..480]
                Y   K   A   Y   K   S   M   Y   H   T   K   T   Y   N   G   D   V   K   D   L  
MT753155.1     TAT AAA GCA TAT AAA AGT ATG TAT CAC ACT AAA ACG TAT AAT GGC GAT GTT AAA GAT CTT 
                Y   K   A   Y   K   S   M   Y   H   T   K   T   Y   N   G   D   V   K   D   L  
MN938851.1     TAT AAA GCA TAT AAA AGT ATG TAT CAC ACT AAA ACG TAT AAT GGC GAT GTT AAA GAT CTT 
                Y   K   A   Y   K   S   M   Y   H   T   K   T   Y   N   G   D   V   K   D   L  
LdIV1_JGS      TAT AAA GCA TAT AAA AGT ATG TAT CAT ACT AAA ACA TAT AAT GGC GAT GTC AAA GAT CTT 
                Y   K   A   Y   K   S   M   Y   H   T   K   T   Y   N   G   D   V   K   D   L  
LdIV1_ZY       TAT AAA GCA TAT AAA AGT ATG TAT CAT ACT AAA ACA TAT AAT GGC GAT GTC AAA GAT CTT 
                Y   K   A   Y   K   S   M   Y   H   T   K   T   Y   N   G   D   V   K   D   L  
LdIV1_NJ       TAT AAA GCA TAT AAA AGT ATG TAT CAT ACT AAA ACA TAT AAT GGC GAT GTC AAA GAT CTT 
                Y   K   A   Y   K   S   M   Y   H   T   K   T   Y   N   G   D   V   K   D   L  
LdIV1_CT       TAT AAA GCA TAT AAA AGT ATG TAT CAT ACT AAA ACA TAT AAT GGC GAT GTC AAA GAT CTT 
                Y   K   A   Y   K   S   M   Y   H   T   K   T   Y   N   G   D   V   K   D   L  
KJ629170.1     TAT AAA GCA TAT AAA AGT ATG TAT CAT ACT AAA ACA TAT AAT GGC GAT GTC AAA GAT CTT 
                                                =           =                   =              


[481..500]
                P   L   D   M   G   T   L   F   I   S   V   L   S   P   L   K   T   T   G   E  
MT753155.1     CCG CTG GAT ATG GGG ACG TTA TTT ATA AGC GTA TTA AGT CCA TTG AAA ACG ACT GGA GAA 
                P   L   D   M   G   T   L   F   I   S   V   L   S   P   L   K   T   T   G   E  
MN938851.1     CCG CTG GAT ATG GGG ACG TTA TTT ATA AGC GTA TTA AGT CCA TTG AAA ACG ACT GGA GAA 
                P   L   D   M   G   T   L   F   I   S   V   L   S   P   L   K   T   T   G   E  
LdIV1_JGS      CCG CTT GAT ATG GGA ACA CTG TTT ATA AGC GTG TTA AGT CCG TTG AAA ACG ACT GGA GAA 
                P   L   D   M   G   T   L   F   I   S   V   L   S   P   L   K   T   T   G   E  
LdIV1_ZY       CCG CTT GAT ATG GGA ACA CTG TTT ATA AGC GTG TTA AGT CCG TTG AAA ACG ACT GGA GAA 
                P   L   D   M   G   T   L   F   I   S   V   L   S   P   L   K   T   T   G   E  
LdIV1_NJ       CCG CTT GAT ATG GGA ACA CTG TTT ATA AGC GTG TTA AGT CCG TTG AAA ACG ACT GGA GAA 
                P   L   D   M   G   T   L   F   I   S   V   L   S   P   L   K   T   T   G   E  
LdIV1_CT       CCG CTT GAT ATG GGA ACA CTG TTT ATA AGC GTG TTA AGT CCG TTG AAA ACG ACT GGA GAA 
                P   L   D   M   G   T   L   F   I   S   V   L   S   P   L   K   T   T   G   E  
KJ629170.1     CCG CTC GAT ATG GGA ACA CTG TTT ATA AGC GTG TTA AGT CCG TTG AAA ACG ACT GGA GAA 
                    =           =   =   =               =           =                          


[501..520]
                T   S   P   R   C   S   F   T   V   F   V   K   Y   E   N   N   E   F   T   G  
MT753155.1     ACT TCT CCT AGG TGT AGT TTT ACC GTT TTT GTT AAA TAT GAA AAT AAT GAA TTC ACT GGT 
                T   S   P   R   C   S   F   T   V   F   V   K   Y   E   N   N   E   F   T   G  
MN938851.1     ACT TCT CCT AGG TGT AGT TTT ACC GTT TTT GTT AAA TAT GAA AAT AAT GAA TTC ACT GGT 
                T   S   P   R   C   S   F   T   V   F   V   K   Y   E   N   N   E   F   T   G  
LdIV1_JGS      ACA TCT CCT AGA TGT AGT TTT ACT GTT TTT GTT AAG TAT GAA AAT AAT GAA TTT ACT GGT 
                T   S   P   R   C   S   F   T   V   F   V   K   Y   E   N   N   E   F   T   G  
LdIV1_ZY       ACA TCT CCT AGA TGT AGT TTT ACT GTT TTT GTT AAG TAT GAA AAT AAT GAA TTT ACT GGT 
                T   S   P   R   C   S   F   T   V   F   V   K   Y   E   N   N   E   F   T   G  
LdIV1_NJ       ACA TCT CCT AGA TGT AGT TTT ACT GTT TTT GTT AAG TAT GAA AAT AAT GAA TTT ACT GGT 
                T   S   P   R   C   S   F   T   V   F   V   K   Y   E   N   N   E   F   T   G  
LdIV1_CT       ACA TCT CCT AGA TGT AGT TTT ACT GTT TTT GTT AAG TAT GAA AAT AAT GAA TTT ACT GGT 
                T   S   P   R   C   S   F   T   V   F   V   K   Y   E   N   N   E   F   T   G  
KJ629170.1     ACA TCT CCT AGA TGT AGT TTT ACT GTT TTT GTT AAG TAT GAA AAT AAT GAA TTT ACT GGT 
                =           =               =               =                       =          


[521..540]
                M   I   A   G   D   V   D   T   P   S   Q   V   S   E   N   L   Q   Y   Q   M  
MT753155.1     ATG ATT GCT GGA GAT GTT GAT ACT CCT TCC CAA GTT TCT GAA AAT TTA CAA TAT CAA ATG 
                M   I   A   G   D   V   D   T   P   S   Q   V   S   E   N   L   Q   Y   Q   M  
MN938851.1     ATG ATT GCT GGA GAT GTT GAT ACT CCT TCC CAA GTT TCT GAA AAT TTA CAA TAT CAA ATG 
                M   I   A   G   D   V   D   T   P   S   Q   V   S   E   S   L   Q   Y   Q   M  
LdIV1_JGS      ATG ATT GCT GGA GAT GTT GAT ACC CCT TCC CAA GTT TCT GAA AGT TTA CAA TAT CAA ATG 
                M   I   A   G   D   V   D   T   P   S   Q   V   S   E   S   L   Q   Y   Q   M  
LdIV1_ZY       ATG ATT GCT GGA GAT GTT GAT ACC CCT TCC CAA GTT TCT GAA AGT TTA CAA TAT CAA ATG 
                M   I   A   G   D   V   D   T   P   S   Q   V   S   E   S   L   Q   Y   Q   M  
LdIV1_NJ       ATG ATT GCT GGA GAT GTT GAT ACC CCT TCC CAA GTT TCT GAA AGT TTA CAA TAT CAA ATG 
                M   I   A   G   D   V   D   T   P   S   Q   V   S   E   S   L   Q   Y   Q   M  
LdIV1_CT       ATG ATT GCT GGA GAT GTT GAT ACC CCT TCC CAA GTT TCT GAA AGT TTA CAA TAT CAA ATG 
                M   I   A   G   D   V   D   T   P   S   Q   V   S   E   S   L   Q   Y   Q   M  
KJ629170.1     ATG ATT GCT GGA GAT GTT GAT ACC CCT TCC CAA GTT TCT GAA AGT TTA CAA TAT CAA ATG 
                                            =                           ^                      


[541..560]
                D   G   I   G   S   I   L   S   A   A   V   P   V   V   E   K   L   L   T   S  
MT753155.1     GAT GGT ATA GGA TCT ATT TTA TCT GCA GCT GTT CCG GTT GTA GAG AAA TTA TTG ACT AGT 
                D   G   I   G   S   I   L   S   A   A   V   P   V   V   E   K   L   L   T   S  
MN938851.1     GAT GGT ATA GGA TCT ATT TTA TCT GCA GCT GTT CCG GTT GTA GAG AAA TTA TTG ACT AGT 
                D   G   I   G   S   I   L   S   A   A   V   P   V   V   E   K   L   L   T   S  
LdIV1_JGS      GAT GGT ATA GGA TCT ATT TTA TCT GCA GCC GTT CCA GTT GTA GAG AAA TTA TTG ACT AGT 
                D   G   I   G   S   I   L   S   A   A   V   P   V   V   E   K   L   L   T   S  
LdIV1_ZY       GAT GGT ATA GGA TCT ATT TTA TCT GCA GCC GTT CCA GTT GTA GAG AAA TTA TTG ACT AGT 
                D   G   I   G   S   I   L   S   A   A   V   P   V   V   E   K   L   L   T   S  
LdIV1_NJ       GAT GGT ATA GGA TCT ATT TTA TCT GCA GCC GTT CCA GTT GTA GAG AAA TTA TTG ACT AGT 
                D   G   I   G   S   I   L   S   A   A   V   P   V   V   E   K   L   L   T   S  
LdIV1_CT       GAT GGT ATA GGA TCT ATT TTA TCT GCA GCC GTT CCA GTT GTA GAG AAA TTA TTG ACT AGT 
                D   G   M   G   S   I   L   S   A   A   V   P   V   V   E   K   L   L   T   S  
KJ629170.1     GAT GGT ATG GGA TCT ATT TTA TCT GCA GCC GTT CCA GTT GTA GAG AAA TTA TTG ACT AGT 
                        ^                           =       =                                  


[561..580]
                S   P   N   D   N   N   R   D   N   P   P   L   N   S   A   P   R   Y   F   V  
MT753155.1     AGT CCT AAT GAT AAT AAT CGT GAT AAT CCT CCT CTA AAT TCA GCT CCA CGA TAT TTT GTA 
                S   P   N   D   N   N   R   D   N   P   P   L   N   S   A   P   R   Y   F   V  
MN938851.1     AGT CCT AAT GAT AAT AAT CGT GAT AAT CCT CCT CTA AAT TCA GCT CCA CGA TAT TTT GTA 
                S   P   N   D   N   N   R   D   N   P   P   L   N   S   A   P   R   Y   F   V  
LdIV1_JGS      AGT CCT AAT GAT AAT AAC CGT GAT AAT CCT CCT CTA AAT TCA GCT CCT CGG TAT TTT GTA 
                S   P   N   D   N   N   R   D   N   P   P   L   N   S   A   P   R   Y   F   V  
LdIV1_ZY       AGT CCT AAT GAT AAT AAC CGT GAT AAT CCT CCT CTA AAT TCA GCT CCT CGG TAT TTT GTA 
                S   P   N   D   N   N   R   D   N   P   P   L   N   S   A   P   R   Y   F   V  
LdIV1_NJ       AGT CCT AAT GAT AAT AAC CGT GAT AAT CCT CCT CTA AAT TCA GCT CCT CGG TAT TTT GTA 
                S   P   N   D   N   N   R   D   N   P   P   L   N   S   A   P   R   Y   F   V  
LdIV1_CT       AGT CCT AAT GAT AAT AAC CGT GAT AAT CCT CCT CTA AAT TCA GCT CCT CGG TAT TTT GTA 
                S   P   N   D   N   N   R   D   N   P   P   L   N   S   A   P   R   Y   F   V  
KJ629170.1     AGT CCT AAT GAT AAT AAC CGT GAT AAT CCT CCT CTA AAT TCA GCT CCT CGG TAT TTT GTA 
                                    =                                       =   =              


[581..600]
                P   T   A   S   H   S   W   S   I   G   T   D   L   V   E   P   L   H   N   L  
MT753155.1     CCG ACC GCG AGT CAT TCA TGG TCA ATT GGT ACA GAT TTA GTA GAA CCC CTT CAT AAT TTA 
                P   T   A   S   H   S   W   S   I   G   T   D   L   V   E   P   L   H   N   L  
MN938851.1     CCG ACC GCG AGT CAT TCA TGG TCA ATT GGT ACA GAT TTA GTA GAA CCC CTT CAT AAT TTA 
                P   T   A   S   H   S   W   S   I   G   T   D   L   V   E   P   L   H   N   L  
LdIV1_JGS      CCA ACT GCA AGT CAT TCA TGG TCA ATT GGT ACA GAT TTA GTA GAA CCC CTT CAT AAT TTA 
                P   T   A   S   H   S   W   S   I   G   T   D   L   V   E   P   L   H   N   L  
LdIV1_ZY       CCA ACT GCA AGT CAT TCA TGG TCA ATT GGT ACA GAT TTA GTA GAA CCC CTT CAT AAT TTA 
                P   T   A   S   H   S   W   S   I   G   T   D   L   V   E   P   L   H   N   L  
LdIV1_NJ       CCA ACT GCA AGT CAT TCA TGG TCA ATT GGT ACA GAT TTA GTA GAA CCC CTT CAT AAT TTA 
                P   T   A   S   H   S   W   S   I   G   T   D   L   V   E   P   L   H   N   L  
LdIV1_CT       CCA ACT GCA AGT CAT TCA TGG TCA ATT GGT ACA GAT TTA GTA GAA CCC CTT CAT AAT TTA 
                P   T   A   S   H   S   W   S   V   G   T   D   L   V   E   P   L   H   N   L  
KJ629170.1     CCA ACT GCA AGT CAT TCA TGG TCA GTT GGT ACA GAT TTA GTA GAA CCC CTT CAT AAT TTA 
                =   =   =                       ^                                              


[601..620]
                R   L   S   G   R   A   Q   T   R   H   P   D   V   D   L   D   E   M   K   V  
MT753155.1     CGC CTA AGC GGA CGA GCT CAG ACA CGT CAT CCG GAT GTA GAT TTG GAT GAA ATG AAA GTA 
                R   L   S   G   R   A   Q   T   R   H   P   D   V   D   L   D   E   M   K   V  
MN938851.1     CGC CTA AGC GGA CGA GCT CAG ACA CGT CAT CCG GAT GTA GAT TTG GAT GAA ATG AAA GTA 
                R   L   S   G   R   A   Q   T   R   H   P   D   V   D   L   D   E   M   K   V  
LdIV1_JGS      CGC TTG AGT GGG CGA GCT CAA ACA CGT CAT CCG GAT GTG GAT TTA GAT GAG ATG AAA GTA 
                R   L   S   G   R   A   Q   T   R   H   P   D   V   D   L   D   E   M   K   V  
LdIV1_ZY       CGC TTG AGT GGG CGA GCT CAA ACA CGT CAT CCG GAT GTG GAT TTA GAT GAG ATG AAA GTA 
                R   L   S   G   R   A   Q   T   R   H   P   D   V   D   L   D   E   M   K   V  
LdIV1_NJ       CGC TTG AGT GGG CGA GCT CAA ACA CGT CAT CCG GAT GTG GAT TTA GAT GAG ATG AAA GTA 
                R   L   S   G   R   A   Q   T   R   H   P   D   V   D   L   D   E   M   K   V  
LdIV1_CT       CGC TTG AGT GGG CGA GCT CAA ACA CGT CAT CCG GAT GTG GAT TTA GAT GAG ATG AAA GTA 
                R   L   S   G   R   A   Q   T   R   H   P   D   V   D   L   D   E   M   K   V  
KJ629170.1     CGC TTG AGT GGG CGA GCT CAA ACA CGT CAT CCG GAT GTG GAT TTA GAT GAG ATG AAA GTA 
                    =   =   =           =                       =       =       =              


[621..640]
                D   V   L   K   R   K   Y   M   L   C   D   V   F   S   W   S   Q   Q   D   P  
MT753155.1     GAT GTT TTG AAG CGT AAA TAT ATG TTG TGT GAC GTA TTT TCA TGG TCA CAA CAA GAT CCT 
                D   V   L   K   R   K   Y   M   L   C   D   V   F   S   W   S   Q   Q   D   P  
MN938851.1     GAT GTT TTG AAG CGT AAA TAT ATG TTG TGT GAC GTA TTT TCA TGG TCA CAA CAA GAT CCT 
                D   V   L   K   R   K   Y   M   L   C   D   V   F   S   W   S   Q   Q   D   P  
LdIV1_JGS      GAT GTT CTG AAG CGT AAA TAT ATG TTG TGT GAT GTA TTT TCA TGG TCG CAA CAA GAT CCT 
                D   V   L   K   R   K   Y   M   L   C   D   V   F   S   W   S   Q   Q   D   P  
LdIV1_ZY       GAT GTT CTG AAG CGT AAA TAT ATG TTG TGT GAT GTA TTT TCA TGG TCG CAA CAA GAT CCT 
                D   V   L   K   R   K   Y   M   L   C   D   V   F   S   W   S   Q   Q   D   P  
LdIV1_NJ       GAT GTT CTG AAG CGT AAA TAT ATG TTG TGT GAT GTA TTT TCA TGG TCG CAA CAA GAT CCT 
                D   V   L   K   R   K   Y   M   L   C   D   V   F   S   W   S   Q   Q   D   P  
LdIV1_CT       GAT GTT CTG AAG CGT AAA TAT ATG TTG TGT GAT GTA TTT TCA TGG TCG CAA CAA GAT CCT 
                D   V   L   K   R   K   Y   M   L   C   D   V   F   S   W   S   Q   Q   D   P  
KJ629170.1     GAT GTT CTG AAG CGT AAG TAT ATG TTG TGT GAT GTA TTT TCA TGG TCG CAA CAA GAT CCT 
                        =           =                   =                   =                  


[641..660]
                N   G   H   N   L   W   N   F   P   V   N   P   M   P   P   K   D   R   L   Y  
MT753155.1     AAT GGC CAT AAT CTA TGG AAT TTT CCT GTT AAT CCA ATG CCA CCT AAA GAT CGA TTA TAT 
                N   G   H   N   L   W   N   F   P   V   N   P   M   P   P   K   D   R   L   Y  
MN938851.1     AAT GGC CAT AAT CTA TGG AAT TTT CCT GTT AAT CCA ATG CCA CCT AAA GAT CGA TTA TAT 
                N   G   H   N   L   W   N   F   P   V   N   P   M   P   P   K   D   R   L   Y  
LdIV1_JGS      AAT GGC CAT AAT TTG TGG AAT TTC CCT GTT AAT CCA ATG CCA CCT AAA GAT CGA TTG TAT 
                N   G   H   N   L   W   N   F   P   V   N   P   M   P   P   K   D   R   L   Y  
LdIV1_ZY       AAT GGC CAT AAT TTG TGG AAT TTC CCT GTT AAT CCA ATG CCA CCT AAA GAT CGA TTG TAT 
                N   G   H   N   L   W   N   F   P   V   N   P   M   P   P   K   D   R   L   Y  
LdIV1_NJ       AAT GGC CAT AAT TTG TGG AAT TTC CCT GTT AAT CCA ATG CCA CCT AAA GAT CGA TTG TAT 
                N   G   H   N   L   W   N   F   P   V   N   P   M   P   P   K   D   R   L   Y  
LdIV1_CT       AAT GGC CAT AAT TTG TGG AAT TTC CCT GTT AAT CCA ATG CCA CCT AAA GAT CGA TTG TAT 
                N   G   H   N   L   W   N   F   P   V   N   P   M   P   P   K   D   R   L   Y  
KJ629170.1     AAT GGC CAT AAT TTG TGG AAT TTC CCT GTT AAT CCA ATG CCA CCT AAA GAT CGA TTG TAT 
                                =           =                                           =      


[661..680]
                K   V   A   Q   A   G   T   N   T   L   S   K   Y   Q   I   T   P   I   G   F  
MT753155.1     AAA GTT GCT CAA GCC GGC ACT AAT ACA TTA TCT AAA TAT CAA ATT ACG CCT ATA GGT TTC 
                K   V   A   Q   A   G   T   N   T   L   S   K   Y   Q   I   T   P   I   G   F  
MN938851.1     AAA GTT GCT CAA GCC GGC ACT AAT ACA TTA TCT AAA TAT CAA ATT ACG CCT ATA GGT TTC 
                K   V   A   Q   A   G   T   N   T   L   S   K   Y   Q   I   T   P   I   G   F  
LdIV1_JGS      AAA GTT GCT CAA GCC GGT ACC AAT ACA TTA TCT AAA TAT CAG ATT ACG CCT ATA GGT TTC 
                K   V   A   Q   A   G   T   N   T   L   S   K   Y   Q   I   T   P   I   G   F  
LdIV1_ZY       AAA GTT GCT CAA GCC GGT ACC AAT ACA TTA TCT AAA TAT CAG ATT ACG CCT ATA GGT TTC 
                K   V   A   Q   A   G   T   N   T   L   S   K   Y   Q   I   T   P   I   G   F  
LdIV1_NJ       AAA GTT GCT CAA GCC GGT ACC AAT ACA TTA TCT AAA TAT CAG ATT ACG CCT ATA GGT TTC 
                K   V   A   Q   A   G   T   N   T   L   S   K   Y   Q   I   T   P   I   G   F  
LdIV1_CT       AAA GTT GCT CAA GCC GGT ACC AAT ACA TTA TCT AAA TAT CAG ATT ACG CCT ATA GGT TTC 
                K   V   A   Q   A   G   T   N   T   L   S   K   Y   Q   I   T   P   I   G   F  
KJ629170.1     AAA GTT GCT CAA GCC GGT ACC AAT ACA TTA TCT AAA TAT CAG ATT ACG CCT ATA GGT TTC 
                                    =   =                           =                          


[681..700]
                L   S   S   L   Y   Q   Y   W   R   G   S   I   E   Y   R   F   D   I   V   A  
MT753155.1     TTA AGT AGT TTA TAT CAA TAT TGG CGT GGT TCT ATT GAA TAT AGA TTT GAT ATA GTA GCT 
                L   S   S   L   Y   Q   Y   W   R   G   S   I   E   Y   R   F   D   I   V   A  
MN938851.1     TTA AGT AGT TTA TAT CAA TAT TGG CGT GGT TCT ATT GAA TAT AGA TTT GAT ATA GTA GCT 
                L   S   S   L   Y   Q   Y   W   R   G   S   I   E   Y   R   F   D   I   V   A  
LdIV1_JGS      TTA AGT AGT TTA TAT CAA TAT TGG CGT GGT TCT ATC GAA TAT AGA TTT GAT ATA GTA GCT 
                L   S   S   L   Y   Q   Y   W   R   G   S   I   E   Y   R   F   D   I   V   A  
LdIV1_ZY       TTA AGT AGT TTA TAT CAA TAT TGG CGT GGT TCT ATC GAA TAT AGA TTT GAT ATA GTA GCT 
                L   S   S   L   Y   Q   Y   W   R   G   S   I   E   Y   R   F   D   I   V   A  
LdIV1_NJ       TTA AGT AGT TTA TAT CAA TAT TGG CGT GGT TCT ATC GAA TAT AGA TTT GAT ATA GTA GCT 
                L   S   S   L   Y   Q   Y   W   R   G   S   I   E   Y   R   F   D   I   V   A  
LdIV1_CT       TTA AGT AGT TTA TAT CAA TAT TGG CGT GGT TCT ATT GAA TAT AGA TTT GAT ATA GTA GCT 
                L   S   S   L   Y   Q   Y   W   R   G   S   I   E   Y   R   F   D   I   V   A  
KJ629170.1     TTA AGT AGT TTA TAT CAA TAT TGG CGT GGT TCT ATC GAA TAT AGA TTT GAT ATA GTA GCT 
                                                            =                                  


[701..720]
                S   Q   F   H   S   G   K   L   L   L   A   Y   I   P   G   L   E   E   G   G  
MT753155.1     TCA CAA TTT CAC AGT GGA AAA TTA TTG TTA GCT TAT ATT CCA GGA TTG GAA GAG GGT GGG 
                S   Q   F   H   S   G   K   L   L   L   A   Y   I   P   G   L   E   E   G   G  
MN938851.1     TCA CAA TTT CAC AGT GGA AAA TTA TTG TTA GCT TAT ATT CCA GGA TTG GAA GAG GGT GGG 
                S   Q   F   H   S   G   K   L   L   L   A   Y   I   P   G   L   E   E   G   G  
LdIV1_JGS      TCA CAA TTT CAC AGT GGA AAA TTA TTG TTA GCA TAC ATT CCA GGG CTG GAA GAA GGT GGT 
                S   Q   F   H   S   G   K   L   L   L   A   Y   I   P   G   L   E   E   G   G  
LdIV1_ZY       TCA CAA TTT CAC AGT GGA AAA TTA TTG TTA GCA TAC ATT CCA GGG CTG GAA GAA GGT GGT 
                S   Q   F   H   S   G   K   L   L   L   A   Y   I   P   G   L   E   E   G   G  
LdIV1_NJ       TCA CAA TTT CAC AGT GGA AAA TTA TTG TTA GCA TAC ATT CCA GGG CTG GAA GAA GGT GGT 
                S   Q   F   H   S   G   K   L   L   L   A   Y   I   P   G   L   E   E   G   G  
LdIV1_CT       TCA CAA TTT CAC AGT GGA AAA TTA TTG TTA GCA TAC ATT CCA GGG CTG GAA GAA GGT GGT 
                S   Q   F   H   S   G   K   L   L   L   A   Y   I   P   G   L   E   E   G   G  
KJ629170.1     TCA CAA TTT CAC AGT GGA AAA TTA TTG TTA GCA TAC ATT CCA GGG CTG GAA GAA GGT GGT 
                                                        =   =           =   =       =       =  


[721..740]
                S   V   T   I   E   Q   A   R   A   S   P   H   I   I   I   S   L   D   N   A  
MT753155.1     TCT GTC ACA ATT GAA CAA GCA CGT GCC TCA CCT CAT ATA ATA ATT TCT TTA GAT AAC GCT 
                S   V   T   I   E   Q   A   R   A   S   P   H   I   I   I   S   L   D   N   A  
MN938851.1     TCT GTC ACA ATT GAA CAA GCA CGT GCT TCA CCT CAT ATA ATA ATT TCT TTA GAT AAC GCT 
                S   V   T   I   E   Q   A   R   A   S   P   H   I   I   I   S   L   D   N   A  
LdIV1_JGS      TCT GTT ACG ATT GAA CAA GCG CGT GCT TCA CCT CAT ATA ATA ATT TCT TTA GAT AAT GCT 
                S   V   T   I   E   Q   A   R   A   S   P   H   I   I   I   S   L   D   N   A  
LdIV1_ZY       TCT GTT ACG ATT GAA CAA GCG CGT GCT TCA CCT CAT ATA ATA ATT TCT TTA GAT AAT GCT 
                S   V   T   I   E   Q   A   R   A   S   P   H   I   I   I   S   L   D   N   A  
LdIV1_NJ       TCT GTT ACG ATT GAA CAA GCG CGT GCT TCA CCT CAT ATA ATA ATT TCT TTA GAT AAT GCT 
                S   V   T   I   E   Q   A   R   A   S   P   H   I   I   I   S   L   D   N   A  
LdIV1_CT       TCT GTT ACG ATT GAA CAA GCG CGT GCT TCA CCT CAT ATA ATA ATT TCT TTA GAT AAT GCT 
                S   V   T   I   E   Q   A   R   A   S   P   H   I   I   I   S   L   D   N   A  
KJ629170.1     TCT GTT ACG ATT GAA CAA GCG CGT GCT TCA CCT CAT ATA ATA ATT TCT TTA GAT AAT GCT 
                    =   =               =       =                                       =      


[741..760]
                M   T   Y   T   W   R   V   P   Y   V   A   D   R   P   W   W   P   R   R   Y  
MT753155.1     ATG ACA TAC ACT TGG CGT GTT CCC TAT GTT GCA GAT AGA CCA TGG TGG CCT CGA CGA TAT 
                M   T   Y   T   W   R   V   P   Y   V   A   D   R   P   W   W   P   R   R   Y  
MN938851.1     ATG ACA TAC ACT TGG CGT GTT CCC TAT GTT GCA GAT AGA CCA TGG TGG CCT CGA CGA TAT 
                M   T   Y   T   W   R   V   P   Y   V   A   D   R   P   W   W   P   R   R   Y  
LdIV1_JGS      ATG ACA TAT ACT TGG CGT GTT CCT TAT GTT GCA GAT AGA CCA TGG TGG CCT CGA CGA TAT 
                M   T   Y   T   W   R   V   P   Y   V   A   D   R   P   W   W   P   R   R   Y  
LdIV1_ZY       ATG ACA TAT ACT TGG CGT GTT CCT TAT GTT GCA GAT AGA CCA TGG TGG CCT CGA CGA TAT 
                M   T   Y   T   W   R   V   P   Y   V   A   D   R   P   W   W   P   R   R   Y  
LdIV1_NJ       ATG ACA TAT ACT TGG CGT GTT CCT TAT GTT GCA GAT AGA CCA TGG TGG CCT CGA CGA TAT 
                M   T   Y   T   W   R   V   P   Y   V   A   D   R   P   W   W   P   R   R   Y  
LdIV1_CT       ATG ACA TAT ACT TGG CGT GTT CCT TAT GTT GCA GAT AGA CCA TGG TGG CCT CGA CGA TAT 
                M   T   Y   T   W   R   V   P   Y   V   A   D   R   P   W   W   P   R   R   Y  
KJ629170.1     ATG ACA TAT ACT TGG CGT GTT CCT TAT GTT GCA GAT AGA CCA TGG TGG CCT CGA CGA TAT 
                        =                   =                                                  


[761..780]
                A   G   E   S   I   S   N   N   V   T   S   P   S   K   I   F   V   F   V   L  
MT753155.1     GCA GGC GAG TCC ATA TCT AAT AAT GTG ACG TCT CCA TCA AAA ATT TTT GTA TTC GTA TTA 
                A   G   E   S   I   S   N   N   V   T   S   P   S   K   I   F   V   F   V   L  
MN938851.1     GCA GGC GAG TCC ATA TCT AAT AAT GTG ACG TCT CCA TCA AAA ATT TTT GTA TTC GTA TTA 
                A   G   E   S   I   S   N   N   V   T   S   P   S   K   I   F   V   F   V   L  
LdIV1_JGS      GCT GGC GAG TCT ATA TCT AAT AAT GTA ACG TCT CCA TCA AAA ATT TTT GTA TTT GTA TTA 
                A   G   E   S   I   S   N   N   V   T   S   P   S   K   I   F   V   F   V   L  
LdIV1_ZY       GCT GGC GAG TCT ATA TCT AAT AAT GTA ACG TCT CCA TCA AAA ATT TTT GTA TTT GTA TTA 
                A   G   E   S   I   S   N   N   V   T   S   P   S   K   I   F   V   F   V   L  
LdIV1_NJ       GCT GGC GAG TCT ATA TCT AAT AAT GTA ACG TCT CCA TCA AAA ATT TTT GTA TTT GTA TTA 
                A   G   E   S   I   S   N   N   V   T   S   P   S   K   I   F   V   F   V   L  
LdIV1_CT       GCT GGC GAG TCT ATA TCT AAT AAT GTA ACG TCT CCA TCA AAA ATT TTT GTA TTT GTA TTA 
                A   G   E   S   I   S   N   N   V   T   S   P   S   K   I   F   V   F   V   L  
KJ629170.1     GCT GGC GAG TCT ATA TCT AAT AAT GTA ACG TCT CCA TCA AAA ATT TTT GTA TTT GTA TTA 
                =           =                   =                                   =          


[781..800]
                N   E   L   V   M   A   E   T   V   A   D   S   I   E   I   L   V   Y   M   R  
MT753155.1     AAT GAG CTT GTT ATG GCA GAA ACT GTC GCT GAT AGT ATT GAA ATA TTA GTA TAT ATG CGT 
                N   E   L   V   M   A   E   T   V   A   D   S   I   E   I   L   V   Y   M   R  
MN938851.1     AAT GAG CTT GTT ATG GCA GAA ACT GTC GCT GAT AGT ATT GAA ATA TTA GTA TAT ATG CGT 
                N   E   L   V   M   A   E   T   V   A   D   S   I   E   I   L   V   Y   M   R  
LdIV1_JGS      AAT GAG CTT GTC ATG GCA GAA ACT GTC GCA GAT AGT ATC GAA ATT TTG GTA TAT ATG CGT 
                N   E   L   V   M   A   E   T   V   A   D   S   I   E   I   L   V   Y   M   R  
LdIV1_ZY       AAT GAG CTT GTC ATG GCA GAA ACT GTC GCA GAT AGT ATC GAA ATT TTG GTA TAT ATG CGT 
                N   E   L   V   M   A   E   T   V   A   D   S   I   E   I   L   V   Y   M   R  
LdIV1_NJ       AAT GAG CTT GTC ATG GCA GAA ACT GTT GCA GAT AGT ATC GAA ATT TTG GTA TAT ATG CGT 
                N   E   L   V   M   A   E   T   V   A   D   S   I   E   I   L   V   Y   M   R  
LdIV1_CT       AAT GAG CTT GTC ATG GCA GAA ACT GTT GCA GAT AGT ATC GAA ATT TTG GTA TAT ATG CGT 
                N   E   L   V   M   A   E   T   V   A   D   S   I   E   I   L   V   Y   M   R  
KJ629170.1     AAT GAG CTT GTC ATG GCA GAA ACT GTT GCA GAT AGT ATC GAA ATT TTG GTA TAT ATG CGT 
                            =                   =   =           =       =   =                  


[801..820]
                G   G   E   D   M   E   F   A   V   P   V   Q   P   S   I   G   L   G   Y   D  
MT753155.1     GGT GGC GAA GAT ATG GAA TTT GCT GTG CCT GTT CAA CCT TCA ATA GGT TTG GGT TAC GAT 
                G   G   E   D   M   E   F   A   V   P   V   Q   P   S   I   G   L   G   Y   D  
MN938851.1     GGT GGT GAA GAT ATG GAA TTT GCT GTG CCT GTT CAA CCT TCA ATA GGT TTG GGT TAC GAT 
                G   G   E   D   M   E   F   A   V   P   V   Q   P   S   I   G   L   G   Y   D  
LdIV1_JGS      GGT GGT GAA GAT ATG GAA TTT GCT GTG CCT GTT CAA CCT TCA ATA GGT TTG GGT TAT GAT 
                G   G   E   D   M   E   F   A   V   P   V   Q   P   S   I   G   L   G   Y   D  
LdIV1_ZY       GGT GGT GAA GAT ATG GAA TTT GCT GTG CCT GTT CAA CCT TCA ATA GGT TTG GGT TAT GAT 
                G   G   E   D   M   E   F   A   V   P   V   Q   P   S   I   G   L   G   Y   D  
LdIV1_NJ       GGT GGT GAA GAT ATG GAA TTT GCT GTG CCT GTT CAA CCT TCA ATA GGT TTG GGT TAT GAT 
                G   G   E   D   M   E   F   A   V   P   V   Q   P   S   I   G   L   G   Y   D  
LdIV1_CT       GGT GGT GAA GAT ATG GAA TTT GCT GTG CCT GTT CAA CCT TCA ATA GGT TTG GGT TAT GAT 
                G   G   E   D   M   E   F   A   V   P   V   Q   P   S   I   G   L   G   H   D  
KJ629170.1     GGT GGT GAA GAC ATG GAA TTT GCT GTG CCT GTT CAA CCT TCA ATA GGT TTG GGT CAT GAT 
                    =       =                                                           ^      


[821..840]
                N   S   Y   I   A   S   R   N   N   T   N   V   F   P   V   S   T   T   D   N  
MT753155.1     AAT AGT TAT ATA GCA AGT CGA AAT AAT ACT AAT GTA TTT CCT GTA TCT ACG ACT GAT AAT 
                N   S   Y   I   A   S   R   N   N   T   N   V   F   P   V   S   T   T   D   N  
MN938851.1     AAT AGT TAT ATA GCA AGT CGA AAT AAT ACT AAT GTA TTT CCT GTA TCT ACG ACT GAT AAT 
                N   S   Y   I   A   S   R   N   N   T   N   V   F   P   V   S   T   T   D   N  
LdIV1_JGS      AAT AGT TAT ATA GCA AGT CGA AAT AAT ACT AAT GTA TTT CCT GTA TCC ACA ACT GAT AAT 
                N   S   Y   I   A   S   R   N   N   T   N   V   F   P   V   S   T   T   D   N  
LdIV1_ZY       AAT AGT TAT ATA GCA AGT CGA AAT AAT ACT AAT GTA TTT CCT GTA TCC ACA ACT GAT AAT 
                N   S   Y   I   A   S   R   N   N   T   N   V   F   P   V   S   T   T   D   N  
LdIV1_NJ       AAT AGT TAT ATA GCA AGT CGA AAT AAT ACT AAT GTA TTT CCT GTA TCC ACA ACT GAT AAT 
                N   S   Y   I   A   S   R   N   N   T   N   V   F   P   V   S   T   T   D   N  
LdIV1_CT       AAT AGT TAT ATA GCA AGT CGA AAT AAT ACT AAT GTA TTT CCT GTA TCC ACA ACT GAT AAT 
                N   S   Y   V   A   S   R   N   N   T   N   I   F   P   V   S   T   T   D   S  
KJ629170.1     AAT AGT TAT GTA GCA AGT CGA AAT AAT ACT AAT ATA TTT CCT GTA TCC ACA ACT GAT AGT 
                            ^                               ^               =   =           ^  


[841..860]
                F   Y   V   G   N   W   H   T   T   P   L   V   Q   V   L   R   H   A   T   T  
MT753155.1     TTT TAT GTT GGT AAT TGG CAT ACC ACA CCA TTG GTT CAA GTT TTG CGT CAT GCT ACT ACA 
                F   Y   V   G   N   W   H   T   T   P   L   V   Q   V   L   R   H   A   T   T  
MN938851.1     TTT TAT GTT GGT AAT TGG CAT ACC ACA CCA TTG GTT CAA GTT TTG CGT CAT GCT ACT ACA 
                F   Y   V   G   N   W   H   S   T   P   L   V   Q   V   L   R   H   A   T   T  
LdIV1_JGS      TTT TAT GTT GGA AAT TGG CAT TCT ACA CCA TTA GTT CAG GTT TTG CGT CAC GCT ACT ACA 
                F   Y   V   G   N   W   H   S   T   P   L   V   Q   V   L   R   H   A   T   T  
LdIV1_ZY       TTT TAT GTT GGA AAT TGG CAT TCT ACA CCA TTA GTT CAG GTT TTG CGT CAC GCT ACT ACA 
                F   Y   V   G   N   W   H   S   T   P   L   V   Q   V   L   R   H   A   T   T  
LdIV1_NJ       TTT TAT GTT GGA AAT TGG CAT TCT ACA CCA TTA GTT CAG GTT TTG CGT CAC GCT ACT ACA 
                F   Y   V   G   N   W   H   S   T   P   L   V   Q   V   L   R   H   A   T   T  
LdIV1_CT       TTT TAT GTT GGA AAT TGG CAT TCT ACA CCA TTA GTT CAG GTT TTG CGT CAC GCT ACT ACA 
                F   Y   A   G   N   W   H   S   A   P   L   V   L   V   L   R   H   A   A   T  
KJ629170.1     TTC TAT GCT GGA AAT TGG CAT TCT GCA CCA TTA GTT CTG GTT TTG CGT CAC GCT GCT ACA 
                =       ^   =               ^   ^       =       ^               =       ^      


[861..880]
                S   E   A   V   G   R   F   S   E   P   I   L   D   R   P   V   Y   Y   V   L  
MT753155.1     TCA GAA GCT GTA GGA CGA TTT AGT GAG CCT ATA TTA GAC CGT CCC GTT TAT TAT GTT CTA 
                S   E   A   V   G   R   F   S   E   P   I   L   D   R   P   V   Y   Y   V   L  
MN938851.1     TCA GAA GCT GTA GGA CGA TTT AGT GAG CCT ATA TTA GAC CGT CCC GTT TAT TAT GTT CTA 
                S   E   A   V   G   R   F   S   E   P   I   L   D   R   P   V   Y   Y   V   L  
LdIV1_JGS      TCA GAA GCT GTT GGG CGA TTT AGT GAG CCT ATA TTA GAT CGT CCT GTT TAT TAT GTT TTG 
                S   E   A   V   G   R   F   S   E   P   I   L   D   R   P   V   Y   Y   V   L  
LdIV1_ZY       TCA GAA GCT GTT GGG CGA TTT AGT GAG CCT ATA TTA GAT CGT CCT GTT TAT TAT GTT TTG 
                S   E   A   V   G   R   F   S   E   P   I   L   D   R   P   V   Y   Y   V   L  
LdIV1_NJ       TCA GAA GCT GTT GGG CGA TTT AGT GAG CCT ATA TTA GAT CGT CCT GTT TAT TAT GTT TTG 
                S   E   A   V   G   R   F   S   E   P   I   L   D   R   P   V   Y   Y   V   L  
LdIV1_CT       TCA GAA GCT GTT GGG CGA TTT AGT GAG CCT ATA TTA GAT CGT CCT GTT TAT TAT GTT TTG 
                S   D   A   V   G   R   F   S   E   P   I   L   D   R   P   V   Y   Y   V   L  
KJ629170.1     TCA GAC GCT GTT GGG CGA TTT AGT GAG CCT ATA TTA GAT CGT CCT GTT TAT TAT GTT TTG 
                    ^       =   =                               =       =                   =  


[881..900]
                S   S   S   L   P   T   A   N   V   N   S   A   A   I   T   L   R   S   I   T  
MT753155.1     TCC TCG TCG CTT CCT ACG GCA AAC GTT AAT TCT GCA GCT ATA ACT TTA CGA TCG ATT ACA 
                S   S   S   L   P   T   A   N   V   N   S   A   A   I   T   L   R   S   I   T  
MN938851.1     TCC TCG TCG CTT CCT ACG GCA AAC GTT AAT TCT GCA GCT ATA ACT TTA CGA TCG ATT ACA 
                S   S   S   L   P   T   A   N   V   N   S   A   A   I   T   L   R   S   I   T  
LdIV1_JGS      TCT TCG TCG CTT CCT ACG GCA AAT GTT AAT TCT GCA GCT ATA ACT TTA CGA TCA ATT ACA 
                S   S   S   L   P   T   A   N   V   N   S   A   A   I   T   L   R   S   I   T  
LdIV1_ZY       TCT TCG TCG CTT CCT ACG GCA AAT GTT AAT TCT GCA GCT ATA ACT TTA CGA TCA ATT ACA 
                S   S   S   L   P   T   A   N   V   N   S   A   A   I   T   L   R   S   I   T  
LdIV1_NJ       TCT TCG TCG CTT CCT ACG GCA AAT GTT AAT TCT GCA GCT ATA ACT TTA CGA TCA ATT ACA 
                S   S   S   L   P   T   A   N   V   N   S   A   A   I   T   L   R   S   I   T  
LdIV1_CT       TCT TCG TCG CTT CCT ACG GCA AAT GTT AAT TCT GCA GCT ATA ACT TTA CGA TCA ATT ACA 
                S   S   S   L   P   T   A   N   V   N   S   G   A   L   T   L   R   S   I   T  
KJ629170.1     TCT TCG TCG CTT CCT ACG GCA AAT GTT AAT TCT GGA GCT TTA ACT TTA CGA TCA ATT ACA 
                =                           =               ^       ^               =          


[901..920]
                H   Y   I   F   L   K   G   I   G   F   S   E   Y   I   G   L   P   V   Y   A  
MT753155.1     CAC TAT ATT TTC TTG AAA GGT ATT GGT TTT AGC GAA TAT ATA GGT TTG CCT GTT TAT GCA 
                H   Y   I   F   L   K   G   I   G   F   S   E   Y   I   G   L   P   V   Y   A  
MN938851.1     CAC TAT ATT TTC TTG AAA GGT ATT GGT TTT AGC GAA TAT ATA GGT TTG CCT GTT TAT GCA 
                H   Y   I   F   L   K   G   I   G   F   N   E   Y   I   G   L   P   V   Y   A  
LdIV1_JGS      CAT TAC ATT TTC TTA AAA GGT ATT GGT TTT AAC GAA TAT ATA GGT TTA CCT GTT TAT GCA 
                H   Y   I   F   L   K   G   I   G   F   N   E   Y   I   G   L   P   V   Y   A  
LdIV1_ZY       CAT TAC ATT TTC TTA AAA GGT ATT GGT TTT AAC GAA TAT ATA GGT TTA CCT GTT TAT GCA 
                H   Y   I   F   L   K   G   I   G   F   N   E   Y   I   G   L   P   V   Y   A  
LdIV1_NJ       CAT TAC ATT TTC TTA AAA GGT ATT GGT TTT AAC GAA TAT ATA GGT TTA CCT GTT TAT GCA 
                H   Y   I   F   L   K   G   I   G   F   N   E   Y   I   G   L   P   V   Y   A  
LdIV1_CT       CAT TAC ATT TTC TTA AAA GGT ATT GGT TTT AAT GAA TAT ATA GGT TTA CCT GTT TAT GCA 
                H   Y   I   F   L   K   G   I   G   F   N   E   Y   I   G   L   P   V   Y   A  
KJ629170.1     CAT TAC ATT TTC TTA AAA GGT ATT GGT TTT AAC GAA TAT ATA GGT TTA CCT GTT TAT GCA 
                =   =           =                       ^                   =                  


[921..940]
                V   D   T   Q   I   A   L   S   R   L   E   Q   I   A   R   A   A   F   T   N  
MT753155.1     GTT GAT ACT CAA ATT GCT CTT AGT AGG CTT GAA CAG ATA GCG CGT GCT GCA TTT ACT AAT 
                V   D   T   Q   I   A   L   S   R   L   E   Q   I   A   R   A   A   F   T   N  
MN938851.1     GTT GAT ACT CAA ATT GCT CTT AGT AGG CTT GAA CAG ATA GCG CGT GCT GCA TTT ACT AAT 
                V   D   S   Q   I   A   L   S   R   L   E   Q   I   A   R   A   A   F   T   N  
LdIV1_JGS      GTT GAT TCT CAA ATT GCT CTT AGT AGG CTT GAA CAG ATA GCG CGT GCT GCA TTT ACT AAT 
                V   D   S   Q   I   A   L   S   R   L   E   Q   I   A   R   A   A   F   T   N  
LdIV1_ZY       GTT GAT TCT CAA ATT GCT CTT AGT AGG CTT GAA CAG ATA GCG CGT GCT GCA TTT ACT AAT 
                V   D   T   Q   I   A   L   S   R   L   E   Q   I   A   R   A   A   F   T   N  
LdIV1_NJ       GTT GAT ACT CAA ATT GCT CTT AGT AGG CTT GAA CAG ATA GCG CGT GCC GCA TTT ACT AAT 
                V   D   T   Q   I   A   L   S   R   L   E   Q   I   A   R   A   A   F   T   N  
LdIV1_CT       GTT GAT ACT CAA ATT GCT CTT AGT AGG CTT GAA CAG ATA GCG CGT GCC GCA TTT ACT AAT 
                A   N   T   Q   I   A   L   N   K   L   E   Q   I   A   R   A   A   F   T   N  
KJ629170.1     GCT AAT ACT CAA ATT GCT CTT AAT AAG CTT GAA CAG ATA GCG CGT GCC GCA TTT ACT AAT 
                ^   ^   ^                   ^   ^                           =                  


[941..960]
                N   Y   T   Y   G   S   W   V   S   S   F   I   I   T   A   P   L   S   T   S  
MT753155.1     AAT TAT ACA TAT GGT TCT TGG GTA AGT TCA TTT ATA ATA ACT GCA CCA CTT TCA ACA TCG 
                N   Y   T   Y   G   S   W   V   S   S   F   I   I   T   A   P   L   S   T   S  
MN938851.1     AAT TAT ACA TAT GGT TCT TGG GTA AGT TCA TTT ATA ATA ACT GCA CCA CTT TCA ACA TCG 
                N   Y   T   Y   G   S   W   V   S   S   F   M   I   T   A   P   L   S   T   S  
LdIV1_JGS      AAT TAT ACA TAT GGT TCT TGG GTA AGT TCA TTT ATG ATA ACT GCA CCA CTT TCG ACA TCG 
                N   Y   T   Y   G   S   W   V   S   S   F   M   I   T   A   P   L   S   T   S  
LdIV1_ZY       AAT TAT ACA TAT GGT TCT TGG GTA AGT TCA TTT ATG ATA ACT GCA CCA CTT TCG ACA TCG 
                N   Y   T   Y   G   S   W   V   S   S   F   M   I   T   A   P   L   S   T   S  
LdIV1_NJ       AAT TAC ACA TAT GGT TCT TGG GTA AGT TCA TTT ATG ATA ACT GCA CCA CTT TCA ACA TCA 
                N   Y   T   Y   G   S   W   V   S   S   F   M   I   T   A   P   L   S   T   S  
LdIV1_CT       AAT TAC ACA TAT GGT TCT TGG GTA AGT TCA TTT ATG ATA ACT GCA CCA CTT TCA ACA TCA 
                K   Y   T   Y   G   S   W   V   S   S   F   M   I   T   A   P   L   S   T   S  
KJ629170.1     AAG TAC ACA TAT GGT TCT TGG GTA AGT TCA TTT ATG ATA ACT GCA CCA CTT TCA ACA TCA 
                ^   =                                       ^                       =       =  


[961..980]
                A   G   G   F   G   F   I   P   A   A   Y   V   T   T   S   N   T   Y   G   G  
MT753155.1     GCA GGC GGT TTT GGT TTC ATA CCT GCA GCA TAC GTT ACA ACA TCT AAT ACG TAT GGA GGA 
                A   G   G   F   G   F   I   P   A   A   Y   V   T   T   S   N   T   Y   G   G  
MN938851.1     GCA GGC GGT TTC GGT TTC ATA CCT GCA GCA TAC GTT ACA ACA TCT AAT ACG TAT GGA GGA 
                A   G   G   F   G   F   I   P   A   A   Y   V   T   T   S   N   T   Y   G   G  
LdIV1_JGS      GCA GGT GGT TTT GGT TTT ATA CCT GCA GCA TAC GTT ACA ACA TCT AAT ACG TAT GGA GGA 
                A   G   G   F   G   F   I   P   A   A   Y   V   T   T   S   N   T   Y   G   G  
LdIV1_ZY       GCA GGT GGT TTT GGT TTT ATA CCT GCA GCA TAC GTT ACA ACA TCT AAT ACG TAT GGA GGA 
                A   G   G   F   G   F   I   P   A   A   Y   V   T   T   S   N   T   Y   G   G  
LdIV1_NJ       GCA GGC GGC TTC GGT TTC ATA CCC GCA GCA TAT GTT ACA ACG TCT AAT ACG TAT GGA GGA 
                A   G   G   F   G   F   I   P   A   A   Y   V   T   T   S   N   T   Y   G   G  
LdIV1_CT       GCA GGC GGC TTC GGT TTC ATA CCC GCA GCA TAT GTT ACA ACG TCT AAT ACG TAT GGA GGA 
                A   G   G   F   G   F   I   P   A   A   Y   V   T   T   S   N   T   Y   G   G  
KJ629170.1     GCA GGC GGC TTC GGT TTC ATA CCC GCA GCA TAT GTT ACA ACG TCT AAT ACG TAT GGA GGA 
                    =   =   =       =       =           =           =                          


[981..1000]
                G   K   S   I   P   F   V   A   T   S   V   S   P   T   L   E   D   M   E   Y  
MT753155.1     GGA AAA TCA ATT CCC TTC GTA GCT ACG AGT GTT TCG CCT ACA TTA GAA GAT ATG GAA TAT 
                G   K   S   I   P   F   V   A   T   S   V   S   P   T   L   E   D   M   E   Y  
MN938851.1     GGA AAA TCA ATT CCC TTC GTA GCT ACG AGT GTT TCG CCT ACA TTA GAA GAT ATG GAA TAT 
                G   K   S   I   P   F   I   A   T   S   V   S   P   T   L   E   D   M   E   Y  
LdIV1_JGS      GGG AAA TCA ATT CCC TTT ATA GCT ACG AGT GTT TCG CCT ACT TTA GAA GAT ATG GAA TAT 
                G   K   S   I   P   F   I   A   T   S   V   S   P   T   L   E   D   M   E   Y  
LdIV1_ZY       GGG AAA TCA ATT CCC TTT ATA GCT ACG AGT GTT TCG CCT ACT TTA GAA GAT ATG GAA TAT 
                G   K   S   I   P   F   T   A   T   S   V   S   P   T   L   E   D   M   E   Y  
LdIV1_NJ       GGG AAA TCA ATT CCC TTT ACA GCT ACG AGT GTG TCA CCT ACC TTA GAA GAT ATG GAG TAT 
                G   K   S   I   P   F   T   A   T   S   V   S   P   T   L   E   D   M   E   Y  
LdIV1_CT       GGG AAA TCA ATT CCC TTT ACA GCT ACG AGT GTG TCA CCT ACC TTA GAA GAT ATG GAG TAT 
                G   K   S   I   P   F   X   A   M   S   V   S   P   T   L   E   D   M   E   Y  
KJ629170.1     GGG AAA TCA ATT CCC TTT AYA GCT ATG AGT GTG TCA CCT ACC TTA GAA GAT ATG GAG TAT 
                =                   =   ^       ^       =   =       =                   =      


[1001..1020]
                Q   G   N   R   E   E   S   L   A   L   V   D   D   T   Q   N   L   Q   S   T  
MT753155.1     CAG GGA AAT AGA GAG GAA TCA TTA GCA TTA GTA GAT GAT ACG CAG AAT TTG CAA AGT ACT 
                Q   G   N   R   E   E   S   L   A   L   V   D   D   T   Q   N   L   Q   S   T  
MN938851.1     CAG GGA AAT AGA GAG GAA TCA TTA GCA TTA GTA GAT GAT ACG CAG AAT TTG CAA AGT ACT 
                Q   G   N   R   E   E   S   L   A   L   V   D   D   T   Q   N   L   Q   S   T  
LdIV1_JGS      CAA GGG AAT AGA GAA GAA TCA TTA GCA TTA GTA GAT GAT ACA CAA AAT TTG CAA AGT ACT 
                Q   G   N   R   E   E   S   L   A   L   V   D   D   T   Q   N   L   Q   S   T  
LdIV1_ZY       CAA GGG AAT AGA GAA GAA TCA TTA GCA TTA GTA GAT GAT ACA CAA AAT TTG CAA AGT ACT 
                Q   G   N   R   E   E   S   L   A   L   V   D   D   T   Q   N   L   Q   S   T  
LdIV1_NJ       CAA GGA AAC AGA GAG GAA TCA CTA GCA TTA GTA GAT GAT ACA CAA AAT TTG CAA AGT ACA 
                Q   G   N   R   E   E   S   L   A   L   V   D   D   T   Q   N   L   Q   S   T  
LdIV1_CT       CAA GGA AAC AGA GAG GAA TCA CTA GCA TTA GTA GAT GAT ACA CAA AAT TTG CAA AGT ACA 
                Q   G   N   R   E   E   S   L   A   L   V   D   D   T   Q   N   L   Q   S   T  
KJ629170.1     CAA GGA AAC AGA GAG GAA TCA CTA GCA TTA GTA GAT GAT ACA CAA AAT TTG CAA AGT ACA 
                =   =   =       =           =                       =   =                   =  


[1021..1040]
                G   S   G   M   M   T   Y   G   E   R   F   V   D   L   K   D   L   G   R   R  
MT753155.1     GGC AGT GGT ATG ATG ACT TAT GGT GAG CGA TTT GTG GAT TTG AAA GAT TTA GGT AGG CGT 
                G   S   G   M   M   T   Y   G   E   R   F   V   D   L   K   D   L   G   R   R  
MN938851.1     GGC AGT GGT ATG ATG ACT TAT GGT GAG CGA TTT GTG GAT TTG AAA GAT TTA GGT AGG CGT 
                G   S   G   M   M   T   Y   G   E   R   F   V   D   L   K   D   L   G   R   R  
LdIV1_JGS      GGC AGT GGC ATG ATG ACT TAT GGT GAG CGA TTT GTG GAT TTG AAA GAT TTA GGT AGG CGT 
                G   S   G   M   M   T   Y   G   E   R   F   V   D   L   K   D   L   G   R   R  
LdIV1_ZY       GGC AGT GGC ATG ATG ACT TAT GGT GAG CGA TTT GTG GAT TTG AAA GAT TTA GGT AGG CGT 
                G   S   G   M   M   T   Y   G   E   R   F   V   D   L   K   D   L   G   R   R  
LdIV1_NJ       GGT AGT GGT ATG ATG ACT TAT GGT GAG CGA TTT GTG GAT TTG AAA GAT TTA GGT AGG CGT 
                G   S   G   M   M   T   Y   G   E   R   F   V   D   L   K   D   L   G   R   R  
LdIV1_CT       GGT AGT GGT ATG ATG ACT TAT GGT GAG CGA TTT GTG GAT TTG AAA GAT TTA GGT AGG CGT 
                G   S   G   M   M   T   Y   G   E   R   F   V   D   L   K   D   L   G   R   R  
KJ629170.1     GGT AGT GGC ATG ATG ACT TAT GGT GAG CGA TTT GTG GAT TTG AAA GAT TTA GGT AGG CGT 
                =       =                                                                      


[1041..1060]
                Y   Q   L   Y   G   W   T   S   I   P   S   D   Q   I   E   R   D   P   G   A  
MT753155.1     TAC CAA CTA TAT GGC TGG ACA TCT ATA CCA AGT GAT CAA ATA GAA CGA GAT CCA GGT GCA 
                Y   Q   L   Y   G   W   T   S   I   P   S   D   Q   I   E   R   D   P   G   A  
MN938851.1     TAC CAA CTA TAT GGC TGG ACA TCT ATA CCA AGT GAT CAA ATA GAA CGA GAT CCA GGT GCA 
                Y   Q   L   Y   G   W   T   S   I   P   S   D   Q   I   E   R   D   P   G   A  
LdIV1_JGS      TAC CAA CTA TAT GGA TGG ACA TCT ATA CCA AGC GAT CAA ATA GAA CGA GAT CCA GGT GCA 
                Y   Q   L   Y   G   W   T   S   I   P   S   D   Q   I   E   R   D   P   G   A  
LdIV1_ZY       TAC CAA CTA TAT GGA TGG ACA TCT ATA CCA AGC GAT CAA ATA GAA CGA GAT CCA GGT GCA 
                Y   Q   L   Y   G   W   T   S   I   P   S   D   Q   I   E   R   D   P   G   A  
LdIV1_NJ       TAC CAA CTA TAT GGC TGG ACA TCT ATA CCG AGT GAT CAA ATA GAA CGA GAT CCA GGT GCG 
                Y   Q   L   Y   G   W   T   S   I   P   S   D   Q   I   E   R   D   P   G   A  
LdIV1_CT       TAC CAA CTA TAT GGC TGG ACA TCT ATA CCG AGT GAT CAA ATA GAA CGA GAT CCA GGT GCG 
                Y   Q   L   Y   G   W   T   S   I   P   R   D   Q   I   E   R   D   P   G   A  
KJ629170.1     TAC CAA CTA TAT GGC TGG ACA TCT ATA CCG CGT GAT CAA ATA GAA CGA GAT CCA GGT GCG 
                                =                   =   ^                                   =  


[1061..1080]
                C   S   F   L   F   P   V   L   P   Q   G   L   N   L   A   I   N   T   P   T  
MT753155.1     TGT AGT TTC TTA TTC CCC GTT CTC CCT CAA GGG TTG AAT TTG GCC ATT AAT ACG CCT ACT 
                C   S   F   L   F   P   V   L   P   Q   G   L   N   L   A   I   N   T   P   T  
MN938851.1     TGT AGT TTC TTA TTC CCC GTT CTC CCT CAA GGG TTG AAT TTG GCC ATT AAT ACG CCT ACT 
                C   S   F   L   F   P   V   L   P   Q   G   L   N   L   A   I   N   T   P   T  
LdIV1_JGS      TGT AGT TTC TTA TTC CCC GTT CTC CCT CAG GGG TTG AAT TTG GCA ATT AAT ACG CCT ACT 
                C   S   F   L   F   P   V   L   P   Q   G   L   N   L   A   I   N   T   P   T  
LdIV1_ZY       TGT AGT TTC TTA TTC CCC GTT CTC CCT CAG GGG TTG AAT TTG GCA ATT AAT ACG CCT ACT 
                C   S   F   L   F   P   V   L   P   Q   G   L   N   L   A   I   N   T   P   T  
LdIV1_NJ       TGT AGT TTC TTA TTC CCC GTT CTT CCT CAG GGG TTG AAT TTG GCT ATT AAT ACG CCT ACT 
                C   S   F   L   F   P   V   L   P   Q   G   L   N   L   A   I   N   T   P   T  
LdIV1_CT       TGT AGT TTC TTA TTC CCC GTT CTT CCT CAG GGG TTG AAT TTG GCT ATT AAT ACG CCT ACT 
                C   S   F   L   F   P   V   L   P   Q   G   L   N   L   A   I   N   T   P   T  
KJ629170.1     TGT AGT TTC TTA TTC CCC GTT CTT CCT CAG GGG TTG AAT TTG GCT ATT AAT ACG CCT ACT 
                                            =       =                   =                      


[1081..1100]
                S   V   N   Q   I   W   N   R   A   R   E   G   H   I   P   L   I   A   S   L  
MT753155.1     TCC GTT AAT CAG ATT TGG AAT CGT GCT CGT GAG GGC CAT ATA CCA TTA ATA GCA AGT TTA 
                S   V   N   Q   I   W   N   R   A   R   E   G   H   I   P   L   I   A   S   L  
MN938851.1     TCC GTT AAT CAG ATT TGG AAT CGT GCT CGT GAG GGC CAT ATA CCA TTA ATA GCA AGT TTA 
                S   V   N   Q   I   W   N   R   A   R   E   G   H   I   P   L   I   A   S   L  
LdIV1_JGS      TCC GTT AAT CAG ATT TGG AAT CGT GCT CGT GAG GGC CAT ATA CCA TTA ATA GCA AGT TTA 
                S   V   N   Q   I   W   N   R   A   R   E   G   H   I   P   L   I   A   S   L  
LdIV1_ZY       TCC GTT AAT CAG ATT TGG AAT CGT GCT CGT GAG GGC CAT ATA CCA TTA ATA GCA AGT TTA 
                S   V   N   Q   I   W   N   R   A   R   E   G   H   I   P   L   I   A   S   L  
LdIV1_NJ       TCT GTT AAT CAG ATT TGG AAT CGT GCT CGT GAG GGC CAT ATA CCA TTA ATA GCA AGT TTA 
                S   V   N   Q   I   W   N   R   A   R   E   G   H   I   P   L   I   A   S   L  
LdIV1_CT       TCT GTT AAT CAG ATT TGG AAT CGT GCT CGT GAG GGC CAT ATA CCA TTA ATA GCA AGT TTA 
                S   V   N   Q   I   W   N   R   A   R   E   G   H   I   P   L   I   A   S   L  
KJ629170.1     TCT GTT AAT CAA ATT TGG AAT CGT GCT CGT GAG GGC CAT ATA CCA TTA ATA GCA AGT TTA 
                =           =                                                                  


[1101..1120]
                F   R   F   Y   R   G   S   L   R   I   R   M   V   F   S   N   A   S   G   L  
MT753155.1     TTC CGT TTT TAT AGA GGT TCA CTT CGA ATT CGT ATG GTA TTC TCT AAT GCA TCA GGT TTA 
                F   R   F   Y   R   G   S   L   R   I   R   M   V   F   S   N   A   S   G   L  
MN938851.1     TTC CGT TTT TAT AGA GGT TCA CTT CGA ATT CGT ATG GTA TTC TCT AAT GCA TCA GGT TTA 
                F   R   F   Y   R   G   S   L   R   I   R   M   V   F   S   N   A   S   G   L  
LdIV1_JGS      TTC CGT TTT TAT AGA GGT TCA CTT CGA ATT CGT ATG GTA TTC TCT AAT GCG TCA GGT TTA 
                F   R   F   Y   R   G   S   L   R   I   R   M   V   F   S   N   A   S   G   L  
LdIV1_ZY       TTC CGT TTT TAT AGA GGT TCA CTT CGA ATT CGT ATG GTA TTC TCT AAT GCG TCA GGT TTA 
                F   R   F   Y   R   G   S   L   R   I   R   M   V   F   S   N   A   S   G   L  
LdIV1_NJ       TTC CGT TTT TAC AGA GGT TCA CTT CGA ATT CGT ATG GTA TTC TCT AAT GCA TCA GGT CTA 
                F   R   F   Y   R   G   S   L   R   I   R   M   V   F   S   N   A   S   G   L  
LdIV1_CT       TTC CGT TTT TAC AGA GGT TCA CTT CGA ATT CGT ATG GTA TTC TCT AAT GCA TCA GGT CTA 
                F   R   F   Y   R   G   S   L   R   I   R   M   V   F   S   N   A   S   G   L  
KJ629170.1     TTC CGT TTT TAC AGA GGT TCA CTT CGA ATT CGC ATG GTA TTC TCT AAT GCA TCA GGT TTA 
                            =                           =                       =           =  


[1121..1140]
                T   A   W   V   Q   H   R   P   D   R   R   L   D   R   D   V   I   T   P   C  
MT753155.1     ACA GCG TGG GTT CAA CAT CGC CCC GAT CGT CGC TTA GAT CGC GAC GTT ATT ACT CCA TGT 
                T   A   W   V   Q   H   R   P   D   R   R   L   D   R   D   V   I   T   P   C  
MN938851.1     ACA GCG TGG GTT CAA CAT CGC CCC GAT CGT CGC TTA GAT CGC GAC GTC ATT ACT CCA TGT 
                T   A   W   V   Q   H   R   P   D   R   R   L   D   R   D   V   I   T   P   C  
LdIV1_JGS      ACA GCG TGG GTT CAA CAT CGC CCC GAT CGT CGC TTA GAT CGC GAC GTT ATT ACT CCA TGT 
                T   A   W   V   Q   H   R   P   D   R   R   L   D   R   D   V   I   T   P   C  
LdIV1_ZY       ACA GCG TGG GTT CAA CAT CGC CCC GAT CGT CGC TTA GAT CGC GAC GTT ATT ACT CCA TGT 
                T   A   W   V   Q   H   R   P   D   R   R   L   D   R   D   V   I   T   P   C  
LdIV1_NJ       ACA GCG TGG GTT CAA CAT CGC CCC GAT CGT CGC TTA GAT CGC GAC GTT ATT ACT CCA TGT 
                T   A   W   V   Q   H   R   P   D   R   R   L   D   R   D   V   I   T   P   C  
LdIV1_CT       ACA GCG TGG GTT CAA CAT CGC CCC GAT CGT CGC TTA GAT CGC GAC GTT ATT ACT CCA TGT 
                T   A   W   V   Q   H   R   P   D   R   R   L   D   R   D   V   I   T   P   C  
KJ629170.1     ACA GCG TGG GTT CAA CAT CGC CCC GAC CGT CGC TTA GAT CGC GAC GTT ATT ACT CCA TGT 
                                                =                           =                  


[1141..1160]
                T   Q   V   T   T   A   E   A   V   F   N   H   S   Y   G   V   Y   M   Q   S  
MT753155.1     ACT CAA GTA ACT ACA GCA GAG GCT GTT TTT AAT CAT TCA TAT GGA GTT TAT ATG CAA TCA 
                T   Q   V   T   T   A   E   A   V   F   N   H   S   Y   G   V   Y   M   Q   S  
MN938851.1     ACT CAA GTA ACT ACA GCA GAG GCT GTT TTT AAT CAT TCA TAT GGA GTT TAT ATG CAA TCA 
                T   Q   V   T   T   A   E   A   V   F   N   H   S   Y   G   V   Y   M   Q   S  
LdIV1_JGS      ACT CAG GTA ACT ACA GCT GAG GCT GTT TTT AAT CAC TCA TAT GGA GTT TAT ATG CAA TCA 
                T   Q   V   T   T   A   E   A   V   F   N   H   S   Y   G   V   Y   M   Q   S  
LdIV1_ZY       ACT CAG GTA ACT ACA GCT GAG GCT GTT TTT AAT CAC TCA TAT GGA GTT TAT ATG CAA TCA 
                T   Q   V   T   T   A   E   A   V   F   N   H   S   Y   G   V   Y   M   Q   S  
LdIV1_NJ       ACT CAA GTG ACT ACA GCA GAG GCT GTT TTT AAT CAT TCA TAT GGA GTT TAT ATG CAA TCA 
                T   Q   V   T   T   A   E   A   V   F   N   H   S   Y   G   V   Y   M   Q   S  
LdIV1_CT       ACC CAA GTG ACT ACA GCA GAG GCT GTT TTT AAT CAT TCA TAT GGA GTT TAT ATG CAA TCA 
                T   Q   V   T   T   A   E   A   V   F   N   H   S   Y   G   V   Y   M   Q   S  
KJ629170.1     ACT CAA GTG ACT ACA GCA GAG GCT GTT TTT AAT CAT TCA TAT GGA GTC TAT ATG CAA TCA 
                =   =   =           =                       =               =                  


[1161..1180]
                L   S   V   N   N   M   I   E   I   E   V   P   F   Y   Q   M   A   N   F   G  
MT753155.1     TTA AGC GTC AAT AAT ATG ATC GAG ATA GAA GTT CCG TTT TAT CAA ATG GCC AAT TTT GGT 
                L   S   V   N   N   M   I   E   I   E   V   P   F   Y   Q   M   A   N   F   G  
MN938851.1     TTA AGC GTC AAT AAT ATG ATC GAG ATA GAA GTT CCG TTT TAT CAA ATG GCC AAT TTT GGT 
                L   S   V   N   N   M   I   E   I   E   V   P   F   Y   Q   M   A   N   F   G  
LdIV1_JGS      TTA AGC GTT AAT AAT ATG ATC GAG ATA GAA GTT CCC TTT TAT CAA ATG GCT AAT TTT GGT 
                L   S   V   N   N   M   I   E   I   E   V   P   F   Y   Q   M   A   N   F   G  
LdIV1_ZY       TTA AGC GTT AAT AAT ATG ATC GAG ATA GAA GTT CCC TTT TAT CAA ATG GCT AAT TTT GGT 
                L   S   V   N   N   M   I   E   I   E   V   P   F   Y   Q   M   A   N   F   G  
LdIV1_NJ       TTA AGT GTC AAT AAT ATG ATC GAG ATA GAA GTT CCG TTT TAT CAA ATG GCC AAT TTT GGT 
                L   S   V   N   N   M   I   E   I   E   V   P   F   Y   Q   M   A   N   F   G  
LdIV1_CT       TTA AGT GTC AAT AAT ATG ATC GAG ATA GAA GTT CCG TTT TAT CAA ATG GCC AAT TTT GGT 
                L   S   V   N   N   M   I   E   I   E   V   P   F   Y   Q   M   A   N   F   G  
KJ629170.1     TTA AGT GTC AAT AAT ATG ATC GAG ATA GAA GTT CCG TTT TAT CAA ATG GCC AAT TTT GGT 
                    =   =                                   =                   =              


[1181..1200]
                L   L   Q   K   P   I   I   A   V   G   G   P   V   T   D   W   S   R   F   Y  
MT753155.1     CTA TTG CAA AAA CCT ATT ATC GCT GTG GGT GGA CCC GTA ACT GAT TGG TCA CGC TTT TAT 
                L   L   Q   K   P   I   I   A   V   G   G   P   V   T   D   W   S   R   F   Y  
MN938851.1     CTA TTG CAA AAA CCT ATT ATC GCT GTG GGT GGA CCC GTA ACT GAT TGG TCA CGC TTT TAT 
                L   L   Q   K   P   I   I   A   V   G   G   P   V   T   D   W   S   R   F   Y  
LdIV1_JGS      CTT TTG CAA AAA CCT ATT ATC GCT GTA GGT GGT CCT GTA ACT GAT TGG TCG CGC TTT TAT 
                L   L   Q   K   P   I   I   A   V   G   G   P   V   T   D   W   S   R   F   Y  
LdIV1_ZY       CTT TTG CAA AAA CCT ATT ATC GCT GTA GGT GGT CCT GTA ACT GAT TGG TCG CGC TTT TAT 
                L   L   Q   K   P   I   I   A   V   G   G   P   V   T   D   W   S   R   F   Y  
LdIV1_NJ       CTG TTA CAA AAA CCC ATT ATT GCT GTA GGT GGT CCC GTA ACT GAT TGG TCG CGC TTT TAT 
                L   L   Q   K   P   I   I   A   V   G   G   P   V   T   D   W   S   R   F   Y  
LdIV1_CT       CTG TTA CAA AAA CCC ATT ATT GCT GTA GGT GGT CCC GTA ACT GAT TGG TCG CGC TTT TAT 
                L   L   Q   K   P   I   I   A   V   G   G   P   V   T   D   W   S   R   F   Y  
KJ629170.1     CTG TTA CAA AAA CCC ATT ATT GCT GTA GGT GGT CCC GTA ACT GAT TGG TCG CGC TTT TAT 
                =   =           =       =       =       =   =                   =              


[1201..1220]
                S   L   G   E   L   S   V   G   F   F   G   D   K   P   T   E   D   I   R   C  
MT753155.1     AGC TTA GGC GAA TTG AGC GTG GGC TTC TTT GGC GAT AAA CCT ACA GAA GAT ATT AGG TGT 
                S   L   G   E   L   S   V   G   F   F   G   D   K   P   T   E   D   I   R   C  
MN938851.1     AGC TTA GGC GAA TTG AGC GTG GGC TTC TTT GGC GAT AAA CCT ACA GAA GAT ATT AGG TGT 
                S   L   G   E   L   S   V   G   F   F   G   D   K   P   A   D   D   I   R   C  
LdIV1_JGS      AGC TTA GGC GAA TTG AGC GTG GGC TTT TTT GGT GAT AAA CCT GCA GAT GAT ATT AGG TGT 
                S   L   G   E   L   S   V   G   F   F   G   D   K   P   A   D   D   I   R   C  
LdIV1_ZY       AGC TTA GGC GAA TTG AGC GTG GGC TTT TTT GGT GAT AAA CCT GCA GAT GAT ATT AGG TGT 
                S   L   G   E   L   S   V   G   F   F   G   D   K   P   T   D   D   I   R   C  
LdIV1_NJ       AGC TTA GGC GAG CTG AGC GTG GGC TTT TTT GGC GAT AAA CCT ACA GAT GAT ATT AGG TGT 
                S   L   G   E   L   S   V   G   F   F   G   D   K   P   T   D   D   I   R   C  
LdIV1_CT       AGC TTA GGC GAG TTG AGT GTG GGC TTT TTT GGC GAT AAA CCT ACA GAT GAT ATT AGG TGT 
                S   L   G   E   L   S   V   G   F   F   G   D   K   P   T   D   D   I   R   C  
KJ629170.1     AGC TTA GGC GAG CTG AGC GTG GGC TTT TTT GGC GAT AAA CCT ACA GAT GAT ATT AGG TGT 
                            =   =   =           =       =               ^   ^                  


[1221..1240]
                T   I   Y   Y   S   M   A   D   D   C   R   F   S   T   Y   Q   G   V   P   P  
MT753155.1     ACG ATA TAT TAC AGT ATG GCT GAT GAT TGC CGG TTC TCG ACA TAT CAG GGA GTT CCT CCC 
                T   I   Y   Y   S   M   A   D   D   C   R   F   S   T   Y   Q   G   V   P   P  
MN938851.1     ACG ATA TAT TAC AGT ATG GCT GAT GAT TGC CGG TTC TCG ACA TAT CAG GGA GTT CCT CCC 
                T   I   Y   Y   S   M   A   D   D   C   R   F   S   T   Y   Q   G   V   P   P  
LdIV1_JGS      ACG ATA TAT TAT AGT ATG GCT GAT GAT TGC CGG TTT TCA ACG TAC CAG GGA GTT CCT CCC 
                T   I   Y   Y   S   M   A   D   D   C   R   F   S   T   Y   Q   G   V   P   P  
LdIV1_ZY       ACG ATA TAT TAT AGT ATG GCT GAT GAT TGC CGG TTT TCA ACG TAC CAG GGA GTT CCT CCC 
                T   I   Y   Y   S   M   A   D   D   C   R   F   S   T   Y   Q   G   V   P   P  
LdIV1_NJ       ACG ATA TAT TAT AGC ATG GCT GAT GAT TGC CGA TTT TCA ACA TAT CAG GGA GTT CCT CCT 
                T   I   Y   Y   S   M   A   D   D   C   R   F   S   T   Y   Q   G   V   P   P  
LdIV1_CT       ACG ATA TAT TAT AGC ATG GCT GAT GAT TGC CGA TTT TCA ACA TAT CAG GGA GTT CCT CCT 
                T   I   Y   Y   S   M   A   D   D   C   R   F   S   T   Y   Q   G   V   P   P  
KJ629170.1     ACG ATA TAT TAT AGC ATG GCT GAT GAT TGC CGA TTT TCA ACA TAT CAG GGA GTT CCT CCT 
                            =   =                       =   =   =   =   =                   =  


[1241..1260]
                V   V   L   I   D   D   L   P   E   Y   Q   G   F   T   D   Y   F   K   A   S  
MT753155.1     GTT GTT TTA ATT GAT GAT TTA CCT GAA TAC CAA GGT TTT ACA GAT TAT TTC AAG GCG TCA 
                V   V   L   I   D   D   L   P   E   Y   Q   G   F   T   D   Y   F   K   A   S  
MN938851.1     GTT GTT TTA ATT GAT GAT TTA CCT GAA TAC CAA GGT TTT ACA GAT TAT TTC AAG GCG TCA 
                V   V   L   I   D   D   L   P   E   Y   Q   G   F   T   D   Y   F   K   A   S  
LdIV1_JGS      GTT GTT TTA ATT GAC GAT TTA CCT GAA TAC CAA GGT TTT ACA GAT TAT TTT AAA GCG TCA 
                V   V   L   I   D   D   L   P   E   Y   Q   G   F   T   D   Y   F   K   A   S  
LdIV1_ZY       GTT GTT TTA ATT GAC GAT TTA CCT GAA TAC CAA GGT TTT ACA GAT TAT TTT AAA GCG TCA 
                V   V   L   I   D   D   L   P   E   Y   Q   G   F   T   D   Y   F   K   A   S  
LdIV1_NJ       GTT GTT TTA ATT GAT GAT TTA CCT GAA TAC CAA GGT TTT ACA GAT TAT TTT AAA GCG TCA 
                V   V   L   I   D   D   L   P   E   Y   Q   G   F   T   D   Y   F   K   A   S  
LdIV1_CT       GTT GTT TTA ATT GAT GAT TTA CCT GAA TAC CAA GGT TTT ACA GAT TAT TTT AAA GCG TCA 
                V   V   L   I   D   D   L   P   E   Y   Q   G   F   T   D   Y   F   K   S   S  
KJ629170.1     GTT GTT TTA ATT GAT GAT TTA CCT GAA TAC CAA GGC TTT ACA GAT TAT TTT AAA TCG TCA 
                                =                           =                   =   =   ^      


[1261..1280]
                P   K   E   L   G   N   Q   V   A   E   G   V   A   E   T   I   H   S   Q   L  
MT753155.1     CCT AAA GAA TTA GGT AAT CAA GTA GCA GAG GGT GTT GCA GAA ACT ATA CAT TCC CAG TTA 
                P   K   E   L   G   N   Q   V   A   E   G   V   A   E   T   I   H   S   Q   L  
MN938851.1     CCT AAA GAA TTA GGT AAT CAA GTA GCA GAG GGT GTT GCA GAA ACT ATA CAT TCC CAG TTA 
                P   K   E   L   G   N   Q   V   A   E   G   V   A   E   T   I   H   S   Q   L  
LdIV1_JGS      CCC AAA GAA TTA GGT AAT CAA GTA GCA GAA GGT GTT GCA GAG ACT ATA CAT TCC CAA TTA 
                P   K   E   L   G   N   Q   V   A   E   G   V   A   E   T   I   H   S   Q   L  
LdIV1_ZY       CCC AAA GAA TTA GGT AAT CAA GTA GCA GAA GGT GTT GCA GAG ACT ATA CAT TCC CAA TTA 
                P   K   E   L   G   N   Q   V   A   E   G   V   A   E   T   I   H   S   Q   L  
LdIV1_NJ       CCT AAA GAA TTA GGT AAT CAA GTA GCA GAG GGT GTT GCA GAG ACT ATA CAT TCC CAA TTA 
                P   K   E   L   G   N   Q   V   A   E   G   V   A   E   T   I   H   S   Q   L  
LdIV1_CT       CCT AAA GAA TTA GGT AAT CAA GTA GCA GAA GGT GTT GCA GAG ACT ATA CAT TCC CAA TTA 
                P   K   E   I   G   N   Q   V   A   E   G   V   A   E   T   I   H   S   Q   L  
KJ629170.1     CCT AAA GAA ATA GGT AAT CAA GTA GCA GAG GGT GTT GCA GAG ACT ATA CAT TCC CAA TTA 
                =           ^                       =               =                   =      


[1281..1300]
                Q   P   A   V   D   N   F   V   A   S   F   K   E   R   M   G   D   V   Y   S  
MT753155.1     CAG CCA GCT GTT GAC AAT TTT GTA GCA TCA TTT AAA GAA AGA ATG GGA GAT GTA TAT TCA 
                Q   P   A   V   D   N   F   V   A   S   F   K   E   R   M   G   D   V   Y   S  
MN938851.1     CAG CCA GCT GTT GAC AAT TTT GTA GCA TCA TTT AAA GAA AGA ATG GGA GAT GTA TAT TCA 
                Q   P   A   V   D   N   F   V   A   S   F   K   E   R   M   G   D   V   Y   L  
LdIV1_JGS      CAG CCA GCT GTT GAT AAT TTT GTA GCA TCT TTT AAA GAA AGA ATG GGA GAT GTA TAT TTA 
                Q   P   A   V   D   N   F   V   A   S   F   K   E   R   M   G   D   V   Y   L  
LdIV1_ZY       CAG CCA GCT GTT GAT AAT TTT GTA GCA TCT TTT AAA GAA AGA ATG GGA GAT GTA TAT TTA 
                Q   P   A   V   D   N   F   V   A   S   F   K   E   R   M   G   D   V   Y   S  
LdIV1_NJ       CAG CCA GCT GTT GAT AAT TTT GTA GCA TCA TTT AAA GAA AGA ATG GGA GAT GTG TAT TCA 
                Q   P   A   V   D   N   F   V   A   S   F   K   E   R   M   G   D   V   Y   S  
LdIV1_CT       CAG CCA GCT GTT GAT AAT TTT GTA GCA TCA TTT AAA GAA AGA ATG GGA GAT GTG TAT TCA 
                Q   P   A   V   D   N   F   V   A   S   F   K   E   R   M   G   D   V   Y   S  
KJ629170.1     CAG CCA GCT GTT GAT AAT TTT GTA GCA TCA TTT AAA GAA AGA ATG GGA GAT GTG TAT TCA 
                                =                   =                               =       ^  


[1301..1320]
                S   V   S   K   S   I   S   D   I   E   F   S   S   K   I   S   S   I   G   S  
MT753155.1     AGT GTT TCA AAG TCA ATA AGT GAT ATT GAA TTT TCT TCA AAA ATT TCT TCT ATA GGC TCT 
                S   V   S   K   S   I   S   D   I   E   F   S   S   K   I   S   S   I   G   S  
MN938851.1     AGT GTT TCA AAG TCA ATA AGT GAT ATT GAA TTT TCT TCA AAA ATT TCT TCT ATA GGC TCT 
                S   V   S   K   S   I   S   D   I   E   F   S   S   K   I   S   S   I   G   S  
LdIV1_JGS      AGT GTT TCA AAA TCA ATA AGT GAT ATT GAA TTT TCT TCA AAA ATT TCT TCT ATA GGC TCT 
                S   V   S   K   S   I   S   D   I   E   F   S   S   K   I   S   S   I   G   S  
LdIV1_ZY       AGT GTT TCA AAA TCA ATA AGT GAT ATT GAA TTT TCT TCA AAA ATT TCT TCT ATA GGC TCT 
                S   V   S   K   S   I   S   D   I   E   F   S   S   K   I   S   S   I   G   S  
LdIV1_NJ       AGT GTT TCA AAG TCA ATA AGT GAT ATT GAA TTT TCT TCA AAA ATT TCT TCT ATA GGC TCT 
                S   V   S   K   S   I   S   D   I   E   F   S   S   K   I   S   S   I   G   S  
LdIV1_CT       AGT GTT TCA AAG TCA ATA AGT GAT ATT GAA TTT TCT TCA AAA ATT TCT TCT ATA GGC TCT 
                S   V   S   Q   S   I   S   D   I   E   F   S   S   K   I   S   A   I   G   S  
KJ629170.1     AGT GTT TCA CAG TCA ATA AGT GAT ATT GAA TTT TCT TCA AAA ATT TCT GCT ATA GGC TCT 
                            ^                                                   ^              


[1321..1340]
                Q   I   I   H   S   V   N   N   P   S   P   S   T   I   A   I   S   V   V   S  
MT753155.1     CAA ATT ATA CAT TCT GTT AAT AAC CCC TCC CCG TCA ACT ATA GCT ATT TCA GTG GTC TCC 
                Q   I   I   H   S   V   N   N   P   S   P   S   T   I   A   I   S   V   V   S  
MN938851.1     CAA ATT ATA CAT TCT GTC AAT AAC CCC TCC CCG TCA ACT ATA GCT ATT TCA GTG GTC TCC 
                Q   I   V   H   S   V   N   N   P   S   P   S   T   I   A   I   S   V   V   S  
LdIV1_JGS      CAA ATT GTA CAT TCT GTT AAT AAC CCT TCC CCG TCA ACT ATA GCT ATC TCA GTA GTT TCA 
                Q   I   V   H   S   V   N   N   P   S   P   S   T   I   A   I   S   V   V   S  
LdIV1_ZY       CAA ATT GTA CAT TCT GTT AAT AAC CCT TCC CCG TCA ACT ATA GCT ATC TCA GTA GTT TCA 
                Q   I   I   H   S   V   N   N   P   S   P   S   T   I   A   I   S   V   V   S  
LdIV1_NJ       CAA ATT ATA CAT TCT GTT AAT AAC CCC TCC CCG TCA ACT ATA GCT ATT TCA GTA GTC TCA 
                Q   I   I   H   S   V   N   N   P   S   P   S   T   I   A   I   S   V   V   S  
LdIV1_CT       CAA ATT ATA CAT TCT GTT AAT AAC CCC TCC CCG TCA ACT ATA GCT ATT TCA GTA GTC TCA 
                Q   I   I   H   S   V   N   N   P   S   P   S   T   I   A   I   S   V   V   S  
KJ629170.1     CAA ATT ATA CAT TCY GTT AAT AAC CCC TCC CCG TCA ACT ATA GCT ATT TCA GTA GTC TCA 
                        ^       =   =           =                           =       =   =   =  


[1341..1360]
                I   C   I   T   L   G   L   I   T   Y   S   T   Y   H   I   I   H   K   Y   V  
MT753155.1     ATT TGT ATT ACT TTA GGA TTA ATA ACG TAT TCT ACA TAT CAT ATC ATT CAT AAA TAT GTA 
                I   C   I   T   L   G   L   I   T   Y   S   T   Y   H   I   I   H   K   Y   V  
MN938851.1     ATT TGT ATT ACT TTA GGA TTA ATA ACG TAT TCT ACA TAT CAT ATC ATT CAT AAA TAT GTA 
                I   C   I   T   L   G   L   I   T   Y   S   T   Y   H   I   V   H   K   Y   V  
LdIV1_JGS      ATT TGT ATT ACT TTA GGA TTA ATA ACG TAT TCT ACA TAC CAT ATT GTC CAT AAA TAT GTG 
                I   C   I   T   L   G   L   I   T   Y   S   T   Y   H   I   V   H   K   Y   V  
LdIV1_ZY       ATT TGT ATT ACT TTA GGA TTA ATA ACG TAT TCT ACA TAC CAT ATT GTC CAT AAA TAT GTG 
                I   C   I   T   L   G   L   I   T   Y   S   T   Y   H   I   I   H   K   Y   V  
LdIV1_NJ       ATT TGT ATT ACT TTA GGA TTA ATA ACG TAT TCT ACA TAT CAT ATT ATT CAT AAA TAT GTG 
                I   C   I   T   L   G   L   I   T   Y   S   T   Y   H   I   I   H   K   Y   V  
LdIV1_CT       ATT TGT ATT ACT TTA GGA TTA ATA ACG TAT TCT ACA TAT CAT ATT ATT CAT AAA TAT GTG 
                I   C   I   T   L   G   L   I   T   Y   A   T   Y   H   I   V   H   K   Y   V  
KJ629170.1     ATT TGT ATT ACT TTA GGA TTA ATA ACG TAT GCT ACA TAT CAT ATT GTT CAT AAA TAT GTG 
                                                        ^       =       =   ^               =  


[1361..1380]
                I   E   I   W   T   Y   I   S   N   K   V   A   S   N   K   V   H   P   E   A  
MT753155.1     ATA GAA ATT TGG ACA TAC ATA TCA AAT AAA GTA GCT TCG AAC AAA GTG CAT CCA GAA GCA 
                I   E   I   W   T   Y   I   S   N   K   V   A   S   N   K   V   H   P   E   A  
MN938851.1     ATA GAA ATT TGG ACA TAC ATA TCA AAT AAA GTA GCT TCG AAC AAA GTG CAT CCA GAA GCA 
                I   E   I   W   T   Y   I   S   N   K   V   A   S   N   K   V   H   P   E   A  
LdIV1_JGS      ATA GAA ATT TGG ACA TAC ATA TCA AAT AAA GTA GCT TCG AAC AAA GTG CAT CCA GAA GCA 
                I   E   I   W   T   Y   I   S   N   K   V   A   S   N   K   V   H   P   E   A  
LdIV1_ZY       ATA GAA ATT TGG ACA TAC ATA TCA AAT AAA GTA GCT TCG AAC AAA GTG CAT CCA GAA GCA 
                I   E   I   W   T   Y   I   S   N   K   V   A   S   N   K   V   H   P   E   A  
LdIV1_NJ       ATA GAA ATT TGG ACA TAC ATA TCA AAT AAA GTA GCT TCG AAC AAA GTG CAT CCA GAA GCA 
                I   E   I   W   T   Y   I   S   N   K   V   A   S   N   K   V   H   P   E   A  
LdIV1_CT       ATA GAA ATT TGG ACA TAC ATA TCA AAT AAA GTA GCT TCG AAC AAA GTG CAT CCA GAA GCA 
                I   E   I   W   T   Y   I   S   N   K   V   A   S   N   K   V   H   P   E   A  
KJ629170.1     ATA GAA ATT TGG ACA TAC ATA TCA AAT AAA GTA GCT TCG AAC AAA GTG CAT CCA GAA GCA 
                                                                                               


[1381..1400]
                E   T   E   V   G   V   D   A   L   K   Y   Q   T   D   T   D   N   A   V   N  
MT753155.1     GAA ACC GAA GTC GGT GTA GAT GCG TTG AAG TAT CAG ACA GAT ACT GAT AAC GCT GTC AAC 
                E   T   E   V   G   V   D   A   L   K   Y   Q   T   D   T   D   N   A   V   N  
MN938851.1     GAA ACC GAA GTC GGT GTA GAT GCG TTG AAG TAT CAG ACA GAT ACT GAT AAC GCT GTC AAC 
                E   T   E   V   G   V   D   A   L   K   Y   Q   T   D   T   D   N   A   V   N  
LdIV1_JGS      GAA ACC GAA GTC GGT GTA GAT GCG TTA AAG TAT CAG ACT GAT ACT GAT AAC GCT GTC AAT 
                E   T   E   V   G   V   D   A   L   K   Y   Q   T   D   T   D   N   A   V   N  
LdIV1_ZY       GAA ACC GAA GTC GGT GTA GAT GCG TTA AAG TAT CAG ACT GAT ACT GAT AAC GCT GTC AAT 
                E   T   E   V   G   V   D   A   L   K   Y   Q   T   D   T   D   N   A   V   N  
LdIV1_NJ       GAA ACC GAA GTC GGT GTA GAT GCG TTA AAG TAT CAG ACG GAT ACT GAC AAC GCT GTC AAC 
                E   T   E   V   G   V   D   A   L   K   Y   Q   T   D   T   D   N   A   V   N  
LdIV1_CT       GAA ACC GAA GTC GGT GTA GAT GCG TTA AAG TAT CAG ACG GAT ACT GAC AAC GCT GTC AAC 
                E   A   E   V   G   V   E   A   L   K   Y   Q   T   D   T   D   N   A   V   N  
KJ629170.1     GAA GCC GAA GTT GGT GTA GAA GCG TTA AAG TAT CAG ACG GAT ACT GAC AAY GCT GTC AAC 
                    ^       =           ^       =               =           =   =           =  


[1401..1420]
                G   F   L   S   I   I   C   G   G   L   C   T   L   F   G   M   K   N   S   L  
MT753155.1     GGG TTT CTG TCC ATA ATC TGT GGA GGA TTA TGT ACT TTA TTT GGT ATG AAA AAT AGT TTA 
                G   F   L   S   I   I   C   G   G   L   C   T   L   F   G   M   K   N   S   L  
MN938851.1     GGG TTT CTG TCC ATA ATC TGT GGA GGA TTA TGT ACT TTA TTT GGT ATG AAA AAT AGT TTA 
                G   F   L   S   I   I   C   G   G   L   C   T   L   F   G   M   K   N   S   L  
LdIV1_JGS      GGG TTT CTG TCC ATA ATC TGT GGA GGA TTA TGT ACT TTA TTT GGT ATG AAA AAT AGT TTA 
                G   F   L   S   I   I   C   G   G   L   C   T   L   F   G   M   K   N   S   L  
LdIV1_ZY       GGG TTT CTG TCC ATA ATC TGT GGA GGA TTA TGT ACT TTA TTT GGT ATG AAA AAT AGT TTA 
                G   F   L   S   I   I   C   G   G   L   C   T   L   F   G   M   K   N   S   L  
LdIV1_NJ       GGG TTT CTG TCC ATA ATC TGT GGA GGA TTA TGT ACT TTA TTT GGT ATG AAA AAT AGT TTA 
                G   F   L   S   I   I   C   G   G   L   C   T   L   F   G   M   K   N   S   L  
LdIV1_CT       GGG TTT CTG TCC ATA ATC TGT GGA GGA TTA TGT ACT TTA TTT GGT ATG AAA AAT AGT TTA 
                G   F   L   S   I   I   C   G   G   L   C   T   L   F   G   M   K   N   S   L  
KJ629170.1     GGG TTT CTG TCC ATA ATC TGT GGA GGA TTA TGT ACT TTA TTT GGT ATG AAA AAT AGT TTA 
                                                                                               


[1421..1440]
                K   Y   K   P   V   S   D   C   L   F   K   E   I   T   N   G   M   R   M   S  
MT753155.1     AAA TAT AAA CCT GTG TCG GAC TGT TTA TTT AAG GAA ATA ACA AAT GGT ATG CGA ATG TCG 
                K   Y   K   P   V   S   D   C   L   F   K   E   I   T   N   G   M   R   M   S  
MN938851.1     AAA TAT AAA CCT GTG TCG GAC TGT TTA TTT AAG GAA ATA ACA AAT GGT ATG CGA ATG TCG 
                K   Y   K   P   V   S   D   C   L   F   K   E   I   T   N   G   M   R   M   S  
LdIV1_JGS      AAA TAT AAA CCT GTG TCA GAC TGT TTA TTT AAG GAA ATA ACA AAT GGT ATG CGA ATG TCG 
                K   Y   K   P   V   S   D   C   L   F   K   E   I   T   N   G   M   R   M   S  
LdIV1_ZY       AAA TAT AAA CCT GTG TCA GAC TGT TTA TTT AAG GAA ATA ACA AAT GGT ATG CGA ATG TCG 
                K   Y   K   P   V   S   D   C   L   F   K   E   I   T   N   G   M   R   M   S  
LdIV1_NJ       AAA TAT AAA CCT GTG TCG GAC TGT TTA TTT AAG GAA ATA ACA AAT GGT ATG CGA ATG TCG 
                K   Y   K   P   M   S   D   C   L   F   K   E   I   T   N   G   M   R   M   S  
LdIV1_CT       AAA TAT AAA CCT ATG TCG GAC TGT TTA TTT AAG GAA ATA ACA AAT GGT ATG CGA ATG TCG 
                K   Y   K   P   V   S   D   C   L   F   K   E   I   T   N   G   M   R   M   S  
KJ629170.1     AAA TAT AAA CCT GTG TCG GAC TGT TTA TTT AAG GAA ATA ACA AAT GGT ATG CGA ATG TCG 
                                ^   =                                                          


[1441..1460]
                N   V   C   F   V   F   F   K   N   L   L   S   V   I   G   D   M   K   S   L  
MT753155.1     AAT GTC TGT TTT GTA TTC TTC AAA AAT TTA TTG TCT GTG ATA GGC GAC ATG AAA TCT TTA 
                N   V   C   F   V   F   F   K   N   L   L   S   V   I   G   D   M   K   S   L  
MN938851.1     AAT GTC TGT TTT GTA TTC TTC AAA AAT TTA TTG TCT GTG ATA GGC GAC ATG AAA TCT TTA 
                N   V   C   F   V   F   F   K   N   L   L   S   V   I   G   D   M   K   S   L  
LdIV1_JGS      AAT GTT TGT TTT GTA TTC TTT AAA AAT TTA TTG TCT GTG ATA GGC GAC ATG AAA TCT TTA 
                N   V   C   F   V   F   F   K   N   L   L   S   V   I   G   D   M   K   S   L  
LdIV1_ZY       AAT GTT TGT TTT GTA TTC TTT AAA AAT TTA TTG TCT GTG ATA GGC GAC ATG AAA TCT TTA 
                N   V   C   F   V   F   F   K   N   L   L   S   V   I   G   D   M   K   S   L  
LdIV1_NJ       AAT GTT TGT TTT GTA TTC TTC AAA AAT TTA TTG TCT GTA ATA GGC GAC ATG AAA TCT TTA 
                N   V   C   F   V   F   F   K   N   L   L   S   V   I   G   D   M   K   S   L  
LdIV1_CT       AAT GTC TGT TTT GTA TTC TTC AAA AAT TTA TTG TCT GTA ATA GGC GAC ATG AAA TCT TTA 
                N   V   C   F   V   F   F   K   N   L   L   S   V   I   G   D   M   K   S   L  
KJ629170.1     AAT GTT TGT TTT GTA TTC TTC AAA AAT TTA TTG TCT GTA ATA GGC GAC ATG AAA TCT TTA 
                    =                   =                       =                              


[1461..1480]
                I   V   S   H   L   Y   P   G   F   N   A   A   E   S   L   M   E   G   K   D  
MT753155.1     ATT GTG TCT CAT TTG TAT CCT GGT TTT AAT GCT GCT GAA AGT TTG ATG GAA GGT AAG GAT 
                I   V   S   H   L   Y   P   G   F   N   A   A   E   S   L   M   E   G   K   D  
MN938851.1     ATT GTG TCT CAT TTG TAT CCT GGT TTT AAT GCT GCT GAA AGT TTG ATG GAA GGT AAG GAT 
                I   V   S   H   L   Y   P   G   F   N   A   A   E   S   L   M   E   G   K   D  
LdIV1_JGS      ATT GTG TCT CAT TTA TAT CCC GGT TTT AAT GCT GCT GAA AGT TTG ATG GAA GGT AAG GAT 
                I   V   S   H   L   Y   P   G   F   N   A   A   E   S   L   M   E   G   K   D  
LdIV1_ZY       ATT GTG TCT CAT TTA TAT CCC GGT TTT AAT GCT GCT GAA AGT TTG ATG GAA GGT AAG GAT 
                I   V   S   H   L   Y   P   G   F   N   A   A   E   S   L   M   E   G   K   D  
LdIV1_NJ       ATT GTG TCT CAT TTA TAT CCC GGT TTT AAT GCT GCT GAA AGT TTG ATG GAA GGT AAG GAT 
                I   V   S   H   L   Y   P   G   F   N   A   A   E   S   L   M   E   G   K   D  
LdIV1_CT       ATT GTG TCT CAT TTA TAT CCC GGT TTT AAT GCT GCT GAA AGT TTG ATG GAA GGT AAG GAT 
                I   V   S   H   L   Y   P   G   F   N   A   A   E   S   L   M   E   G   K   D  
KJ629170.1     ATT GTK TCT CAT TTA TAT CCT GGT TTT AAT GCT GCT GAA AGT TTG ATG GAA GGT AAG GAT 
                    =           =       =                                                      


[1481..1500]
                I   I   E   K   W   A   Q   H   S   L   N   I   L   D   P   M   V   A   Q   N  
MT753155.1     ATA ATA GAA AAG TGG GCA CAG CAT TCT CTT AAT ATA TTA GAT CCT ATG GTA GCA CAA AAT 
                I   I   E   K   W   A   Q   H   S   L   N   I   L   D   P   M   V   A   Q   N  
MN938851.1     ATA ATA GAA AAG TGG GCA CAG CAT TCT CTT AAT ATA TTA GAT CCT ATG GTA GCA CAA AAT 
                I   I   E   K   W   A   Q   H   S   L   N   I   L   D   P   M   V   A   Q   N  
LdIV1_JGS      ATA ATA GAA AAG TGG GCA CAG CAT TCT CTT AAT ATA TTA GAT CCT ATG GTA GCA CAA AAT 
                I   I   E   K   W   A   Q   H   S   L   N   I   L   D   P   M   V   A   Q   N  
LdIV1_ZY       ATA ATA GAA AAG TGG GCA CAG CAT TCT CTT AAT ATA TTA GAT CCT ATG GTA GCA CAA AAT 
                I   I   E   K   W   A   Q   H   S   L   N   I   L   D   P   M   V   A   Q   N  
LdIV1_NJ       ATA ATA GAA AAG TGG GCA CAG CAT TCT CTT AAT ATA TTA GAT CCT ATG GTA GCA CAA AAT 
                I   I   E   K   W   A   Q   H   S   L   N   I   L   D   P   M   V   A   Q   N  
LdIV1_CT       ATA ATA GAA AAG TGG GCA CAG CAT TCT CTT AAT ATA TTA GAT CCT ATG GTA GCA CAA AAT 
                I   I   E   K   W   A   Q   H   S   L   N   I   L   D   P   M   V   A   Q   N  
KJ629170.1     ATA ATA GAA AAG TGG GCT CAG CAT TCT CTC AAT ATA TTA GAT CCT ATG GTA GCA CAA AAT 
                                    =               =                                          


[1501..1520]
                I   K   Y   N   K   D   L   H   I   S   L   L   D   C   Y   A   F   G   K   I  
MT753155.1     ATT AAA TAT AAT AAA GAT TTA CAT ATT AGC TTA TTA GAT TGC TAT GCT TTT GGG AAA ATT 
                I   K   Y   N   K   D   L   H   I   S   L   L   D   C   Y   A   F   G   K   I  
MN938851.1     ATT AAA TAT AAT AAA GAT TTA CAT ATT AGC TTA TTA GAT TGC TAT GCT TTT GGG AAA ATT 
                I   K   Y   N   K   D   L   H   I   S   L   L   D   C   Y   A   F   G   K   I  
LdIV1_JGS      ATT AAA TAT AAT AAA GAT TTA CAT ATT AGC TTA TTA GAC TGC TAT GCT TTT GGG AAA ATT 
                I   K   Y   N   K   D   L   H   I   S   L   L   D   C   Y   A   F   G   K   I  
LdIV1_ZY       ATT AAA TAT AAT AAA GAT TTA CAT ATT AGC TTA TTA GAC TGC TAT GCT TTT GGG AAA ATT 
                I   K   Y   N   K   D   L   H   I   S   L   L   D   C   Y   A   F   G   K   I  
LdIV1_NJ       ATT AAA TAT AAT AAA GAT TTA CAT ATT AGC TTA TTA GAC TGC TAT GCT TTT GGG AAA ATT 
                I   K   Y   N   K   D   L   H   I   S   L   L   D   C   Y   A   F   G   K   I  
LdIV1_CT       ATT AAA TAT AAT AAA GAT TTA CAT ATT AGC TTA TTA GAC TGC TAT GCT TTT GGG AAA ATT 
                I   K   Y   D   K   D   L   H   I   S   L   L   D   C   Y   A   F   G   K   I  
KJ629170.1     ATT AAA TAT GAT AAA GAT TTA CAT ATT AGC TTA TTA GAC TGC TAT GCT TTT GGG AAA ATT 
                            ^                                   =                              


[1521..1540]
                L   K   V   K   S   L   E   T   Q   Y   P   A   L   I   Q   M   V   N   N   I  
MT753155.1     TTA AAA GTT AAA TCT TTA GAA ACG CAA TAC CCA GCT TTA ATA CAA ATG GTA AAT AAT ATA 
                L   K   V   K   S   L   E   T   Q   Y   P   A   L   I   Q   M   V   N   N   I  
MN938851.1     TTA AAA GTT AAA TCT TTA GAA ACG CAA TAC CCA GCT TTA ATA CAA ATG GTA AAT AAT ATA 
                L   K   V   K   S   L   E   T   Q   Y   P   A   L   I   Q   M   V   N   N   I  
LdIV1_JGS      TTA AAA GTT AAA TCA TTA GAA ACG CAA TAC CCA GCT TTA ATA CAA ATG GTA AAT AAT ATA 
                L   K   V   K   S   L   E   T   Q   Y   P   A   L   I   Q   M   V   N   N   I  
LdIV1_ZY       TTA AAA GTT AAA TCA TTA GAA ACG CAA TAC CCA GCT TTA ATA CAA ATG GTA AAT AAT ATA 
                L   K   V   K   S   L   E   T   Q   Y   P   A   L   I   Q   M   V   N   N   I  
LdIV1_NJ       TTA AAA GTT AAA TCA TTG GAA ACG CAA TAC CCA GCT TTA ATA CAA ATG GTA AAT AAT ATA 
                L   K   V   K   S   L   E   T   Q   Y   P   A   L   I   Q   M   V   N   N   I  
LdIV1_CT       TTA AAA GTT AAA TCA TTA GAA ACG CAA TAC CCA GCT TTA ATA CAA ATG GTA AAT AAT ATA 
                L   K   V   K   S   L   E   T   Q   Y   P   A   L   I   Q   M   V   N   N   I  
KJ629170.1     TTA AAA GTT AAA TCA TTA GAA ACG CAA TAC CCA GCT TTA ATA CAA ATG GTA AAT AAT ATA 
                                =   =                                                          


[1541..1560]
                F   D   K   L   H   R   L   Y   V   D   L   I   A   Q   G   I   D   P   H   V  
MT753155.1     TTT GAT AAA CTT CAC AGG TTA TAT GTA GAT TTA ATT GCT CAA GGT ATC GAT CCG CAT GTT 
                F   D   K   L   H   R   L   Y   V   D   L   I   A   Q   G   I   D   P   H   V  
MN938851.1     TTT GAT AAA CTT CAC AGG TTA TAT GTA GAT TTA ATT GCT CAA GGT ATC GAT CCG CAT GTT 
                F   D   K   L   H   R   L   Y   V   D   L   I   A   Q   G   I   D   P   H   V  
LdIV1_JGS      TTT GAT AAA CTT CAC AGG TTG TAT GTA GAT TTA ATT GCT CAA GGT ATC GAT CCG CAC GTT 
                F   D   K   L   H   R   L   Y   V   D   L   I   A   Q   G   I   D   P   H   V  
LdIV1_ZY       TTT GAT AAA CTT CAC AGG TTG TAT GTA GAT TTA ATT GCT CAA GGT ATC GAT CCG CAC GTT 
                F   D   K   L   H   R   L   Y   V   D   L   I   A   Q   G   I   D   P   H   V  
LdIV1_NJ       TTT GAT AAA CTT CAC AGG TTG TAT GTA GAT TTA ATT GCT CAA GGT ATT GAT CCG CAC GTT 
                F   D   K   L   H   R   L   Y   V   D   L   I   A   Q   G   I   D   P   H   V  
LdIV1_CT       TTT GAT AAA CTT CAC AGG TTG TAT GTA GAT TTA ATT GCT CAA GGT ATT GAT CCG CAC GTT 
                F   D   K   L   H   K   L   Y   X   D   L   I   A   Q   G   V   D   P   H   V  
KJ629170.1     TTT GAT AAA CTT CAC AAG TTG TAT RTA GAT TTA ATT GCT CAA GGT GTT GAT CCG CAC GTT 
                                    ^   =       ^                           ^           =      


[1561..1580]
                R   K   L   P   F   V   I   Y   N   C   G   A   P   E   I   G   K   S   H   L  
MT753155.1     CGC AAG CTT CCA TTC GTC ATT TAT AAC TGT GGT GCT CCT GAG ATT GGT AAA AGT CAT CTT 
                R   K   L   P   F   V   I   Y   N   C   G   A   P   E   I   G   K   S   H   L  
MN938851.1     CGC AAG CTT CCA TTC GTC ATT TAT AAC TGT GGT GCT CCT GAG ATT GGT AAA AGT CAT CTT 
                R   K   L   P   F   V   I   Y   N   C   G   A   P   E   I   G   K   S   H   L  
LdIV1_JGS      CGC AAG CTT CCA TTT GTC ATC TAT AAC TGT GGT GCT CCT GAG ATT GGT AAA AGT CAT CTT 
                R   K   L   P   F   V   I   Y   N   C   G   A   P   E   I   G   K   S   H   L  
LdIV1_ZY       CGC AAG CTT CCA TTT GTC ATC TAT AAC TGT GGT GCT CCT GAG ATT GGT AAA AGT CAT CTT 
                R   K   L   P   F   V   I   Y   N   C   G   A   P   E   I   G   K   S   H   L  
LdIV1_NJ       CGC AAG CTT CCA TTT GTT ATC TAT AAC TGT GGT GCT CCT GAG ATT GGT AAA AGT CAT CTT 
                R   K   L   P   F   V   I   Y   N   C   G   A   P   E   I   G   K   S   H   L  
LdIV1_CT       CGC AAG CTT CCA TTT GTC ATC TAT AAC TGT GGT GCT CCT GAG ATT GGT AAA AGT CAT CTT 
                R   K   L   P   F   V   I   Y   N   C   G   A   P   E   I   G   K   S   H   L  
KJ629170.1     CGC AAG CTT CCA TTT GTC ATC TAT AAC TGT GGT GCT CCT GAG ATT GGT AAA AGT CAT CTT 
                                =   =   =                                                      


[1581..1600]
                T   T   N   I   C   A   E   L   C   K   D   Q   N   I   V   S   E   T   H   L  
MT753155.1     ACT ACA AAT ATC TGT GCT GAA TTA TGT AAA GAT CAG AAT ATA GTT TCT GAA ACT CAT TTA 
                T   T   N   I   C   A   E   L   C   K   D   Q   N   I   V   S   E   T   H   L  
MN938851.1     ACT ACA AAT ATC TGT GCT GAA TTA TGT AAA GAT CAG AAT ATA GTT TCT GAA ACT CAT TTA 
                T   T   N   I   C   A   E   L   C   K   D   Q   S   I   V   S   E   T   H   L  
LdIV1_JGS      ACT ACA AAT ATC TGT GCT GAA TTA TGT AAA GAC CAG AGT ATA GTT TCT GAA ACT CAT CTA 
                T   T   N   I   C   A   E   L   C   K   D   Q   S   I   V   S   E   T   H   L  
LdIV1_ZY       ACT ACA AAT ATC TGT GCT GAA TTA TGT AAA GAC CAG AGT ATA GTT TCT GAA ACT CAT CTA 
                T   T   N   I   C   A   E   L   C   K   D   Q   N   I   V   S   E   T   H   L  
LdIV1_NJ       ACT ACA AAT ATC TGT GCT GAA TTA TGT AAA GAT CAG AAT ATA GTT TCT GAA ACT CAT CTA 
                T   T   N   I   C   A   E   L   C   K   D   Q   S   I   V   S   E   T   H   L  
LdIV1_CT       ACT ACA AAT ATC TGT GCT GAA TTA TGT AAA GAT CAG AGT ATA GTT TCT GAA ACT CAT CTA 
                T   T   N   I   C   A   E   L   C   K   D   Q   D   I   V   S   E   T   H   L  
KJ629170.1     ACT ACA AAT ATC TGT GCT GAG TTA TGT AAA GAT CAG GAT ATA GTT TCT GAA ACT CAT CTA 
                                        =               =       ^                           =  


[1601..1620]
                M   C   V   L   N   A   T   S   K   F   W   D   N   C   D   R   Q   P   C   L  
MT753155.1     ATG TGT GTT TTG AAT GCA ACA TCG AAA TTT TGG GAC AAT TGC GAT CGC CAA CCA TGT TTG 
                M   C   V   L   N   A   T   S   K   F   W   D   N   C   D   R   Q   P   C   L  
MN938851.1     ATG TGT GTT TTG AAT GCA ACA TCG AAA TTT TGG GAC AAT TGC GAT CGC CAA CCA TGT TTG 
                M   C   V   L   N   A   T   S   K   F   W   D   N   C   D   R   Q   P   C   L  
LdIV1_JGS      ATG TGT GTC TTG AAT GCA ACA TCG AAA TTT TGG GAT AAT TGC GAT CGT CAA CCA TGT TTG 
                M   C   V   L   N   A   T   S   K   F   W   D   N   C   D   R   Q   P   C   L  
LdIV1_ZY       ATG TGT GTC TTG AAT GCA ACA TCG AAA TTT TGG GAT AAT TGC GAT CGT CAA CCA TGT TTG 
                M   C   V   L   N   A   T   S   K   F   W   D   N   C   D   R   Q   P   C   L  
LdIV1_NJ       ATG TGT GTT TTA AAT GCA ACA TCG AAA TTT TGG GAT AAT TGC GAT CGC CAA CCA TGT TTG 
                M   C   V   L   N   A   T   S   K   F   W   D   N   C   D   R   Q   P   C   L  
LdIV1_CT       ATG TGT GTT TTA AAT GCA ACA TCG AAA TTT TGG GAT AAT TGC GAT CGC CAA CCA TGT TTG 
                M   C   V   L   N   A   T   S   K   F   W   D   N   C   D   R   Q   P   C   L  
KJ629170.1     ATG TGT GTT TTA AAT GCA ACA TCG AAA TTT TGG GAT AAT TGC GAT CGC CAA CCA TGT TTG 
                        =   =                               =               =                  


[1621..1640]
                V   M   D   D   A   F   N   I   R   K   G   T   M   L   E   D   Q   L   A   A  
MT753155.1     GTT ATG GAC GAT GCC TTT AAT ATA CGT AAA GGA ACG ATG TTA GAA GAT CAA CTT GCA GCT 
                V   M   D   D   A   F   N   I   R   K   G   T   M   L   E   D   Q   L   A   A  
MN938851.1     GTT ATG GAC GAT GCC TTT AAT ATA CGT AAA GGA ACG ATG TTA GAA GAT CAA CTT GCA GCT 
                V   M   D   D   A   F   N   I   R   K   G   T   M   L   E   D   Q   L   A   A  
LdIV1_JGS      GTT ATG GAC GAT GCC TTT AAT ATA CGT AAA GGA ACG ATG TTA GAA GAT CAA CTT GCA GCT 
                V   M   D   D   A   F   N   I   R   K   G   T   M   L   E   D   Q   L   A   A  
LdIV1_ZY       GTT ATG GAC GAT GCC TTT AAT ATA CGT AAA GGA ACG ATG TTA GAA GAT CAA CTT GCA GCT 
                V   M   D   D   A   F   N   I   R   K   G   T   M   L   E   D   Q   L   A   A  
LdIV1_NJ       GTT ATG GAC GAT GCC TTT AAT ATA CGT AAA GGA ACG ATG TTA GAA GAT CAA CTT GCG GCT 
                V   M   D   D   A   F   N   I   R   K   G   T   M   L   E   D   Q   L   A   A  
LdIV1_CT       GTT ATG GAC GAT GCC TTT AAT ATA CGT AAA GGA ACG ATG TTA GAA GAT CAA CTT GCG GCT 
                V   M   D   D   A   F   N   I   R   K   G   T   M   L   E   D   Q   X   A   A  
KJ629170.1     GTT ATG GAC GAT GCC TTT AAT ATA CGT AAA GGA ACG ATG TTA GAA GAT CAA MTT GCG GCT 
                                                                                    ^   =      


[1641..1660]
                I   F   N   V   V   S   P   V   V   L   V   P   P   K   A   A   V   E   D   K  
MT753155.1     ATT TTC AAT GTA GTA TCA CCA GTT GTT TTA GTT CCC CCG AAA GCG GCT GTA GAA GAT AAA 
                I   F   N   V   V   S   P   V   V   L   V   P   P   K   A   A   V   E   D   K  
MN938851.1     ATT TTC AAT GTA GTA TCA CCA GTT GTT TTA GTT CCC CCG AAA GCG GCT GTA GAA GAT AAA 
                I   F   N   V   V   S   P   V   V   L   V   P   P   K   A   A   V   E   D   K  
LdIV1_JGS      ATT TTC AAT GTA GTA TCA CCA GTT GTT TTA GTT CCC CCG AAA GCG GCT GTA GAA GAT AAA 
                I   F   N   V   V   S   P   V   V   L   V   P   P   K   A   A   V   E   D   K  
LdIV1_ZY       ATT TTC AAT GTA GTA TCA CCA GTT GTT TTA GTT CCC CCG AAA GCG GCT GTA GAA GAT AAA 
                I   F   N   V   V   S   P   V   V   L   V   P   P   K   A   A   V   E   D   K  
LdIV1_NJ       ATT TTC AAT GTA GTA TCT CCA GTT GTT TTA GTT CCC CCG AAA GCG GCT GTA GAA GAT AAA 
                I   F   N   V   V   S   P   V   V   L   V   P   P   K   A   A   V   E   D   K  
LdIV1_CT       ATT TTC AAT GTA GTA TCT CCA GTT GTT TTA GTT CCC CCG AAA GCG GCT GTA GAA GAT AAA 
                I   F   N   V   V   S   P   V   V   L   V   P   P   K   A   A   V   E   D   K  
KJ629170.1     ATT TTC AAT GTA GTA TCT CCA GTT GTA TTA GTT CCC CCG AAA GCG GCT GTA GAA GAT AAA 
                                    =           =                                              


[1661..1680]
                G   R   T   Y   N   P   E   I   F   I   L   N   S   N   V   D   F   F   K   T  
MT753155.1     GGT CGT ACA TAT AAT CCA GAA ATA TTT ATA TTA AAT AGT AAT GTT GAT TTC TTT AAA ACA 
                G   R   T   Y   N   P   E   I   F   I   L   N   S   N   V   D   F   F   K   T  
MN938851.1     GGT CGT ACA TAT AAT CCA GAA ATA TTT ATA TTA AAT AGT AAT GTT GAT TTC TTT AAA ACA 
                G   R   T   Y   N   P   E   I   F   I   L   N   S   N   V   D   F   F   K   T  
LdIV1_JGS      GGT CGT ACA TAT AAT CCA GAA ATA TTT ATA TTA AAT AGT AAT GTT GAT TTC TTT AAA ACA 
                G   R   T   Y   N   P   E   I   F   I   L   N   S   N   V   D   F   F   K   T  
LdIV1_ZY       GGT CGT ACA TAT AAT CCA GAA ATA TTT ATA TTA AAT AGT AAT GTT GAT TTC TTT AAA ACA 
                G   R   T   Y   N   P   E   I   F   I   L   N   S   N   V   D   F   F   K   T  
LdIV1_NJ       GGT CGT ACA TAT AAT CCG GAA ATA TTT ATA TTA AAT AGT AAT GTC GAT TTC TTT AAA ACA 
                G   R   T   Y   N   P   E   I   F   I   L   N   S   N   V   D   F   F   K   T  
LdIV1_CT       GGT CGT ACA TAT AAT CCG GAA ATA TTT ATA TTA AAT AGT AAT GTC GAT TTC TTT AAA ACA 
                G   R   T   Y   N   P   E   I   F   I   L   N   S   N   V   D   F   F   K   T  
KJ629170.1     GGT CGT ACA TAT AAT CCG GAA ATA TTT ATA TTA AAT AGT AAT GTC GAT TTC TTT AAA ACA 
                                    =                                   =                      


[1681..1700]
                D   I   C   L   E   E   A   L   W   R   R   R   D   I   L   I   K   S   E   L  
MT753155.1     GAT ATT TGC TTG GAA GAA GCA TTG TGG CGT CGC AGA GAT ATA TTA ATT AAG AGC GAG TTA 
                D   I   C   L   E   E   A   L   W   R   R   R   D   I   L   I   K   S   E   L  
MN938851.1     GAT ATT TGC TTG GAA GAA GCA TTG TGG CGT CGC AGA GAT ATA TTA ATT AAG AGC GAG TTG 
                D   I   C   L   E   E   A   L   W   R   R   R   D   I   L   I   K   S   E   L  
LdIV1_JGS      GAT ATT TGC TTG GAA GAA GCA TTG TGG CGT CGC AGA GAT ATA TTA ATT AAA AGC GAG TTG 
                D   I   C   L   E   E   A   L   W   R   R   R   D   I   L   I   K   S   E   L  
LdIV1_ZY       GAT ATT TGC TTG GAA GAA GCA TTG TGG CGT CGC AGA GAT ATA TTA ATT AAA AGC GAG TTG 
                D   I   C   L   E   E   A   L   W   R   R   R   D   I   L   I   K   S   E   L  
LdIV1_NJ       GAT ATT TGC TTA GAA GAA GCA TTG TGG CGT CGC AGA GAT ATA TTA ATT AAA AGC GAG TTG 
                D   I   C   L   E   E   A   L   W   R   R   R   D   I   L   I   K   S   E   L  
LdIV1_CT       GAT ATT TGC TTA GAA GAA GCA TTG TGG CGT CGC AGA GAT ATA TTA ATT AAA AGC GAG TTG 
                D   I   C   L   E   E   A   L   W   R   R   R   D   I   L   I   K   S   E   L  
KJ629170.1     GAT ATT TGC TTA GAA GAA GCA TTG TGG CGT CGC AGA GAT ATA TTA ATT AAA AGC GAG TTG 
                            =                                                   =           =  


[1701..1720]
                D   P   D   F   V   K   E   G   C   I   H   C   L   K   K   L   K   V   S   S  
MT753155.1     GAC CCT GAC TTT GTT AAG GAA GGA TGT ATT CAT TGT TTA AAG AAA TTG AAA GTG AGT AGT 
                D   P   D   F   V   K   E   G   C   I   H   C   L   K   K   L   K   V   S   S  
MN938851.1     GAC CCT GAC TTT GTT AAG GAA GGA TGT ATT CAT TGT TTA AAG AAA TTG AAA GTG AGT AGT 
                D   P   D   F   V   K   E   G   C   I   H   C   L   K   K   L   K   V   S   S  
LdIV1_JGS      GAT CCT GAC TTT GTT AAG GAA GGT TGT ATT CAT TGT TTA AAG AAA TTG AAA GTG AGT AGT 
                D   P   D   F   V   K   E   G   C   I   H   C   L   K   K   L   K   V   S   S  
LdIV1_ZY       GAT CCT GAC TTT GTT AAG GAA GGT TGT ATT CAT TGT TTA AAG AAA TTG AAA GTG AGT AGT 
                D   P   D   F   V   K   E   G   C   I   H   C   L   K   K   L   K   V   S   S  
LdIV1_NJ       GAC CCT GAC TTT GTT AAG GAA GGA TGT ATT CAT TGT TTA AAG AAA TTG AAA GTT AGT AGT 
                D   P   D   F   V   K   E   G   C   I   H   C   L   K   K   L   K   V   S   S  
LdIV1_CT       GAC CCT GAC TTT GTT AAG GAA GGA TGT ATT CAT TGT TTA AAG AAA TTG AAA GTT AGT AGT 
                D   P   D   F   V   K   E   G   C   I   H   C   L   K   K   L   K   V   N   S  
KJ629170.1     GAC CCT GAC TTT GTT AAG GAA GGA TGT ATT CAT TGT TTA AAG AAA TTG AAA GTT AAT AGT 
                =                           =                                       =   ^      


[1721..1740]
                Q   L   P   V   E   A   V   T   A   L   K   D   N   H   H   L   K   F   K   Y  
MT753155.1     CAA TTG CCT GTT GAA GCT GTT ACA GCA TTG AAG GAT AAT CAT CAT CTT AAA TTT AAA TAT 
                Q   L   P   V   E   A   V   T   A   L   K   D   N   H   H   L   K   F   K   Y  
MN938851.1     CAA TTG CCT GTT GAA GCT GTT ACA GCA TTG AAG GAT AAT CAT CAT CTT AAA TTT AAA TAT 
                Q   L   P   V   E   A   V   T   A   L   K   D   N   H   H   L   K   F   K   Y  
LdIV1_JGS      CAA TTG CCT GTT GAA GCT GTT ACA GCA TTG AAG GAT AAT CAT CAT CTT AAA TTT AAA TAT 
                Q   L   P   V   E   A   V   T   A   L   K   D   N   H   H   L   K   F   K   Y  
LdIV1_ZY       CAA TTG CCT GTT GAA GCT GTT ACA GCA TTG AAG GAT AAT CAT CAT CTT AAA TTT AAA TAT 
                Q   L   P   V   E   A   V   T   A   L   K   D   N   H   H   L   K   F   K   Y  
LdIV1_NJ       CAA TTG CCT GTT GAA GCT GTT ACA GCA TTG AAG GAT AAT CAT CAT CTT AAA TTT AAA TAT 
                Q   L   P   V   E   A   V   T   A   L   K   D   N   H   H   L   K   F   K   Y  
LdIV1_CT       CAA TTG CCT GTT GAA GCT GTT ACA GCA TTG AAG GAT AAT CAT CAT CTT AAA TTT AAA TAT 
                Q   L   P   V   E   A   V   T   A   L   K   D   N   H   H   L   K   F   K   Y  
KJ629170.1     CAA TTG CCT GTT GAA GCT GTT ACA GCA TTG AAG GAT AAT CAT CAT CTT AAA TTT AAA TAT 
                                                                                               


[1741..1760]
                T   F   D   V   T   N   P   N   C   Q   Y   L   P   E   N   S   Y   L   K   Y  
MT753155.1     ACC TTT GAT GTT ACT AAT CCA AAT TGT CAA TAT TTA CCT GAG AAT AGT TAT TTG AAG TAC 
                T   F   D   V   T   N   P   N   C   Q   Y   L   P   E   N   S   Y   L   K   Y  
MN938851.1     ACC TTT GAT GTT ACT AAT CCA AAT TGT CAA TAT TTA CCT GAG AAT AGT TAT TTG AAG TAC 
                T   F   D   V   T   N   P   N   C   Q   Y   L   P   E   N   S   Y   L   K   Y  
LdIV1_JGS      ACC TTT GAT GTT ACT AAT CCA AAT TGT CAA TAT TTA CCT GAA AAT AGT TAT TTG AAG TAC 
                T   F   D   V   T   N   P   N   C   Q   Y   L   P   E   N   S   Y   L   K   Y  
LdIV1_ZY       ACC TTT GAT GTT ACT AAT CCA AAT TGT CAA TAT TTA CCT GAA AAT AGT TAT TTG AAG TAC 
                T   F   D   V   T   N   P   N   C   Q   Y   L   P   E   N   S   Y   L   K   Y  
LdIV1_NJ       ACC TTT GAT GTT ACT AAT CCA AAT TGT CAA TAT TTA CCT GAG AAT AGT TAT TTG AAG TAT 
                T   F   D   V   T   N   P   N   C   Q   Y   L   P   E   N   S   Y   L   K   Y  
LdIV1_CT       ACC TTT GAT GTT ACT AAT CCA AAT TGT CAA TAT TTA CCT GAG AAT AGT TAT TTA AAG TAT 
                T   F   D   V   T   N   P   N   C   Q   Y   L   P   E   N   S   Y   L   K   Y  
KJ629170.1     ACC TTT GAT GTT ACT AAT CCA AAT TGT CAA TAT TTA CCT GAG AAT AGT TAT TTG AAG TAT 
                                                                    =               =       =  


[1761..1780]
                D   E   L   M   K   L   L   K   D   L   F   K   K   N   R   E   A   E   N   Y  
MT753155.1     GAC GAA TTA ATG AAA TTA TTA AAA GAT TTA TTC AAG AAG AAC AGA GAA GCA GAG AAT TAT 
                D   E   L   M   K   L   L   K   D   L   F   K   K   N   R   E   A   E   N   Y  
MN938851.1     GAC GAA TTA ATG AAA TTA TTA AAA GAT TTA TTC AAG AAG AAC AGA GAA GCA GAA AAT TAT 
                D   E   L   M   K   L   L   K   D   L   F   K   K   N   R   E   A   E   N   Y  
LdIV1_JGS      GAT GAA TTA ATG AAA TTA CTA AAA GAT TTA TTC AAG AAG AAT AGA GAA GCA GAG AAT TAT 
                D   E   L   M   K   L   L   K   D   L   F   K   K   N   R   E   A   E   N   Y  
LdIV1_ZY       GAT GAA TTA ATG AAA TTA CTA AAA GAT TTA TTC AAG AAG AAT AGA GAA GCA GAG AAT TAT 
                D   E   L   M   K   L   L   K   D   L   F   K   K   N   R   E   A   E   N   Y  
LdIV1_NJ       GAT GAA TTA ATG AAA TTA TTA AAA GAT TTA TTC AAG AAG AAC AGA GAA GCA GAG AAT TAT 
                D   E   L   M   K   L   L   K   D   L   F   K   K   N   R   E   A   E   N   Y  
LdIV1_CT       GAT GAA TTA ATG AAA TTA TTA AAA GAT TTA TTC AAG AAG AAC AGA GAA GCA GAG AAT TAT 
                D   E   L   M   K   L   L   K   D   L   F   K   K   N   R   E   A   X   N   Y  
KJ629170.1     GAT GAA TTA ATG AAA TTA TTA AAA GAT TTA TTC AAG AAG AAC AGA GAA GCA KAG AAT TAT 
                =                       =                           =               ^          


[1781..1800]
                K   F   A   Q   R   V   A   H   C   N   E   V   S   S   N   F   P   S   L   V  
MT753155.1     AAA TTT GCG CAG CGA GTA GCC CAT TGT AAT GAA GTT TCT AGC AAT TTT CCT TCG TTG GTA 
                K   F   A   Q   R   V   A   H   C   N   E   V   S   S   N   F   P   S   L   V  
MN938851.1     AAA TTT GCG CAG CGA GTA GCC CAT TGT AAT GAA GTT TCT AGC AAT TTT CCT TCG TTG GTA 
                K   F   A   Q   R   V   A   H   C   N   E   V   S   S   N   F   P   S   L   V  
LdIV1_JGS      AAA TTT GCG CAG CGA GTA GCC CAT TGT AAT GAA GTT TCT AGC AAT TTT CCT TCG TTG GTA 
                K   F   A   Q   R   V   A   H   C   N   E   V   S   S   N   F   P   S   L   V  
LdIV1_ZY       AAA TTT GCG CAG CGA GTA GCC CAT TGT AAT GAA GTT TCT AGC AAT TTT CCT TCG TTG GTA 
                K   F   A   Q   R   V   A   H   C   N   E   V   S   S   S   F   P   S   L   V  
LdIV1_NJ       AAA TTT GCG CAG CGA GTA GCC CAT TGC AAT GAA GTT TCT AGT AGT TTT CCT TCG TTG GTA 
                K   F   A   Q   R   V   A   H   C   N   E   V   S   S   N   F   P   S   L   V  
LdIV1_CT       AAA TTT GCG CAG CGA GTA GCC CAT TGC AAT GAA GTT TCT AGT AAT TTT CCT TCG TTG GTA 
                K   F   A   Q   R   V   A   H   C   N   E   V   S   S   N   F   P   S   L   V  
KJ629170.1     AAA TTC GCG CAG CGA GTA GCC CAT TGC AAT GAA GTT TCT AGT AAT TTT CCT TCG TTG GTA 
                    =                           =                   =   ^                      


[1801..1820]
                K   H   V   D   D   L   E   D   L   W   N   Q   A   I   A   K   R   E   G   A  
MT753155.1     AAG CAT GTT GAT GAT CTT GAA GAT CTT TGG AAT CAG GCT ATA GCA AAA AGA GAA GGT GCC 
                K   H   V   D   D   L   E   D   L   W   N   Q   A   I   A   K   R   E   G   A  
MN938851.1     AAG CAT GTT GAT GAT CTT GAA GAT CTT TGG AAT CAG GCT ATA GCA AAA AGA GAA GGT GCC 
                K   H   V   D   D   L   E   D   L   W   N   Q   A   I   A   K   R   E   G   A  
LdIV1_JGS      AAG CAT GTT GAT GAT CTT GAG GAT CTT TGG AAT CAG GCT ATA GCA AAA AGA GAA GGT GCC 
                K   H   V   D   D   L   E   D   L   W   N   Q   A   I   A   K   R   E   G   A  
LdIV1_ZY       AAG CAT GTT GAT GAT CTT GAG GAT CTT TGG AAT CAG GCT ATA GCA AAA AGA GAA GGT GCC 
                K   H   V   D   D   L   E   D   L   W   N   Q   A   I   A   K   R   E   G   A  
LdIV1_NJ       AAG CAT GTT GAT GAT CTT GAA GAT CTT TGG AAT CAG GCT ATA GCA AAA AGA GAA GGT GCC 
                K   H   V   D   D   L   E   D   L   W   N   Q   A   I   A   K   R   E   G   A  
LdIV1_CT       AAG CAT GTT GAT GAT CTT GAA GAT CTT TGG AAT CAG GCT ATA GCA AAA AGA GAA GGT GCC 
                K   H   V   D   N   L   E   S   L   W   N   Q   A   I   Q   K   R   K   F   A  
KJ629170.1     AAG CAT GTT GAT AAT CTT GAA AGT CTT TGG AAT CAG GCT ATA CAA AAA AGA AAA TTT GCC 
                                ^       =   ^                           ^           ^   ^      


[1821..1840]
                V   N   L   V   K   N   S   T   L   M   S   M   S   K   G   F   A   E   K   I  
MT753155.1     GTA AAT CTT GTT AAA AAT AGC ACT TTA ATG TCA ATG TCA AAA GGC TTT GCA GAG AAA ATT 
                V   N   L   V   K   N   S   T   L   M   S   M   S   K   G   F   A   E   K   I  
MN938851.1     GTA AAT CTT GTT AAA AAT AGC ACT TTA ATG TCA ATG TCA AAA GGC TTT GCA GAG AAA ATT 
                V   N   L   V   K   N   S   T   L   M   S   M   S   K   G   F   A   E   K   I  
LdIV1_JGS      GTA AAT CTT GTA AAA AAT AGC ACT TTA ATG TCA ATG TCA AAA GGT TTT GCA GAA AAA ATT 
                V   N   L   V   K   N   S   T   L   M   S   M   S   K   G   F   A   E   K   I  
LdIV1_ZY       GTA AAT CTT GTA AAA AAT AGC ACT TTA ATG TCA ATG TCA AAA GGT TTT GCA GAA AAA ATT 
                V   N   L   V   K   N   S   T   L   M   S   M   S   K   G   F   A   E   K   I  
LdIV1_NJ       GTA AAT CTT GTT AAA AAT AGC ACT TTA ATG TCA ATG TCA AAA GGT TTT GCA GAA AAA ATT 
                V   N   L   V   K   N   S   T   L   M   S   M   S   K   G   F   A   E   K   I  
LdIV1_CT       GTA AAT CTT GTT AAA AAT AGC ACT TTA ATG TCA ATG TCA AAA GGT TTT GCA GAA AAA ATT 
                I   D   L   V   K   N   S   T   L   T   S   M   S   Q   S   F   X   E   K   I  
KJ629170.1     ATA GAT CTT GTT AAA AAT AGC ACT TTA ACG TCA ATG TCA CAA AGT TTT GYA GAG AAA ATT 
                ^   ^       =                       ^               ^   ^       ^   =          


[1841..1860]
                S   E   N   W   S   E   C   K   H   A   V   F   K   K   I   Y   T   S   I   R  
MT753155.1     TCC GAA AAT TGG TCA GAA TGC AAA CAT GCA GTT TTT AAA AAG ATT TAC ACT TCA ATA AGA 
                S   E   N   W   S   E   C   K   H   A   V   F   K   K   I   Y   T   S   I   R  
MN938851.1     TCC GAA AAT TGG TCA GAA TGC AAA CAT GCA GTT TTT AAA AAG ATT TAC ACT TCA ATA AGA 
                S   E   N   W   S   E   C   K   H   A   V   F   K   K   I   Y   T   S   I   R  
LdIV1_JGS      TCT GAA AAT TGG TCA GAA TGT AAA CAT GCA GTT TTT AAG AAA ATT TAT ACT TCA ATA AGA 
                S   E   N   W   S   E   C   K   H   A   V   F   K   K   I   Y   T   S   I   R  
LdIV1_ZY       TCT GAA AAT TGG TCA GAA TGT AAA CAT GCA GTT TTT AAG AAA ATT TAT ACT TCA ATA AGA 
                S   E   N   W   S   E   C   K   H   A   V   F   K   K   I   Y   T   S   I   R  
LdIV1_NJ       TCC GAA AAT TGG TCA GAA TGT AAA CAT GCA GTA TTT AAA AAG ATT TAT ACT TCG ATA AGA 
                S   E   N   W   S   E   C   K   H   A   V   F   K   K   I   Y   T   S   I   R  
LdIV1_CT       TCC GAA AAT TGG TCA GAA TGT AAA CAT GCA GTA TTT AAA AAG ATT TAT ACT TCG ATA AGA 
                S   E   N   W   S   E   C   K   H   A   V   F   K   K   I   Y   T   S   I   R  
KJ629170.1     TCC GAA AAT TGG TCA GAA TGT AAA CAT GCA GTA TTT AAA AAG ATT TAT ACT TCG ATA AGA 
                =                       =               =       =   =       =       =          


[1861..1880]
                P   G   V   N   K   Y   D   M   L   N   P   T   C   M   K   C   V   A   L   K  
MT753155.1     CCA GGA GTG AAT AAG TAT GAT ATG CTC AAT CCT ACG TGT ATG AAA TGT GTG GCG CTC AAA 
                P   G   V   N   K   Y   D   M   L   N   P   T   C   M   K   C   V   A   L   K  
MN938851.1     CCA GGA GTG AAT AAG TAT GAT ATG CTC AAT CCT ACG TGT ATG AAA TGT GTG GCG CTC AAA 
                P   G   V   N   K   Y   D   M   L   N   P   T   C   M   K   C   V   A   L   K  
LdIV1_JGS      CCG GGA GTG AAT AAG TAT GAT ATG CTC AAT CCT ACG TGT ATG AAA TGT GTA GCG CTT AAA 
                P   G   V   N   K   Y   D   M   L   N   P   T   C   M   K   C   V   A   L   K  
LdIV1_ZY       CCG GGA GTG AAT AAG TAT GAT ATG CTC AAT CCT ACG TGT ATG AAA TGT GTA GCG CTT AAA 
                P   G   V   N   K   Y   D   M   L   N   P   T   C   M   K   C   V   A   L   K  
LdIV1_NJ       CCG GGA GTG AAT AAA TAT GAT ATG CTT AAT CCT ACG TGT ATG AAA TGT GTG GCG CTT AAA 
                P   G   V   N   K   Y   D   M   L   N   P   T   C   M   K   C   V   A   L   K  
LdIV1_CT       CCG GGA GTG AAT AAA TAT GAT ATG CTT AAT CCT ACG TGT ATG AAA TGT GTG GCG CTT AAA 
                P   G   V   N   K   Y   D   M   L   N   P   T   C   M   K   C   V   A   L   K  
KJ629170.1     CCG GGA GTG AAT AAA TAT GAT ATG CTT AAT CCT ACG TGT ATG AAA TGT GTG GCG CTT AAA 
                =               =               =                               =       =      


[1881..1900]
                Y   Q   C   I   S   C   K   I   E   Y   E   K   M   K   K   E   A   N   T   I  
MT753155.1     TAT CAG TGT ATA TCG TGT AAA ATA GAA TAT GAG AAA ATG AAA AAA GAA GCT AAT ACA ATT 
                Y   Q   C   I   S   C   K   I   E   Y   E   K   M   K   K   E   A   N   T   I  
MN938851.1     TAT CAG TGT ATA TCG TGT AAA ATA GAA TAT GAG AAA ATG AAA AAA GAA GCT AAT ACA ATT 
                Y   Q   C   I   S   C   K   I   E   Y   E   K   M   K   K   E   A   N   T   I  
LdIV1_JGS      TAT CAG TGT ATA TCG TGT AAA ATA GAA TAT GAA AAA ATG AAA AAA GAA GCT AAT ACG ATT 
                Y   Q   C   I   S   C   K   I   E   Y   E   K   M   K   K   E   A   N   T   I  
LdIV1_ZY       TAT CAG TGT ATA TCG TGT AAA ATA GAA TAT GAA AAA ATG AAA AAA GAA GCT AAT ACG ATT 
                Y   Q   C   I   S   C   K   I   E   Y   E   K   M   K   K   E   A   N   I   V  
LdIV1_NJ       TAT CAG TGT ATA TCT TGT AAA ATA GAA TAT GAG AAA ATG AAA AAA GAA GCT AAT ATA GTT 
                Y   Q   C   I   S   C   K   I   E   Y   E   K   M   K   K   E   A   N   T   V  
LdIV1_CT       TAT CAG TGT ATA TCT TGT AAA ATA GAA TAT GAG AAA ATG AAA AAA GAA GCT AAT ACA GTT 
                Y   Q   C   I   S   C   K   I   E   Y   E   K   M   K   K   E   A   N   T   V  
KJ629170.1     TAT CAG TGT ATA TCT TGT AAA ATA GAA TAT GAG AAA ATG AAA AAA GAA GCT AAT ACA GTT 
                                =                       =                               ^   ^  


[1901..1920]
                D   T   P   T   P   S   T   S   S   G   S   S   S   V   E   V   L   F   G   D  
MT753155.1     GAT ACA CCT ACT CCA TCA ACA AGT AGT GGT TCT TCC TCC GTT GAA GTT TTA TTT GGA GAT 
                D   T   P   T   P   S   T   S   S   G   S   S   S   V   E   V   L   F   G   D  
MN938851.1     GAT ACA CCT ACT CCA TCA ACA AGT AGT GGT TCT TCC TCC GTT GAA GTT TTA TTT GGA GAT 
                D   T   P   T   P   S   T   S   S   G   S   S   S   V   E   V   L   F   G   D  
LdIV1_JGS      GAT ACA CCT ACT CCA TCG ACA AGT AGT GGT TCT TCC TCC GTT GAA GTA TTA TTT GGA GAT 
                D   T   P   T   P   S   T   S   S   G   S   S   S   V   E   V   L   F   G   D  
LdIV1_ZY       GAT ACA CCT ACT CCA TCG ACA AGT AGT GGT TCT TCC TCC GTT GAA GTA TTA TTT GGA GAT 
                D   T   P   T   P   S   T   S   S   G   S   S   S   V   E   V   L   F   G   D  
LdIV1_NJ       GAT ACA CCT ACT CCA TCG ACA AGT AGT GGT TCT TCC TCC GTT GAA GTT TTA TTT GGA GAT 
                D   T   P   T   P   S   T   S   S   G   S   S   S   V   E   V   L   F   G   D  
LdIV1_CT       GAT ACA CCT ACT CCA TCG ACA AGT AGT GGT TCT TCC TCC GTT GAA GTT TTA TTT GGA GAT 
                D   T   P   I   P   S   T   S   S   G   S   A   S   I   E   V   L   F   G   D  
KJ629170.1     GAT ACA CCT ATT CCA TCG ACA AGT AGT GGT TCT GCC TCC ATT GAA GTT TTA TTT GGA GAT 
                            ^       =                       ^       ^       =                  


[1921..1940]
                P   K   I   G   Y   Q   G   N   A   P   I   V   E   E   P   A   D   N   T   N  
MT753155.1     CCT AAG ATA GGG TAT CAA GGT AAT GCG CCA ATC GTG GAA GAA CCA GCT GAT AAT ACT AAT 
                P   K   I   G   Y   Q   G   N   A   P   I   V   E   E   P   A   D   N   T   N  
MN938851.1     CCT AAG ATA GGG TAT CAA GGT AAT GCG CCA ATC GTG GAA GAA CCA GCT GAT AAT ACT AAT 
                P   K   I   G   Y   Q   G   N   A   P   I   V   E   E   P   A   D   N   T   S  
LdIV1_JGS      CCT AAG ATA GGG TAT CAA GGT AAT GCG CCA ATC GTA GAA GAA CCA GCT GAT AAT ACT TCT 
                P   K   I   G   Y   Q   G   N   A   P   I   V   E   E   P   A   D   N   T   S  
LdIV1_ZY       CCT AAG ATA GGG TAT CAA GGT AAT GCG CCA ATC GTA GAA GAA CCA GCT GAT AAT ACT TCT 
                P   K   I   G   Y   Q   G   N   A   P   I   I   E   E   P   A   D   N   T   N  
LdIV1_NJ       CCT AAG ATA GGG TAT CAA GGT AAT GCG CCA ATC ATA GAA GAA CCA GCT GAT AAT ACT AAT 
                P   K   I   G   Y   Q   G   N   A   P   I   I   E   E   P   A   D   N   T   N  
LdIV1_CT       CCT AAG ATA GGG TAT CAA GGT AAT GCG CCA ATC ATA GAA GAA CCA GCT GAT AAT ACT AAT 
                P   K   L   G   Y   Q   G   N   A   P   I   V   E   E   P   A   D   N   T   D  
KJ629170.1     CCT AAG CTA GGG TAT CAA GGT AAT GCG CCA ATC GTA GAG GAA CCA GCT GAT AAT ACT GAT 
                        ^                                   ^   =                           ^  


[1941..1960]
                T   S   I   P   V   S   I   P   M   F   D   H   D   V   K   Y   L   L   S   S  
MT753155.1     ACT TCT ATT CCA GTG TCA ATT CCA ATG TTT GAT CAC GAT GTC AAG TAT TTG CTT AGT AGT 
                T   S   I   P   V   S   I   P   M   F   D   H   D   V   K   Y   L   L   S   S  
MN938851.1     ACT TCT ATT CCA GTG TCA ATT CCA ATG TTT GAT CAT GAT GTC AAG TAT TTG CTT AGT AGT 
                -   -   I   P   V   A   I   P   M   F   D   H   D   V   K   Y   L   L   S   S  
LdIV1_JGS      --- --- ATT CCA GTG GCA ATT CCA ATG TTT GAT CAT GAT GTC AAG TAT TTG CTT AGT AGT 
                -   -   I   P   V   A   I   P   M   F   D   H   D   V   K   Y   L   L   S   S  
LdIV1_ZY       --- --- ATT CCA GTG GCA ATT CCA ATG TTT GAT CAT GAT GTC AAG TAT TTG CTT AGT AGT 
                T   S   I   P   V   S   I   P   M   F   D   H   D   V   K   Y   L   L   S   S  
LdIV1_NJ       ACT TCT ATT CCA GTG TCA ATT CCA ATG TTC GAT CAT GAT GTC AAG TAT TTG CTT AGT AGT 
                T   S   I   P   V   S   I   P   M   F   D   H   D   V   K   Y   L   L   S   S  
LdIV1_CT       ACT TCT ATT CCA GTG TCA ATT CCA ATG TTC GAT CAT GAT GTC AAG TAT TTG CTT AGT AGT 
                -   -   -   -   I   A   V   P   V   F   D   H   D   V   K   Y   L   L   S   S  
KJ629170.1     --- --- --- --- ATT GCA GTT CCA GTG TTC GAT CAT GAT GTC AAG TAT TTG CTT AGT AGT 
                ^   ^   ^   ^   ^   ^   ^       ^   =       =                                  


[1961..1980]
                K   G   C   E   W   L   T   D   L   K   K   N   Y   S   P   I   V   L   H   D  
MT753155.1     AAA GGG TGT GAG TGG TTA ACA GAT TTA AAA AAG AAT TAC TCA CCT ATA GTT TTG CAT GAT 
                K   G   C   E   W   L   T   D   L   K   K   N   Y   S   P   I   V   L   H   D  
MN938851.1     AAA GGG TGT GAG TGG TTA ACA GAT TTA AAA AAG AAT TAC TCA CCT ATA GTT TTG CAT GAT 
                K   G   C   E   W   L   T   D   L   K   K   N   Y   S   P   I   V   L   H   D  
LdIV1_JGS      AAA GGG TGT GAA TGG TTA ACA GAT TTA AAA AAG AAT TAC TCA CCT ATA GTC TTG CAT GAT 
                K   G   C   E   W   L   T   D   L   K   K   N   Y   S   P   I   V   L   H   D  
LdIV1_ZY       AAA GGG TGT GAA TGG TTA ACA GAT TTA AAA AAG AAT TAC TCA CCT ATA GTC TTG CAT GAT 
                K   G   C   E   W   L   T   D   L   K   K   N   Y   S   P   I   V   L   H   D  
LdIV1_NJ       AAA GGG TGT GAG TGG TTA ACA GAT TTA AAA AAG AAT TAC TCA CCT ATA GTT TTG CAT GAT 
                K   G   C   E   W   L   T   D   L   K   K   N   Y   S   P   I   V   L   H   D  
LdIV1_CT       AAA GGG TGT GAG TGG TTA ACA GAT CTA AAA AAG AAT TAC TCA CCT ATA GTT TTG CAT GAT 
                K   G   C   K   W   L   T   D   L   N   N   N   Y   S   P   I   V   L   H   D  
KJ629170.1     AAA GGG TGT AAG TGG TTA ACA GAT TTA AAC AAT AAT TAC TCA CCT ATA GTT TTG CAT GAT 
                            ^                   =   ^   ^                       =              


[1981..2000]
                F   K   K   F   L   D   D   H   H   D   S   I   A   V   S   L   R   R   Y   P  
MT753155.1     TTT AAG AAA TTT TTA GAT GAT CAT CAT GAT AGT ATA GCT GTC AGT CTG CGA CGG TAT CCT 
                F   K   K   F   L   D   D   H   H   D   S   I   A   V   S   L   R   R   Y   P  
MN938851.1     TTT AAG AAA TTT TTA GAT GAT CAT CAT GAT AGT ATA GCT GTC AGT CTG CGA CGG TAT CCT 
                F   K   K   F   L   D   D   H   H   D   S   I   A   V   S   L   R   R   Y   P  
LdIV1_JGS      TTT AAA AAA TTT TTA GAT GAT CAT CAT GAT AGT ATA GCT GTC AGT CTG CGA CGG TAT CCT 
                F   K   K   F   L   D   D   H   H   D   S   I   A   V   S   L   R   R   Y   P  
LdIV1_ZY       TTT AAA AAA TTT TTA GAT GAT CAT CAT GAT AGT ATA GCT GTC AGT CTG CGA CGG TAT CCT 
                F   K   K   F   L   D   D   H   H   D   S   I   A   V   S   L   R   R   Y   P  
LdIV1_NJ       TTT AAG AAA TTT TTA GAT GAT CAT CAT GAT AGC ATA GCT GTC AGT CTG CGA CGA TAT CCT 
                F   K   K   F   L   D   D   H   H   D   S   I   A   V   S   L   R   R   Y   P  
LdIV1_CT       TTT AAG AAA TTT TTA GAT GAT CAT CAT GAT AGC ATA GCT GTC AGT CTG CGA CGA TAT CCT 
                F   K   K   F   L   D   D   H   H   D   S   I   A   V   S   L   R   R   Y   P  
KJ629170.1     TTT AAG AAA TTT TTA GAT GAT CAT CAT GAT AGC ATA GCT GTC AGT CTG CGA CGA TAT CCT 
                    =                                   =                           =          


[2001..2020]
                Q   Y   A   R   G   P   E   L   F   K   S   I   C   Q   R   G   C   N   C   V  
MT753155.1     CAA TAC GCG CGT GGG CCA GAA CTT TTT AAA TCC ATA TGT CAA CGT GGG TGT AAT TGT GTT 
                Q   Y   A   R   G   P   E   L   F   K   S   I   C   Q   R   G   C   N   C   V  
MN938851.1     CAA TAC GCG CGT GGG CCA GAA CTT TTT AAA TCC ATA TGT CAA CGT GGG TGT AAT TGT GTT 
                Q   Y   A   R   G   P   E   L   F   K   S   I   C   Q   R   G   C   D   C   V  
LdIV1_JGS      CAA TAT GCG CGT GGG CCA GAA TTG TTT AAA TCC ATA TGT CAA CGT GGG TGT GAT TGT GTT 
                Q   Y   A   R   G   P   E   L   F   K   S   I   C   Q   R   G   C   D   C   V  
LdIV1_ZY       CAA TAT GCG CGT GGG CCA GAA TTG TTT AAA TCC ATA TGT CAA CGT GGG TGT GAT TGT GTT 
                Q   Y   A   R   G   P   E   L   F   K   S   I   C   Q   R   G   C   N   C   V  
LdIV1_NJ       CAA TAT GCG CGT GGG CCA GAA TTG TTT AAA TCT ATA TGT CAA CGT GGG TGT AAT TGT GTT 
                Q   Y   A   R   G   P   E   L   F   K   S   I   C   Q   R   G   C   N   C   V  
LdIV1_CT       CAA TAT GCG CGT GGG CCA GAA TTG TTT AAA TCT ATA TGT CAA CGT GGG TGT AAT TGT GTT 
                Q   Y   A   R   G   P   E   L   F   K   S   I   C   E   S   G   C   D   C   V  
KJ629170.1     CAA TAT GCG CGT GGG CCA GAA TTG TTT AAA TCT ATA TGT GAA AGT GGG TGT GAT TGT GTT 
                    =                       =           =           ^   ^           ^          


[2021..2040]
                H   N   F   N   T   N   P   P   I   I   Y   K   G   Q   F   A   F   V   N   P  
MT753155.1     CAT AAT TTT AAT ACT AAT CCT CCT ATA ATA TAT AAG GGT CAA TTT GCT TTT GTT AAT CCG 
                H   N   F   N   T   N   P   P   I   I   Y   K   G   Q   F   A   F   V   N   P  
MN938851.1     CAT AAT TTT AAT ACT AAT CCT CCT ATA ATA TAT AAG GGT CAA TTT GCT TTT GTT AAT CCG 
                H   N   F   N   T   N   P   P   I   I   Y   K   G   Q   F   A   F   V   N   P  
LdIV1_JGS      CAT AAT TTT AAT ACT AAT CCT CCT ATA ATA TAT AAG GGT CAA TTT GCT TTT GTT AAT CCG 
                H   N   F   N   T   N   P   P   I   I   Y   K   G   Q   F   A   F   V   N   P  
LdIV1_ZY       CAT AAT TTT AAT ACT AAT CCT CCT ATA ATA TAT AAG GGT CAA TTT GCT TTT GTT AAT CCG 
                H   N   F   N   T   N   P   P   I   I   Y   K   G   Q   F   A   F   V   N   P  
LdIV1_NJ       CAT AAT TTT AAT ACT AAT CCT CCT ATA ATA TAT AAG GGT CAA TTT GCT TTT GTT AAT CCG 
                H   N   F   N   A   N   P   P   I   I   Y   K   G   Q   F   A   F   V   N   P  
LdIV1_CT       CAT AAT TTT AAT GCT AAT CCT CCT ATA ATA TAT AAG GGT CAA TTT GCT TTT GTT AAT CCG 
                H   N   F   N   K   N   P   P   I   I   Y   K   G   Q   F   A   F   V   N   P  
KJ629170.1     CAT AAT TTT AAT AAG AAT CCT CCT ATA ATA TAT AAG GGT CAA TTT GCT TTT GTT AAT CCG 
                                ^                                                              


[2041..2060]
                S   T   P   G   V   P   D   Y   I   N   C   F   T   C   T   G   K   C   W   M  
MT753155.1     TCT ACA CCT GGC GTG CCT GAT TAT ATC AAT TGC TTC ACA TGT ACG GGG AAG TGT TGG ATG 
                S   T   P   G   V   P   D   Y   I   N   C   F   T   C   T   G   K   C   W   M  
MN938851.1     TCT ACA CCT GGC GTG CCT GAT TAT ATC AAT TGC TTC ACA TGT ACG GGG AAG TGT TGG ATG 
                S   T   P   G   V   P   D   Y   I   N   C   F   T   C   T   G   K   C   W   M  
LdIV1_JGS      TCT ACA CCT GGT GTG CCC GAT TAT ATT AAT TGC TTT ACA TGT ACG GGA AAG TGT TGG ATG 
                S   T   P   G   V   P   D   Y   I   N   C   F   T   C   T   G   K   C   W   M  
LdIV1_ZY       TCT ACA CCT GGT GTG CCC GAT TAT ATT AAT TGC TTT ACA TGT ACG GGA AAG TGT TGG ATG 
                S   T   P   G   V   P   D   Y   I   N   C   F   T   C   T   G   K   C   W   M  
LdIV1_NJ       TCT ACA CCT GGT GTG CCC GAT TAT ATT AAT TGC TTT ACA TGT ACG GGA AAG TGT TGG ATG 
                S   T   P   G   V   P   D   Y   I   N   C   F   T   C   T   G   K   C   W   M  
LdIV1_CT       TCT ACA CCT GGT GTG CCC GAT TAT ATT AAT TGC TTT ACA TGT ACG GGA AAG TGT TGG ATG 
                S   T   P   G   V   P   D   Y   I   N   C   F   T   C   T   G   K   C   W   M  
KJ629170.1     TCT ACA CCT GGT GTG CCC GAT TAT ATT AAT TGC TTT ACA TGT ACG GGA AAG TGT TGG ATG 
                            =       =           =           =               =                  


[2061..2080]
                L   L   P   W   I   H   H   G   T   V   I   A   C   K   K   M   S   A   S   R  
MT753155.1     TTA CTA CCT TGG ATT CAT CAT GGT ACT GTC ATT GCA TGT AAG AAA ATG AGT GCA TCG AGA 
                L   L   P   W   I   H   H   G   T   V   I   A   C   K   K   M   S   A   S   R  
MN938851.1     TTA CTA CCT TGG ATT CAT CAT GGT ACT GTC ATT GCA TGT AAG AAA ATG AGT GCA TCG AGA 
                L   L   P   W   I   H   H   G   T   V   I   A   C   K   K   M   S   A   S   R  
LdIV1_JGS      TTG TTA CCT TGG ATT CAT CAT GGT ACT GTT ATT GCA TGT AAG AAA ATG AGT GCA TCG AGA 
                L   L   P   W   I   H   H   G   T   V   I   A   C   K   K   M   S   A   S   R  
LdIV1_ZY       TTG TTA CCT TGG ATT CAT CAT GGT ACT GTT ATT GCA TGT AAG AAA ATG AGT GCA TCG AGA 
                L   L   P   W   I   H   H   G   T   V   I   A   C   K   K   M   S   A   S   R  
LdIV1_NJ       TTG TTA CCT TGG ATT CAT CAT GGT ACT GTT ATT GCA TGT AAG AAA ATG AGT GCA TCA AGA 
                L   L   P   W   I   H   H   G   T   V   I   A   C   K   K   M   S   A   S   R  
LdIV1_CT       TTG TTA CCT TGG ATT CAT CAT GGT ACT GTT ATT GCA TGT AAG AAA ATG AGT GCA TCA AGA 
                L   L   P   W   I   H   H   G   T   V   I   A   C   K   K   M   S   A   L   R  
KJ629170.1     TTG TTA CCT TGG ATT CAT CAT GGT ACT GTT ATT GCA TGT AAG AAG ATG AGT GCA TTA AGA 
                =   =                               =                   =               ^      


[2081..2100]
                E   S   W   M   C   E   M   D   D   R   E   L   I   F   D   K   I   T   F   N  
MT753155.1     GAG AGT TGG ATG TGT GAG ATG GAT GAT AGA GAA TTG ATA TTT GAT AAG ATT ACG TTT AAT 
                E   S   W   M   C   E   M   D   D   R   E   L   I   F   D   K   I   T   F   N  
MN938851.1     GAG AGT TGG ATG TGT GAG ATG GAT GAT AGA GAA TTG ATA TTT GAT AAG ATT ACG TTT AAT 
                E   S   W   M   C   E   M   D   D   R   E   L   I   F   D   K   I   T   F   D  
LdIV1_JGS      GAG AGT TGG ATG TGT GAG ATG GAT GAT AGA GAA TTG ATA TTT GAT AAA ATT ACG TTT GAT 
                E   S   W   M   C   E   M   D   D   R   E   L   I   F   D   K   I   T   F   D  
LdIV1_ZY       GAG AGT TGG ATG TGT GAG ATG GAT GAT AGA GAA TTG ATA TTT GAT AAA ATT ACG TTT GAT 
                E   S   W   M   C   E   M   D   D   R   E   L   I   F   D   K   I   T   F   N  
LdIV1_NJ       GAG AGT TGG ATG TGT GAG ATG GAT GAT AGA GAA TTG ATA TTT GAT AAA ATT ACG TTT AAT 
                E   S   W   M   C   E   M   D   D   R   E   L   I   F   D   K   I   T   F   N  
LdIV1_CT       GAG AGT TGG ATG TGT GAG ATG GAT GAT AGA GAA TTG ATA TTT GAT AAA ATT ACG TTT AAT 
                E   S   W   M   C   Q   M   D   D   R   E   L   I   F   D   K   I   T   F   D  
KJ629170.1     GAG AGT TGG ATG TGT CAG ATG GAT GAT AGA GAA TTG ATA TTT GAT AAA ATT ACG TTT GAT 
                                    ^                                       =               ^  


[2101..2120]
                S   I   F   S   K   L   T   K   W   V   W   D   F   Y   Y   D   K   M   K   P  
MT753155.1     AGC ATA TTT TCA AAA TTA ACT AAG TGG GTT TGG GAT TTC TAT TAT GAT AAG ATG AAA CCT 
                S   I   F   S   K   L   T   K   W   V   W   D   F   Y   Y   D   K   M   K   P  
MN938851.1     AGT ATA TTT TCA AAA TTA ACT AAG TGG GTT TGG GAT TTC TAT TAT GAT AAG ATG AAA CCT 
                S   I   F   S   K   L   T   K   W   V   W   D   F   Y   Y   D   K   M   K   P  
LdIV1_JGS      AGT ATA TTT TCA AAA TTA ACT AAG TGG GTT TGG GAT TTC TAT TAT GAT AAG ATG AAA CCT 
                S   I   F   S   K   L   T   K   W   V   W   D   F   Y   Y   D   K   M   K   P  
LdIV1_ZY       AGT ATA TTT TCA AAA TTA ACT AAG TGG GTT TGG GAT TTC TAT TAT GAT AAG ATG AAA CCT 
                S   I   F   S   K   L   T   K   W   V   W   D   F   Y   Y   D   K   M   K   P  
LdIV1_NJ       AGT ATA TTT TCA AAA TTA ACT AAG TGG GTT TGG GAT TTC TAT TAT GAT AAG ATG AAA CCT 
                S   I   F   S   K   L   T   K   W   V   W   D   F   Y   Y   D   K   M   K   P  
LdIV1_CT       AGT ATA TTT TCA AAA TTA ACT AAG TGG GTT TGG GAT TTC TAT TAT GAT AAG ATG AAA CCT 
                S   I   F   S   K   L   T   K   W   V   W   D   F   Y   Y   D   K   M   K   P  
KJ629170.1     AGT ATA TTT TCA AAA TTA ACT AAG TGG GTT TGG GAT TTC TAT TAT GAT AAG ATG AAA CCT 
                =                                                                              


[2121..2140]
                A   L   K   A   V   I   S   F   F   S   T   L   N   G   W   M   F   G   A   L  
MT753155.1     GCT CTT AAA GCT GTA ATA TCA TTC TTT TCT ACT CTT AAC GGG TGG ATG TTT GGT GCT CTA 
                A   L   K   A   V   I   S   F   F   S   T   L   N   G   W   M   F   G   A   L  
MN938851.1     GCT CTT AAA GCT GTA ATA TCA TTC TTT TCT ACT CTT AAC GGG TGG ATG TTT GGT GCT CTA 
                A   L   K   A   V   I   S   F   F   S   T   L   N   G   W   M   F   G   A   L  
LdIV1_JGS      GCT CTT AAA GCT GTA ATA TCA TTC TTT TCT ACT CTT AAC GGG TGG ATG TTT GGT GCT CTA 
                A   L   K   A   V   I   S   F   F   S   T   L   N   G   W   M   F   G   A   L  
LdIV1_ZY       GCT CTT AAA GCT GTA ATA TCA TTC TTT TCT ACT CTT AAC GGG TGG ATG TTT GGT GCT CTA 
                A   L   K   A   V   I   S   F   F   S   T   L   N   G   W   M   F   G   A   L  
LdIV1_NJ       GCT CTT AAA GCT GTA ATA TCA TTC TTT TCT ACT CTT AAC GGG TGG ATG TTT GGT GCT CTA 
                A   L   K   A   V   I   S   F   F   S   T   L   N   G   W   M   F   G   A   L  
LdIV1_CT       GCT CTT AAA GCT GTA ATA TCA TTC TTT TCT ACT CTT AAC GGG TGG ATG TTT GGT GCT CTA 
                A   L   K   A   V   I   S   F   F   S   T   L   N   G   W   M   F   G   A   L  
KJ629170.1     GCT CTT AAA GCT GTA ATA TCA TTC TTT TCT ACT CTT AAC GGG TGG ATG TTC GGT GCT CTA 
                                                                                =              


[2141..2160]
                F   L   T   T   I   F   S   T   I   I   M   G   V   G   T   Y   E   V   C   T  
MT753155.1     TTC TTA ACA ACT ATA TTT TCA ACC ATT ATT ATG GGT GTT GGT ACA TAT GAA GTA TGT ACA 
                F   L   T   T   I   F   S   T   I   I   M   G   V   G   T   Y   E   V   C   T  
MN938851.1     TTC TTA ACA ACT ATA TTT TCA ACC ATT ATT ATG GGT GTT GGT ACA TAT GAA GTA TGT ACA 
                F   L   T   T   I   F   S   T   I   I   M   G   V   G   T   Y   E   V   C   T  
LdIV1_JGS      TTC TTA ACA ACT ATA TTT TCA ACC ATT ATT ATG GGT GTT GGT ACA TAT GAA GTA TGT ACA 
                F   L   T   T   I   F   S   T   I   I   M   G   V   G   T   Y   E   V   C   T  
LdIV1_ZY       TTC TTA ACA ACT ATA TTT TCA ACC ATT ATT ATG GGT GTT GGT ACA TAT GAA GTA TGT ACA 
                F   L   T   T   I   F   S   T   I   I   M   G   V   G   T   Y   E   V   C   T  
LdIV1_NJ       TTC TTA ACA ACT ATA TTT TCA ACT ATT ATT ATG GGT GTT GGT ACA TAT GAA GTA TGT ACA 
                F   L   T   T   I   F   S   T   I   I   M   G   V   G   T   Y   E   V   C   T  
LdIV1_CT       TTC TTA ACA ACT ATA TTT TCA ACT ATT ATT ATG GGT GTT GGT ACA TAT GAA GTA TGT ACA 
                F   L   T   T   I   F   S   T   I   I   M   G   V   G   T   Y   E   V   C   T  
KJ629170.1     TTC TTA ACA ACT ATA TTT TCA ACT ATT ATT ATG GGT GTT GGT ACA TAT GAA GTA TGT ACA 
                                            =                                                  


[2161..2180]
                Q   Q   G   G   M   Q   P   S   G   A   E   I   A   R   A   N   T   I   K   T  
MT753155.1     CAG CAA GGA GGA ATG CAA CCG AGT GGT GCA GAA ATA GCA CGT GCC AAT ACT ATA AAA ACT 
                Q   Q   G   G   M   Q   P   S   G   A   E   I   A   R   A   N   T   I   K   T  
MN938851.1     CAG CAA GGA GGA ATG CAA CCG AGT GGT GCA GAA ATA GCA CGT GCC AAT ACT ATA AAA ACT 
                Q   Q   G   G   L   Q   P   S   G   A   E   I   A   R   A   N   T   I   K   T  
LdIV1_JGS      CAG CAG GGA GGA TTG CAA CCG AGT GGT GCA GAA ATA GCA CGT GCG AAT ACC ATA AAG ACT 
                Q   Q   G   G   L   Q   P   S   G   A   E   I   A   R   A   N   T   I   K   T  
LdIV1_ZY       CAG CAG GGA GGA TTG CAA CCG AGT GGT GCA GAA ATA GCA CGT GCG AAT ACC ATA AAG ACT 
                Q   Q   G   G   L   Q   P   S   G   A   E   I   A   R   A   N   T   I   K   T  
LdIV1_NJ       CAG CAG GGA GGA TTG CAA CCG AGT GGT GCA GAA ATA GCA CGT GCG AAT ACC ATA AAG ACT 
                Q   Q   G   G   L   Q   P   S   G   A   E   I   A   R   A   N   T   I   K   T  
LdIV1_CT       CAG CAG GGA GGA TTG CAA CCG AGT GGT GCA GAA ATA GCA CGT GCG AAT ACC ATA AAG ACT 
                Q   Q   G   G   L   Q   P   S   G   A   E   I   A   R   A   N   T   I   K   T  
KJ629170.1     CAG CAG GGA GGA TTG CAA CCG AGT GGT GCA GAA ATA GCA CGT GCG AAT ACC ATA AAG ACT 
                    =           ^                                       =       =       =      


[2181..2200]
                V   G   D   T   K   F   H   P   I   A   Y   Q   A   N   S   Y   E   S   G   K  
MT753155.1     GTT GGT GAC ACA AAA TTT CAC CCT ATT GCT TAC CAA GCT AAC TCA TAT GAA TCT GGG AAA 
                V   G   D   T   K   F   H   P   I   A   Y   Q   A   N   S   Y   E   S   G   K  
MN938851.1     GTT GGT GAC ACA AAA TTT CAC CCT ATT GCT TAC CAA GCT AAC TCA TAT GAA TCT GGG AAA 
                V   G   D   T   K   L   H   P   I   A   Y   Q   A   N   S   Y   E   S   G   K  
LdIV1_JGS      GTT GGC GAC ACA AAA TTA CAT CCT ATT GCT TAC CAA GCT AAT TCA TAT GAG TCT GGA AAG 
                V   G   D   T   K   L   H   P   I   A   Y   Q   A   N   S   Y   E   S   G   K  
LdIV1_ZY       GTT GGC GAC ACA AAA TTA CAT CCT ATT GCT TAC CAA GCT AAT TCA TAT GAG TCT GGA AAG 
                V   G   D   T   K   F   H   P   I   A   Y   Q   A   N   S   Y   E   S   G   K  
LdIV1_NJ       GTT GGC GAC ACA AAA TTT CAT CCT ATT GCT TAC CAA GCT AAT TCA TAC GAG TCT GGA AAG 
                V   G   D   T   K   F   H   P   I   A   Y   Q   A   N   S   Y   E   S   G   K  
LdIV1_CT       GTT GGT GAC ACA AAA TTT CAT CCT ATT GCT TAC CAA GCT AAT TCA TAT GAG TCT GGA AAG 
                V   G   D   T   K   F   H   P   I   A   Y   Q   A   N   S   Y   E   S   G   K  
KJ629170.1     GTT GGT GAC ACG AAA TTT CAT CCT ATT GCT TAC CAA GCT AAT TCA TAT GAG TCT GGA AAG 
                    =       =       ^   =                           =       =   =       =   =  


[2201..2220]
                P   R   V   A   K   A   S   K   A   K   I   K   T   P   I   R   A   T   K   T  
MT753155.1     CCT CGT GTT GCC AAA GCA TCA AAA GCC AAA ATT AAA ACA CCT ATA CGA GCC ACG AAA ACA 
                P   R   V   A   K   A   S   K   A   K   I   K   T   P   I   R   A   T   K   T  
MN938851.1     CCT CGT GTT GCC AAA GCA TCA AAA GCC AAA ATT AAA ACA CCT ATA CGA GCC ACG AAA ACA 
                P   R   V   A   K   A   S   K   A   K   I   K   T   P   I   R   A   T   K   T  
LdIV1_JGS      CCT CGT GTT GCC AAA GCG TCA AAA GCT AAA ATT AAA ACA CCC ATA CGA GCT ACG AAA ACA 
                P   R   V   A   K   A   S   K   A   K   I   K   T   P   I   R   A   T   K   T  
LdIV1_ZY       CCT CGT GTT GCC AAA GCG TCA AAA GCT AAA ATT AAA ACA CCC ATA CGA GCT ACG AAA ACA 
                P   R   V   A   K   A   S   K   A   K   I   K   T   P   I   R   A   T   K   T  
LdIV1_NJ       CCT CGT GTT GCC AAA GCG TCA AAA GCT AAA ATT AAA ACA CCC ATA CGA GCT ACG AAA ACA 
                P   R   V   A   K   A   S   K   A   K   I   K   T   P   I   R   A   T   K   T  
LdIV1_CT       CCT CGT GTT GCC AAA GCG TCA AAA GCT AAA ATT AAA ACA CCC ATA CGA GCT ACG AAA ACA 
                P   R   V   A   K   A   S   K   A   K   I   K   T   P   I   R   A   T   K   T  
KJ629170.1     CCT CGT GTT GCC AAA GCG TCA AAA GCT AAA ATT AAA ACA CCC ATA CGA GCT ACG AAA ACA 
                                    =           =                   =           =              


[2221..2240]
                L   E   Y   Q   S   A   Q   Q   F   D   V   V   K   Q   R   L   R   N   N   L  
MT753155.1     TTA GAG TAT CAA AGT GCA CAG CAA TTC GAT GTT GTA AAA CAA AGG TTA AGA AAT AAT TTG 
                L   E   Y   Q   S   A   Q   Q   F   D   V   V   K   Q   R   L   R   N   N   L  
MN938851.1     TTA GAG TAT CAA AGT GCA CAG CAA TTC GAT GTT GTA AAA CAA AGG TTA AGA AAT AAT TTG 
                L   E   Y   Q   S   A   Q   Q   F   D   V   V   K   Q   R   L   R   N   N   L  
LdIV1_JGS      TTA GAG TAT CAA AGT GCG CAG CAA TTT GAT GTT GTA AAA CAA AGG TTA AGA AAT AAT TTG 
                L   E   Y   Q   S   A   Q   Q   F   D   V   V   K   Q   R   L   R   N   N   L  
LdIV1_ZY       TTA GAG TAT CAA AGT GCG CAG CAA TTT GAT GTT GTA AAA CAA AGG TTA AGA AAT AAT TTG 
                L   E   Y   Q   S   A   Q   Q   F   D   V   V   K   Q   R   L   R   N   N   L  
LdIV1_NJ       TTA GAG TAT CAA AGT GCG CAG CAA TTT GAT GTT GTA AAA CAA AGG TTA AGA AAT AAT TTG 
                L   E   Y   Q   S   A   Q   Q   F   D   V   V   K   Q   R   L   R   N   N   L  
LdIV1_CT       TTA GAG TAT CAA AGT GCG CAG CAA TTT GAT GTT GTA AAA CAA AGG TTA AGA AAT AAT TTG 
                L   E   Y   Q   G   A   Q   Q   F   D   V   V   K   Q   R   L   R   N   N   L  
KJ629170.1     TTA GAG TAT CAA GGT GCG CAG CAA TTT GAT GTT GTA AAA CAA AGG TTA AGA AAT AAT TTG 
                                ^   =           =                                              


[2241..2260]
                S   S   I   D   V   V   Y   T   D   M   E   G   N   L   K   R   T   R   N   F  
MT753155.1     TCT TCA ATT GAT GTT GTC TAT ACG GAT ATG GAA GGA AAT CTT AAA AGA ACG AGG AAT TTT 
                S   S   I   D   V   V   Y   T   D   V   E   G   N   L   K   R   T   R   N   F  
MN938851.1     TCT TCA ATT GAT GTT GTC TAT ACG GAT GTG GAA GGA AAT CTT AAA AGA ACG AGG AAT TTT 
                S   S   I   D   V   V   Y   T   D   V   E   G   N   M   K   R   T   R   N   F  
LdIV1_JGS      TCT TCG ATT GAT GTT GTC TAT ACG GAT GTG GAA GGA AAT ATG AAA AGA ACG AGA AAT TTT 
                S   S   I   D   V   V   Y   T   D   V   E   G   N   M   K   R   T   R   N   F  
LdIV1_ZY       TCT TCG ATT GAT GTT GTC TAT ACG GAT GTG GAA GGA AAT ATG AAA AGA ACG AGA AAT TTT 
                S   S   I   D   V   V   Y   T   D   V   E   G   N   M   K   R   T   R   N   F  
LdIV1_NJ       TCT TCG ATT GAT GTT GTC TAT ACG GAT GTG GAA GGA AAT ATG AAA AGA ACG AGA AAT TTT 
                S   S   I   D   V   V   Y   T   D   V   E   G   N   M   K   R   T   R   N   F  
LdIV1_CT       TCT TCG ATT GAT GTT GTC TAT ACG GAT GTG GAA GGA AAT ATG AAA AGA ACG AGA AAT TTT 
                S   S   I   D   V   V   Y   T   D   V   E   G   N   M   K   R   T   R   N   F  
KJ629170.1     TCT TCG ATT GAT GTT GTT TAT ACG GAT GTG GAA GGA AAT ATG AAA AGA ACG AGA AAT TTT 
                    =               =               ^               ^               =          


[2261..2280]
                G   L   M   L   K   D   Q   Q   M   L   I   Q   K   H   Y   Y   D   F   W   K  
MT753155.1     GGC CTG ATG TTA AAG GAT CAA CAA ATG CTT ATA CAG AAA CAT TAT TAT GAC TTT TGG AAA 
                G   L   M   L   K   D   Q   Q   M   L   I   Q   K   H   Y   Y   D   F   W   K  
MN938851.1     GGC TTG ATG TTA AAG GAT CAA CAA ATG CTT ATA CAG AAA CAT TAT TAT GAC TTT TGG AAA 
                G   L   M   L   K   D   Q   Q   M   L   I   Q   K   H   Y   Y   D   F   W   K  
LdIV1_JGS      GGC CTG ATG TTG AAG GAT CAA CAA ATG TTA ATA CAG AAA CAT TAT TAT GAC TTT TGG AAA 
                G   L   M   L   K   D   Q   Q   M   L   I   Q   K   H   Y   Y   D   F   W   K  
LdIV1_ZY       GGC CTG ATG TTG AAG GAT CAA CAA ATG TTA ATA CAG AAA CAT TAT TAT GAC TTT TGG AAA 
                G   L   M   L   K   D   Q   Q   M   L   I   Q   K   H   Y   Y   D   F   W   K  
LdIV1_NJ       GGC CTG ATG TTG AAG GAT CAA CAA ATG TTA ATA CAG AAA CAT TAT TAT GAT TTT TGG AAA 
                G   L   M   L   K   D   Q   Q   M   L   I   Q   K   H   Y   Y   D   F   W   K  
LdIV1_CT       GGC CTG ATG TTG AAG GAT CAA CAA ATG TTA ATA CAG AAA CAT TAT TAT GAC TTT TGG AAA 
                G   L   M   L   K   D   Q   Q   M   L   I   Q   K   H   Y   Y   D   F   W   K  
KJ629170.1     GGC CTG ATG TTG AAG GAT CAA CAA ATG TTA ATA CAG AAA CAT TAT TAT GAC TTT TGG AAA 
                    =       =                       =                           =              


[2281..2300]
                R   L   D   L   T   A   K   F   Y   F   Y   N   N   N   I   K   S   H   A   P  
MT753155.1     CGG TTA GAT CTT ACT GCT AAA TTT TAT TTC TAT AAT AAT AAT ATT AAG AGT CAT GCT CCA 
                R   L   D   L   T   A   K   F   Y   F   Y   N   N   N   I   K   S   H   A   P  
MN938851.1     CGG TTA GAT CTT ACT GCT AAA TTT TAT TTC TAT AAT AAT AAT ATT AAG AGT CAT GCT CCA 
                R   L   D   L   T   A   K   F   Y   F   Y   N   N   N   I   K   S   H   A   P  
LdIV1_JGS      CGA TTA GAT CTT ACT GCT AAA TTT TAT TTC TAT AAT AAC AAT ATT AAG AGT CAT GCT CCA 
                R   L   D   L   T   A   K   F   Y   F   Y   N   N   N   I   K   S   H   A   P  
LdIV1_ZY       CGA TTA GAT CTT ACT GCT AAA TTT TAT TTC TAT AAT AAC AAT ATT AAG AGT CAT GCT CCA 
                R   L   D   L   T   A   K   F   Y   F   Y   N   N   N   I   K   S   H   A   P  
LdIV1_NJ       CGA TTA GAT CTT ACT GCT AAA TTT TAT TTC TAT AAT AAC AAT ATT AAG AGT CAT GCT CCG 
                R   L   D   L   T   A   K   F   Y   F   Y   N   N   N   I   K   S   H   A   P  
LdIV1_CT       CGA TTA GAT CTT ACT GCT AAA TTT TAT TTC TAT AAT AAC AAT ATT AAA AGT CAT GCT CCG 
                R   L   D   L   T   A   K   F   Y   F   Y   N   N   N   I   K   S   H   A   P  
KJ629170.1     CGA TTA GAT CTT ACT GCT AAA TTT TAT TTC TAT AAT AAC AAT ATT AAG AGT CAT GCT CCG 
                =                                               =           =               =  


[2301..2320]
                D   G   I   L   L   T   N   F   F   D   L   D   I   D   W   F   M   T   P   D  
MT753155.1     GAC GGT ATT TTA TTG ACG AAT TTC TTC GAT TTA GAT ATT GAT TGG TTC ATG ACT CCT GAT 
                D   G   I   L   L   T   N   F   F   D   L   D   I   D   W   F   M   T   P   D  
MN938851.1     GAC GGT ATT TTA TTG ACG AAT TTC TTC GAT TTA GAT ATT GAT TGG TTC ATG ACT CCT GAT 
                D   G   I   L   L   T   N   F   F   D   L   D   I   D   W   F   M   T   P   D  
LdIV1_JGS      GAC GGT ATT TTA TTG ACG AAT TTC TTC GAT TTA GAT ATT GAT TGG TTT ATG ACT CCT GAT 
                D   G   I   L   L   T   N   F   F   D   L   D   I   D   W   F   M   T   P   D  
LdIV1_ZY       GAC GGT ATT TTA TTG ACG AAT TTC TTC GAT TTA GAT ATT GAT TGG TTT ATG ACT CCT GAT 
                D   G   I   L   L   T   N   F   F   D   L   D   I   D   W   F   M   T   P   D  
LdIV1_NJ       GAC GGT ATT TTA TTG ACG AAT TTC TTC GAT TTA GAT ATT GAT TGG TTT ATG ACT CCT GAT 
                D   G   I   L   L   T   N   F   F   D   L   D   I   D   W   F   M   T   P   D  
LdIV1_CT       GAC GGT ATT TTA TTG ACG AAT TTC TTC GAT TTA GAT ATT GAT TGG TTT ATG ACT CCT GAT 
                D   G   I   L   L   T   N   F   F   D   L   D   V   D   W   F   M   T   P   D  
KJ629170.1     GAC GGT ATT TTA TTA ACG AAT TTC TTT GAT TTA GAT GTT GAT TGG TTT ATG ACT CCT GAT 
                                =               =               ^           =                  


[2321..2340]
                K   D   I   F   D   S   N   F   G   I   L   H   L   P   K   I   V   P   A   Y  
MT753155.1     AAA GAT ATT TTT GAT AGT AAT TTT GGA ATA TTG CAT CTT CCT AAA ATT GTT CCT GCT TAT 
                K   D   I   F   D   S   N   F   G   I   L   H   L   P   K   I   V   P   A   Y  
MN938851.1     AAA GAT ATT TTT GAT AGT AAT TTT GGA ATA TTG CAT CTT CCT AAA ATT GTT CCT GCT TAT 
                K   D   I   F   D   S   N   F   G   I   L   H   L   P   K   I   V   P   A   Y  
LdIV1_JGS      AAA GAT ATC TTT GAT AGT AAC TTC GGA ATT TTG CAT CTT CCT AAA ATT GTT CCT GCT TAT 
                K   D   I   F   D   S   N   F   G   I   L   H   L   P   K   I   V   P   A   Y  
LdIV1_ZY       AAA GAT ATC TTT GAT AGT AAC TTC GGA ATT TTG CAT CTT CCT AAA ATT GTT CCT GCT TAT 
                K   D   I   F   D   S   N   F   G   I   L   H   L   P   K   I   V   P   A   Y  
LdIV1_NJ       AAA GAT ATT TTT GAT AGT AAC TTC GGA ATA TTG CAT CTT CCT AAA ATT GTT CCT GCT TAT 
                K   D   I   F   D   S   N   F   G   I   L   H   L   P   K   I   V   P   A   Y  
LdIV1_CT       AAA GAT ATT TTT GAT AGT AAC TTC GGA ATA TTG CAT CTT CCT AAA ATT GTT CCT GCT TAT 
                Q   D   I   F   D   S   N   F   G   I   L   H   L   P   K   I   V   P   A   Y  
KJ629170.1     CAA GAT ATT TTT GAT AGT AAC TTC GGA ATA TTG CAT CTT CCT AAA ATT GTT CCT GCT TAT 
                ^       =               =   =       =                                          


[2341..2360]
                K   D   L   T   K   F   I   A   K   S   T   E   H   Q   Y   I   K   F   D   E  
MT753155.1     AAA GAT TTA ACA AAG TTT ATA GCT AAA TCA ACT GAA CAT CAA TAT ATT AAA TTT GAC GAA 
                K   D   L   T   K   F   I   A   K   S   T   E   H   Q   Y   I   K   F   D   E  
MN938851.1     AAA GAT TTA ACA AAG TTT ATA GCT AAA TCA ACT GAA CAT CAA TAT ATT AAA TTT GAC GAA 
                K   D   L   T   K   F   I   A   K   S   T   E   H   Q   Y   I   K   F   D   E  
LdIV1_JGS      AAA GAT TTA ACA AAG TTT ATA GCT AAA TCA ACT GAA CAC CAA TAT ATT AAA TTT GAC GAA 
                K   D   L   T   K   F   I   A   K   S   T   E   H   Q   Y   I   K   F   D   E  
LdIV1_ZY       AAA GAT TTA ACA AAG TTT ATA GCT AAA TCA ACT GAA CAC CAA TAT ATT AAA TTT GAC GAA 
                K   D   L   T   K   F   I   A   K   S   T   E   H   Q   Y   I   K   F   D   E  
LdIV1_NJ       AAA GAT TTA ACA AAG TTT ATA GCT AAA TCA ACT GAA CAT CAA TAT ATT AAA TTT GAC GAA 
                K   D   L   T   K   F   I   A   K   S   T   E   H   Q   Y   I   K   F   D   E  
LdIV1_CT       AAA GAT TTA ACA AAG TTT ATA GCT AAA TCA ACT GAA CAT CAA TAT ATT AAA TTT GAC GAA 
                K   D   L   T   K   F   I   A   K   S   T   E   H   Q   Y   I   K   F   D   E  
KJ629170.1     AAA GAT TTA ACA AAG TTT ATA GCT AAG TCA ACT GAA CAT CAA TAT ATT AAA TTT GAC GAA 
                                                =               =                              


[2361..2380]
                C   Y   L   Y   S   S   L   S   G   E   S   M   H   C   V   M   N   I   E   Y  
MT753155.1     TGT TAT CTT TAT TCA AGT CTG TCT GGA GAG AGT ATG CAT TGT GTG ATG AAT ATT GAG TAT 
                C   Y   L   Y   S   S   L   S   G   E   S   M   H   C   V   M   N   I   E   Y  
MN938851.1     TGT TAT CTT TAT TCA AGT CTG TCT GGA GAG AGT ATG CAT TGT GTG ATG AAT ATT GAG TAT 
                C   Y   L   Y   S   S   L   S   G   E   S   M   H   C   V   M   N   I   E   Y  
LdIV1_JGS      TGT TAC CTT TAT TCA AGT CTG TCT GGA GAG AGT ATG CAT TGT GTT ATG AAC ATC GAG TAT 
                C   Y   L   Y   S   S   L   S   G   E   S   M   H   C   V   M   N   I   E   Y  
LdIV1_ZY       TGT TAC CTT TAT TCA AGT CTG TCT GGA GAG AGT ATG CAT TGT GTT ATG AAC ATC GAG TAT 
                C   Y   L   Y   S   S   L   S   G   E   S   M   H   C   V   M   N   I   E   Y  
LdIV1_NJ       TGT TAT CTT TAT TCA AGT TTG TCT GGA GAG AGT ATG CAT TGC GTG ATG AAT ATC GAG TAT 
                C   Y   L   Y   S   S   L   S   G   E   S   M   H   C   V   M   N   I   E   Y  
LdIV1_CT       TGT TAT CTT TAT TCA AGT TTG TCT GGA GAG AGT ATG CAT TGC GTG ATG AAT ATC GAG TAT 
                C   Y   L   Y   S   S   L   S   G   E   S   M   H   C   V   M   N   I   E   Y  
KJ629170.1     TGT TAT CTT TAT TCA AGT TTG TCT GGA GAG AGT ATG CAT TGC GTG ATG AAT ATC GAG TAT 
                    =                   =                           =   =       =   =          


[2381..2400]
                N   K   E   V   T   D   A   N   G   W   L   R   L   D   E   C   Y   S   Y   R  
MT753155.1     AAT AAA GAG GTC ACT GAC GCA AAT GGA TGG CTT CGG TTG GAT GAA TGT TAT TCG TAT AGA 
                N   K   E   V   T   D   A   N   G   W   L   R   L   D   E   C   Y   S   Y   R  
MN938851.1     AAT AAA GAG GTC ACT GAC GCA AAT GGA TGG CTT CGG TTG GAT GAA TGT TAT TCG TAT AGA 
                N   K   E   V   T   D   A   N   G   W   L   R   L   D   E   C   Y   S   Y   R  
LdIV1_JGS      AAT AAA GAA GTC ACT GAT GCA AAT GGG TGG CTT CGA TTG GAT GAA TGT TAT TCG TAT AGA 
                N   K   E   V   T   D   A   N   G   W   L   R   L   D   E   C   Y   S   Y   R  
LdIV1_ZY       AAT AAA GAA GTC ACT GAT GCA AAT GGG TGG CTT CGA TTG GAT GAA TGT TAT TCG TAT AGA 
                N   K   E   V   T   D   A   N   G   W   L   R   L   D   E   C   Y   S   Y   R  
LdIV1_NJ       AAT AAA GAA GTC ACT GAT GCA AAT GGG TGG CTT CGG TTG GAT GAA TGT TAT TCG TAT AGA 
                N   K   E   V   T   D   A   N   G   W   L   R   L   D   E   C   Y   S   Y   R  
LdIV1_CT       AAT AAA GAA GTC ACT GAT GCA AAT GGG TGG CTT CGG TTG GAT GAA TGT TAT TCG TAT AGA 
                N   K   E   V   T   D   A   N   G   W   L   R   L   D   E   C   Y   S   Y   K  
KJ629170.1     AAT AAA GAA GTC ACT GAC GCA AAT GGG TGG CTT CGG TTG GAT GAA TGT TAT TCG TAT AAA 
                        =           =           =           =                               ^  


[2401..2420]
                Y   T   G   V   G   L   C   G   S   A   L   L   C   S   T   L   E   R   P   I  
MT753155.1     TAT ACT GGA GTA GGC TTA TGT GGT AGT GCA CTG TTG TGT AGT ACT TTA GAA AGA CCA ATT 
                Y   T   G   V   G   L   C   G   S   A   L   L   C   S   T   L   E   R   P   I  
MN938851.1     TAT ACT GGA GTA GGC TTA TGT GGT AGT GCA CTG TTG TGT AGT ACT TTA GAA AGA CCA ATT 
                Y   T   G   I   G   L   C   G   S   A   L   L   C   S   T   L   E   R   P   I  
LdIV1_JGS      TAT ACT GGA ATA GGC TTA TGT GGT AGT GCA CTG TTG TGT AGT ACT TTA GAA AGA CCA ATT 
                Y   T   G   I   G   L   C   G   S   A   L   L   C   S   T   L   E   R   P   I  
LdIV1_ZY       TAT ACT GGA ATA GGC TTA TGT GGT AGT GCA CTG TTG TGT AGT ACT TTA GAA AGA CCA ATT 
                Y   T   G   V   G   L   C   G   S   A   L   L   C   S   T   L   E   R   P   I  
LdIV1_NJ       TAT ACT GGA GTA GGC TTA TGT GGT AGT GCA CTA TTG TGT AGT ACT TTA GAA AGA CCA ATT 
                Y   T   G   V   G   L   C   G   S   A   L   L   C   S   T   L   E   R   P   I  
LdIV1_CT       TAT ACT GGA GTA GGC TTA TGT GGT AGT GCA CTA TTG TGT AGT ACT TTA GAA AGA CCA ATT 
                Y   T   G   V   G   L   C   G   S   A   L   L   C   S   T   L   E   R   P   I  
KJ629170.1     TAT ACT GGA GTA GGC TTA TGT GGT AGT GCA CTA TTG TGT AGT ACT TTA GAA AGA CCA ATT 
                            ^                           =                                      


[2421..2440]
                V   G   V   H   F   A   G   T   S   T   Y   G   Y   A   E   P   L   C   Y   E  
MT753155.1     GTT GGT GTG CAC TTT GCT GGT ACT TCT ACG TAT GGT TAT GCC GAA CCG TTG TGT TAT GAA 
                V   G   V   H   F   A   G   T   S   T   Y   G   Y   A   E   P   L   C   Y   E  
MN938851.1     GTT GGT GTG CAC TTT GCT GGT ACT TCT ACG TAT GGT TAT GCC GAA CCG TTG TGT TAT GAA 
                V   G   V   H   F   A   G   T   S   T   Y   G   Y   A   E   P   L   C   Y   E  
LdIV1_JGS      GTT GGT GTC CAC TTT GCC GGT ACT TCC ACG TAT GGC TAT GCT GAA CCG TTG TGT TAT GAA 
                V   G   V   H   F   A   G   T   S   T   Y   G   Y   A   E   P   L   C   Y   E  
LdIV1_ZY       GTT GGT GTC CAC TTT GCC GGT ACT TCC ACG TAT GGC TAT GCT GAA CCG TTG TGT TAT GAA 
                V   G   V   H   F   A   G   T   S   T   Y   G   Y   A   E   P   L   C   Y   E  
LdIV1_NJ       GTT GGT GTG CAC TTT GCT GGT ACT TCT ACG TAT GGT TAT GCC GAA CCG TTG TGT TAT GAA 
                V   G   V   H   F   A   G   T   S   T   Y   G   Y   A   E   P   L   C   Y   E  
LdIV1_CT       GTT GGT GTG CAC TTT GCT GGT ACT TCT ACG TAT GGT TAT GCC GAA CCG TTG TGT TAT GAA 
                I   G   V   H   F   A   G   T   S   T   Y   G   Y   A   E   P   L   C   Y   E  
KJ629170.1     ATT GGT GTG CAC TTT GCT GGT ACT TCT ACG TAT GGT TAT GCC GAA CCG TTG TGT TAT GAA 
                ^       =           =           =           =       =                          


[2441..2460]
                S   F   N   Q   I   D   V   K   H   Y   D   Y   D   L   C   D   L   R   L   D  
MT753155.1     TCA TTT AAT CAA ATC GAT GTG AAA CAT TAT GAT TAT GAT TTG TGT GAT TTG CGA TTG GAT 
                S   F   N   Q   I   D   V   K   H   Y   D   Y   D   L   C   D   L   R   L   D  
MN938851.1     TCA TTT AAT CAA ATC GAT GTG AAA CAT TAT GAT TAT GAT TTG TGT GAT TTG CGA TTG GAT 
                S   F   N   Q   I   D   V   K   H   Y   D   Y   D   L   C   D   L   R   L   D  
LdIV1_JGS      TCG TTT AAT CAA ATC GAC GTG AAA CAT TAT GAT TAT GAT TTG TGT GAT TTG CGA TTG GAT 
                S   F   N   Q   I   D   V   K   H   Y   D   Y   D   L   C   D   L   R   L   D  
LdIV1_ZY       TCG TTT AAT CAA ATC GAC GTG AAA CAT TAT GAT TAT GAT TTG TGT GAT TTG CGA TTG GAT 
                S   F   N   Q   I   D   V   K   H   Y   D   Y   D   L   C   D   L   R   L   D  
LdIV1_NJ       TCA TTT AAT CAA ATT GAC GTG AAA CAT TAT GAT TAT GAT TTG TGT GAT TTG CGA TTG GAT 
                S   F   N   Q   I   D   V   K   H   Y   D   Y   D   L   C   D   L   R   L   D  
LdIV1_CT       TCA TTT AAT CAA ATT GAC GTG AAA CAT TAT GAT TAT GAT TTG TGT GAT TTG CGA TTG GAT 
                S   F   N   Q   I   D   V   K   H   Y   D   Y   D   L   C   D   L   R   L   D  
KJ629170.1     TCA TTT AAT CAA ATT GAC GTA AAA CAT TAT GAT TAT GAT TTG TGT GAT TTG CGA TTG GAT 
                =               =   =   =                                                      


[2461..2480]
                G   S   K   E   K   I   Q   F   D   T   L   L   Y   P   Q   G   T   V   P   D  
MT753155.1     GGT TCT AAG GAA AAA ATT CAG TTT GAT ACC CTA TTA TAT CCG CAG GGT ACT GTG CCA GAT 
                G   S   K   E   K   I   Q   F   D   T   L   L   Y   P   Q   G   T   V   P   D  
MN938851.1     GGT TCT AAG GAA AAA ATT CAG TTT GAT ACC CTA TTA TAT CCG CAG GGT ACT GTG CCA GAT 
                G   S   K   E   K   I   Q   F   D   T   L   L   Y   P   Q   G   T   V   P   D  
LdIV1_JGS      GGT TCT AAG GAA AAA ATT CAA TTT GAT ACC CTA TTA TAT CCG CAG GGT ACT GTG CCA GAT 
                G   S   K   E   K   I   Q   F   D   T   L   L   Y   P   Q   G   T   V   P   D  
LdIV1_ZY       GGT TCT AAG GAA AAA ATT CAA TTT GAT ACC CTA TTA TAT CCG CAG GGT ACT GTG CCA GAT 
                G   S   K   E   K   I   Q   F   D   T   L   L   Y   P   Q   G   T   V   P   D  
LdIV1_NJ       GGT TCT AAG GAA AAA ATT CAA TTT GAT ACC CTA TTA TAT CCG CAG GGT ACT GTG CCA GAT 
                G   S   K   E   K   I   Q   F   D   T   L   L   Y   P   Q   G   T   V   P   D  
LdIV1_CT       GGT TCT AAG GAA AAA ATT CAA TTT GAT ACC CTA TTA TAT CCG CAG GGT ACT GTG CCA GAT 
                G   S   K   E   K   I   Q   F   D   T   L   L   Y   P   Q   G   T   V   P   D  
KJ629170.1     GGT TCT AAG GAA AAA ATT CAA TTT GAT ACC CTA TTA TAT CCG CAG GGT ACT GTG CCA GAC 
                                        =                                                   =  


[2481..2500]
                V   Y   S   H   H   Q   G   C   V   S   Q   Y   I   P   S   L   V   H   G   V  
MT753155.1     GTG TAT AGC CAT CAT CAA GGT TGT GTT AGT CAG TAT ATC CCA AGT TTG GTT CAT GGA GTG 
                V   Y   S   H   H   Q   G   C   V   S   Q   Y   I   P   S   L   V   H   G   V  
MN938851.1     GTG TAT AGC CAT CAT CAA GGT TGT GTT AGT CAG TAT ATC CCA AGT TTG GTT CAT GGA GTG 
                V   Y   S   H   H   Q   G   C   V   S   Q   Y   I   P   S   L   V   H   G   V  
LdIV1_JGS      GTG TAT AGC CAC CAT CAA GGT TGT GTT AGT CAG TAT ATC CCA AGT TTG GTT CAT GGA GTG 
                V   Y   S   H   H   Q   G   C   V   S   Q   Y   I   P   S   L   V   H   G   V  
LdIV1_ZY       GTG TAT AGC CAC CAT CAA GGT TGT GTT AGT CAG TAT ATC CCA AGT TTG GTT CAT GGA GTG 
                V   Y   S   H   H   Q   G   C   V   S   Q   Y   I   P   S   L   V   H   G   V  
LdIV1_NJ       GTG TAT AGC CAT CAT CAA GGT TGT GTT AGT CAA TAT ATC CCA AGT TTG GTT CAT GGA GTG 
                V   Y   S   H   H   Q   G   C   V   S   Q   Y   I   P   S   L   V   H   G   V  
LdIV1_CT       GTG TAT AGC CAT CAT CAA GGT TGT GTT AGT CAA TAT ATC CCA AGT TTG GTT CAT GGA GTG 
                V   Y   S   H   H   Q   G   C   V   S   Q   Y   I   P   S   L   V   H   G   V  
KJ629170.1     GTG TAT AGC CAT CAT CAA GGT TGT GTT AGT CAA TAT ATC CCA AGT TTG GTT CAT GGA GTG 
                            =                           =                                      


[2501..2520]
                Y   E   V   D   T   E   P   N   P   L   S   P   R   D   E   R   L   P   P   G  
MT753155.1     TAT GAA GTT GAT ACT GAA CCA AAC CCA TTG TCA CCA CGA GAT GAA CGA TTG CCA CCC GGA 
                Y   E   V   D   T   E   P   N   P   L   S   P   R   D   E   R   L   P   P   G  
MN938851.1     TAT GAA GTT GAT ACT GAA CCA AAC CCA TTG TCA CCA CGA GAT GAA CGA TTG CCA CCC GGA 
                Y   E   V   D   T   E   P   N   P   L   S   P   R   D   E   R   L   P   P   G  
LdIV1_JGS      TAT GAA GTT GAT ACT GAA CCA AAC CCA TTG TCA CCA CGA GAT GAA CGA TTG CCA CCC GGA 
                Y   E   V   D   T   E   P   N   P   L   S   P   R   D   E   R   L   P   P   G  
LdIV1_ZY       TAT GAA GTT GAT ACT GAA CCA AAC CCA TTG TCA CCA CGA GAT GAA CGA TTG CCA CCC GGA 
                Y   E   V   D   T   E   P   N   P   L   S   P   R   D   E   R   L   P   P   G  
LdIV1_NJ       TAT GAA GTT GAT ACT GAA CCG AAC CCA TTA TCA CCA CGA GAT GAA CGA TTG CCA CCC GGA 
                Y   E   V   D   T   E   P   N   P   L   S   P   R   D   E   R   L   P   P   G  
LdIV1_CT       TAT GAA GTT GAT ACT GAA CCG AAC CCA TTA TCA CCA CGA GAT GAA CGA TTG CCA CCC GGA 
                Y   E   V   D   T   E   P   N   P   L   S   P   R   D   E   R   L   P   P   G  
KJ629170.1     TAT GAA GTT GAT ACT GAA CCG AAC CCA TTA TCA CCA CGA GAT GAA CGA TTG CCA CCC GGA 
                                        =           =                                          


[2521..2540]
                N   P   P   L   Q   R   G   V   E   H   M   G   K   P   P   L   D   F   P   S  
MT753155.1     AAC CCA CCC TTA CAG CGA GGA GTC GAA CAT ATG GGT AAA CCT CCT TTA GAT TTT CCA AGT 
                N   P   P   L   Q   R   G   V   E   H   M   G   K   P   P   L   D   F   P   S  
MN938851.1     AAC CCA CCC TTA CAG CGA GGA GTC GAA CAT ATG GGT AAA CCT CCT TTA GAT TTT CCA AGT 
                N   P   P   L   Q   R   G   V   E   H   M   G   K   P   P   L   D   F   P   S  
LdIV1_JGS      AAC CCA CCC TTA CAG CGA GGA GTC GAA CAT ATG GGC AAA CCT CCT TTA GAT TTT CCA AGT 
                N   P   P   L   Q   R   G   V   E   H   M   G   K   P   P   L   D   F   P   S  
LdIV1_ZY       AAC CCA CCC TTA CAG CGA GGA GTC GAA CAT ATG GGC AAA CCT CCT TTA GAT TTT CCA AGT 
                N   P   P   L   Q   R   G   V   E   H   M   G   K   P   P   L   D   F   P   S  
LdIV1_NJ       AAT CCA CCC TTA CAG CGA GGA GTT GAA CAT ATG GGT AAA CCT CCT TTA GAT TTT CCA AGT 
                N   P   P   L   Q   R   G   V   E   H   M   G   K   P   P   L   D   F   P   S  
LdIV1_CT       AAT CCA CCC TTA CAG CGA GGA GTT GAA CAT ATG GGT AAA CCT CCT TTA GAT TTT CCA AGT 
                N   P   P   L   Q   R   G   V   E   H   M   G   K   P   P   L   D   F   P   N  
KJ629170.1     AAT CCA CCC TTA CAG CGA GGA GTT GAA CAT ATG GGT AAA CCT CCT TTA GAT TTT CCA AAT 
                =                           =               =                               ^  


[2541..2560]
                D   L   L   K   P   A   A   R   D   L   E   D   V   I   L   R   T   V   K   P  
MT753155.1     GAT TTA TTA AAA CCA GCA GCA AGA GAT TTG GAG GAT GTA ATT TTG CGT ACC GTT AAA CCT 
                D   L   L   K   P   A   A   R   D   L   E   D   V   I   L   R   T   V   K   P  
MN938851.1     GAT TTA TTA AAA CCA GCA GCA AGA GAT TTG GAG GAT GTA ATT TTG CGT ACC GTT AAA CCT 
                D   L   L   K   P   A   A   R   D   L   E   D   V   I   L   R   T   V   K   P  
LdIV1_JGS      GAT TTA TTA AAA CCA GCA GCA AGA GAT TTG GAG GAT GTA ATT TTG CGT ACT GTT AAA CCT 
                D   L   L   K   P   A   A   R   D   L   E   D   V   I   L   R   T   V   K   P  
LdIV1_ZY       GAT TTA TTA AAA CCA GCA GCA AGA GAT TTG GAG GAT GTA ATT TTG CGT ACT GTT AAA CCT 
                D   L   L   K   P   A   A   R   D   L   E   D   V   I   L   R   T   V   K   P  
LdIV1_NJ       GAT TTA TTA AAA CCA GCA GCA AGA GAT TTG GAA GAT GTA ATT TTG CGT ACT GTC AAA CCT 
                D   L   L   K   P   A   A   R   D   L   E   D   V   I   L   R   T   V   K   P  
LdIV1_CT       GAT TTA TTA AAA CCA GCA GCA AGA GAT TTG GAA GAT GTA ATT TTG CGT ACT GTC AAA CCT 
                D   L   L   R   P   A   A   R   D   L   E   D   V   I   L   R   T   V   K   P  
KJ629170.1     GAT TTA TTA AGA CCA GCA GCA AGA GAT TTG GAA GAT GTG ATT TTG CGT ACT GTC AAA CCT 
                            ^                           =       =               =   =          


[2561..2580]
                V   R   L   V   M   D   K   I   S   L   Q   D   A   I   C   G   N   V   N   V  
MT753155.1     GTT CGG CTT GTT ATG GAT AAA ATA TCG CTA CAA GAT GCA ATT TGT GGA AAT GTT AAT GTT 
                V   R   L   V   M   D   K   I   S   L   Q   D   A   I   C   G   N   V   N   V  
MN938851.1     GTT CGG CTT GTT ATG GAT AAA ATA TCG CTA CAA GAT GCA ATT TGT GGA AAT GTT AAT GTT 
                V   R   L   V   M   D   K   I   S   L   Q   D   A   I   C   G   N   V   N   V  
LdIV1_JGS      GTT CGG CTT GTT ATG GAT AAA ATA TCG CTA CAA GAT GCA ATT TGT GGA AAT GTT AAT GTT 
                V   R   L   V   M   D   K   I   S   L   Q   D   A   I   C   G   N   V   N   V  
LdIV1_ZY       GTT CGG CTT GTT ATG GAT AAA ATA TCG CTA CAA GAT GCA ATT TGT GGA AAT GTT AAT GTT 
                V   R   L   V   M   D   K   I   S   L   Q   D   A   I   C   G   N   V   N   V  
LdIV1_NJ       GTT CGG CTT GTT ATG GAC AAA ATA TCG CTA CAA GAT GCA ATT TGT GGA AAT GTC AAT GTT 
                V   R   L   V   M   D   K   I   S   L   Q   D   A   I   C   G   N   V   N   V  
LdIV1_CT       GTT CGG CTT GTT ATG GAC AAA ATA TCG CTA CAA GAT GCA ATT TGT GGA AAT GTC AAT GTT 
                V   R   L   V   M   D   K   I   S   L   Q   D   A   I   C   G   N   V   N   V  
KJ629170.1     GTT CGG CTT GTT ATG GAC AAA ATA TCG CTA CAA GAT GCA ATT TGT GGA AAT GTC AAT GTT 
                                    =                                               =          


[2581..2600]
                K   G   F   E   P   L   E   W   S   S   S   E   G   F   P   L   R   R   L   R  
MT753155.1     AAA GGA TTT GAA CCG TTA GAG TGG AGC TCA AGT GAG GGT TTT CCT CTT AGA CGA CTT CGT 
                K   G   F   E   P   L   E   W   S   S   S   E   G   F   P   L   R   R   L   R  
MN938851.1     AAA GGA TTT GAA CCA TTA GAG TGG AGC TCA AGT GAG GGT TTT CCT CTT AGA CGA CTT CGT 
                K   G   F   E   P   L   E   W   S   S   S   E   G   F   P   L   R   R   L   R  
LdIV1_JGS      AAA GGA TTT GAA CCG TTA GAG TGG AGC TCA AGT GAG GGT TTT CCT CTT AGA CGA CTT CGT 
                K   G   F   E   P   L   E   W   S   S   S   E   G   F   P   L   R   R   L   R  
LdIV1_ZY       AAA GGA TTT GAA CCG TTA GAG TGG AGC TCA AGT GAG GGT TTT CCT CTT AGA CGA CTT CGT 
                K   G   F   E   P   L   E   W   S   S   S   E   G   F   P   L   R   R   L   R  
LdIV1_NJ       AAA GGA TTT GAA CCG TTA GAG TGG AGC TCA AGT GAG GGT TTT CCT CTT AGA CGA CTT CGT 
                K   G   F   E   P   L   E   W   S   S   S   E   G   F   P   L   R   R   L   R  
LdIV1_CT       AAA GGA TTT GAA CCG TTG GAG TGG AGC TCA AGT GAG GGT TTT CCT CTT AGA CGA CTT CGT 
                K   G   F   E   P   L   E   W   S   S   S   E   G   F   P   L   R   R   L   R  
KJ629170.1     AAA GGA TTT GAA CCG TTG GAG TGG AGC TCA AGT GAG GGT TTT CCT CTT AGA CGA CTT CGT 
                                =   =                                                          


[2601..2620]
                P   S   G   V   K   G   K   K   W   L   F   D   L   E   E   T   P   T   G   Y  
MT753155.1     CCA TCT GGA GTC AAA GGA AAA AAA TGG TTG TTT GAT CTT GAA GAA ACT CCT ACA GGT TAT 
                P   S   G   V   K   G   K   K   W   L   F   D   L   E   E   T   P   T   G   Y  
MN938851.1     CCA TCT GGA GTC AAA GGG AAA AAA TGG TTG TTT GAT CTT GAA GAA ACT CCT ACA GGT TAT 
                P   S   G   V   K   G   K   K   W   L   F   D   L   E   E   T   P   T   G   Y  
LdIV1_JGS      CCA TCT GGA GTC AAA GGG AAA AAA TGG TTG TTT GAT CTT GAA GAA ACT CCT ACA GGT TAT 
                P   S   G   V   K   G   K   K   W   L   F   D   L   E   E   T   P   T   G   Y  
LdIV1_ZY       CCA TCT GGA GTC AAA GGG AAA AAA TGG TTG TTT GAT CTT GAA GAA ACT CCT ACA GGT TAT 
                P   S   G   V   K   G   K   K   W   L   F   D   L   E   E   T   P   T   G   Y  
LdIV1_NJ       CCA TCT GGA GTC AAA GGG AAA AAA TGG TTG TTT GAT CTT GAA GAA ACT CCT ACA GGT TAT 
                P   S   G   V   K   G   K   K   W   L   F   D   L   E   E   T   P   T   G   Y  
LdIV1_CT       CCA TCT GGA GTC AAA GGG AAA AAA TGG TTG TTT GAT CTT GAA GAA ACT CCT ACA GGT TAT 
                P   S   G   V   K   G   K   K   W   L   F   D   L   E   E   T   P   T   G   Y  
KJ629170.1     CCA TCT GGA GTC AAA GGG AAA AAG TGG TTG TTT GAT CTT GAA GAA ACT CCT ACA GGT TAT 
                                    =       =                                                  


[2621..2640]
                V   L   K   G   M   H   G   E   L   K   R   Q   L   S   I   C   D   A   L   R  
MT753155.1     GTC TTA AAA GGA ATG CAT GGT GAG TTA AAA CGT CAG TTA TCA ATC TGT GAC GCC TTG AGA 
                V   L   R   G   M   H   G   E   L   K   R   Q   L   S   I   C   D   A   L   R  
MN938851.1     GTC TTA AGA GGA ATG CAT GGT GAG TTA AAA CGT CAG TTA TCA ATC TGT GAC GCC TTG AGA 
                V   L   K   G   M   H   G   E   L   K   R   Q   L   S   I   C   D   A   L   R  
LdIV1_JGS      GTC TTA AAA GGA ATG CAT GGT GAG TTA AAA CGT CAG TTA TCG ATC TGC GAC GCC TTG AGA 
                V   L   K   G   M   H   G   E   L   K   R   Q   L   S   I   C   D   A   L   R  
LdIV1_ZY       GTC TTA AAA GGA ATG CAT GGT GAG TTA AAA CGT CAG TTA TCG ATC TGC GAC GCC TTG AGA 
                V   L   K   G   M   H   G   E   L   K   R   Q   L   S   I   C   D   A   L   R  
LdIV1_NJ       GTC TTA AAA GGA ATG CAT GGC GAG TTA AAA CGT CAG CTA TCA ATT TGC GAC GCC TTG AGA 
                V   L   K   G   M   H   G   E   L   K   R   Q   L   S   I   C   D   A   L   R  
LdIV1_CT       GTC TTA AAA GGA ATG CAT GGT GAG TTA AAA CGT CAG TTA TCA ATT TGC GAC GCC TTG AGA 
                V   L   K   G   M   H   G   E   L   K   R   Q   L   S   I   C   D   A   L   R  
KJ629170.1     GTC TTA AAA GGA ATG CAT GGT GAG TTA AAG CGT CAG TTA TCA ATT TGC GAC GCC TTG AGA 
                        ^               =           =           =   =   =   =                  


[2641..2660]
                K   D   S   I   R   C   P   T   I   F   V   D   C   L   K   D   T   C   I   D  
MT753155.1     AAA GAT AGT ATT CGA TGC CCA ACC ATT TTC GTT GAT TGT TTG AAA GAT ACG TGT ATT GAT 
                K   D   S   I   R   C   P   T   I   F   V   D   C   L   K   D   T   C   I   D  
MN938851.1     AAA GAT AGT ATT CGA TGC CCA ACC ATT TTC GTT GAT TGT TTG AAA GAT ACG TGT ATT GAT 
                K   D   S   I   R   C   P   T   I   F   V   D   C   L   K   D   T   C   I   D  
LdIV1_JGS      AAA GAT AGT ATT CGA TGC CCA ACC ATT TTC GTT GAT TGT TTG AAA GAT ACG TGT ATT GAT 
                K   D   S   I   R   C   P   T   I   F   V   D   C   L   K   D   T   C   I   D  
LdIV1_ZY       AAA GAT AGT ATT CGA TGC CCA ACC ATT TTC GTT GAT TGT TTG AAA GAT ACG TGT ATT GAT 
                K   D   S   I   R   C   P   T   I   F   V   D   C   L   K   D   T   C   I   D  
LdIV1_NJ       AAA GAT AGT ATT CGA TGC CCA ACT ATT TTT GTT GAT TGT TTG AAA GAT ACG TGT ATT GAT 
                K   D   S   I   R   C   P   T   I   F   V   D   C   L   K   D   T   C   I   D  
LdIV1_CT       AAA GAT AGT ATT CGA TGC CCA ACT ATT TTT GTT GAT TGT TTG AAA GAT ACG TGT ATT GAT 
                K   D   S   I   R   C   P   T   I   F   V   D   C   L   K   D   T   C   I   D  
KJ629170.1     AAA GAT AGT ATT CGA TGC CCA ACT ATT TTT GTT GAT TGT TTG AAA GAT ACG TGT ATT GAT 
                                            =       =                                          


[2661..2680]
                I   N   K   C   K   I   P   G   K   T   R   I   F   S   I   S   P   V   Q   Y  
MT753155.1     ATT AAT AAA TGT AAA ATT CCA GGA AAA ACA CGT ATA TTT TCT ATC TCT CCA GTG CAA TAT 
                I   N   K   C   K   I   P   G   K   T   R   I   F   S   I   S   P   V   Q   Y  
MN938851.1     ATT AAT AAA TGT AAA ATT CCA GGA AAA ACA CGT ATA TTT TCT ATC TCT CCA GTG CAA TAT 
                I   N   K   C   K   I   P   G   K   T   R   I   F   S   I   S   P   V   Q   Y  
LdIV1_JGS      ATT AAT AAA TGT AAA ATT CCA GGG AAA ACG CGT ATA TTT TCT ATC TCT CCA GTG CAA TAT 
                I   N   K   C   K   I   P   G   K   T   R   I   F   S   I   S   P   V   Q   Y  
LdIV1_ZY       ATT AAT AAA TGT AAA ATT CCA GGG AAA ACG CGT ATA TTT TCT ATC TCT CCA GTG CAA TAT 
                I   N   K   C   K   I   P   G   K   T   R   I   F   S   I   S   P   V   Q   Y  
LdIV1_NJ       ATT AAT AAA TGT AAA ATT CCA GGG AAA ACA CGT ATA TTT TCT ATC TCT CCA GTG CAA TAT 
                I   N   K   C   K   I   P   G   K   T   R   I   F   S   I   S   P   V   Q   Y  
LdIV1_CT       ATT AAT AAA TGT AAA ATT CCA GGG AAA ACA CGC ATA TTT TCT ATC TCT CCA GTG CAA TAT 
                I   N   K   C   K   I   P   G   K   T   R   I   F   S   I   S   P   V   Q   Y  
KJ629170.1     ATT AAT AAA TGT AAA ATT CCA GGG AAA ACA CGC ATA TTT TCT ATC TCT CCA GTG CAA TAT 
                                            =       =   =                                      


[2681..2700]
                T   I   A   F   K   Q   Y   F   G   D   F   L   A   S   Y   Q   E   A   R   L  
MT753155.1     ACA ATA GCC TTT AAG CAA TAT TTT GGT GAT TTT TTA GCG TCA TAT CAG GAA GCC CGT TTG 
                T   I   A   F   K   Q   Y   F   G   D   F   L   A   S   Y   Q   E   A   R   L  
MN938851.1     ACA ATA GCC TTT AAG CAA TAT TTT GGT GAT TTT TTA GCG TCA TAT CAG GAA GCC CGT TTG 
                T   I   A   F   K   Q   Y   F   G   D   F   L   A   S   Y   Q   E   A   R   L  
LdIV1_JGS      ACA ATA GCC TTC AAG CAA TAT TTT GGT GAT TTT TTA GCG TCA TAT CAG GAA GCC CGT CTG 
                T   I   A   F   K   Q   Y   F   G   D   F   L   A   S   Y   Q   E   A   R   L  
LdIV1_ZY       ACA ATA GCC TTC AAG CAA TAT TTT GGT GAT TTT TTA GCG TCA TAT CAG GAA GCC CGT CTG 
                T   I   A   F   K   Q   Y   F   G   D   F   L   A   S   Y   Q   E   A   R   L  
LdIV1_NJ       ACA ATA GCC TTC AAG CAA TAT TTT GGT GAT TTT TTA GCG TCA TAT CAG GAA GCC CGT CTG 
                T   I   A   F   K   Q   Y   F   G   D   F   L   A   S   Y   Q   E   A   R   L  
LdIV1_CT       ACA ATA GCC TTC AAG CAA TAT TTT GGT GAT TTT TTA GCG TCA TAT CAG GAA GCC CGT CTG 
                T   I   A   F   K   Q   Y   F   G   D   F   L   A   S   Y   Q   E   A   R   L  
KJ629170.1     ACA ATA GCC TTC AAG CAA TAT TTT GGT GAT TTT TTA GCG TCA TAT CAG GAA GCC CGT CTG 
                            =                                                               =  


[2701..2720]
                K   A   E   H   G   I   G   L   N   V   D   S   L   E   W   S   Q   V   A   N  
MT753155.1     AAA GCT GAA CAT GGT ATC GGA CTT AAT GTT GAT TCT TTG GAG TGG TCT CAA GTT GCA AAT 
                K   A   E   H   G   I   G   L   N   V   D   S   L   E   W   S   Q   V   A   N  
MN938851.1     AAA GCT GAA CAT GGT ATC GGA CTT AAT GTT GAT TCT TTG GAG TGG TCT CAA GTT GCA AAT 
                K   A   E   H   G   I   G   L   N   V   D   S   L   E   W   S   Q   V   A   N  
LdIV1_JGS      AAA GCT GAA CAT GGT ATC GGA CTT AAC GTC GAT TCT TTG GAG TGG TCT CAA GTT GCA AAT 
                K   A   E   H   G   I   G   L   N   V   D   S   L   E   W   S   Q   V   A   N  
LdIV1_ZY       AAA GCT GAA CAT GGT ATC GGA CTT AAC GTC GAT TCT TTG GAG TGG TCT CAA GTT GCA AAT 
                K   A   E   H   G   I   G   L   N   V   D   S   L   E   W   S   Q   V   A   N  
LdIV1_NJ       AAA GCT GAA CAT GGT ATC GGA CTT AAC GTC GAT TCT TTG GAG TGG TCT CAA GTT GCA AAT 
                K   A   E   H   G   I   G   L   N   V   D   S   L   E   W   S   Q   V   A   N  
LdIV1_CT       AAA GCT GAA CAT GGT ATT GGA CTT AAC GTC GAT TCT TTG GAG TGG TCT CAA GTT GCA AAT 
                K   A   E   H   G   I   G   L   N   V   D   S   L   E   W   S   Q   V   A   N  
KJ629170.1     AAA GCT GAA CAT GGT ATC GGA CTT AAC GTC GAT TCT TTG GAG TGG TCT CAA GTT GCA AAT 
                                    =           =   =                                          


[2721..2740]
                Y   I   T   T   Y   G   N   N   I   I   A   G   D   Y   K   N   F   G   P   S  
MT753155.1     TAC ATA ACT ACA TAC GGA AAT AAT ATT ATA GCT GGT GAT TAT AAA AAT TTT GGT CCA AGT 
                Y   I   T   T   Y   G   N   N   I   I   A   G   D   Y   K   N   F   G   P   S  
MN938851.1     TAC ATA ACT ACA TAC GGA AAT AAT ATT ATA GCT GGT GAT TAT AAA AAT TTT GGT CCA AGT 
                Y   I   T   T   Y   G   N   N   I   I   A   G   D   Y   K   N   F   G   P   S  
LdIV1_JGS      TAC ATA ACT ACA TAT GGA AAT AAT ATT ATA GCT GGT GAT TAT AAA AAT TTT GGC CCG AGT 
                Y   I   T   T   Y   G   N   N   I   I   A   G   D   Y   K   N   F   G   P   S  
LdIV1_ZY       TAC ATA ACT ACA TAT GGA AAT AAT ATT ATA GCT GGT GAT TAT AAA AAT TTT GGC CCG AGT 
                Y   I   T   T   Y   G   N   N   I   I   A   G   D   Y   K   N   F   G   P   S  
LdIV1_NJ       TAC ATA ACT ACA TAT GGA AAT AAT ATT ATA GCT GGT GAT TAT AAA AAT TTT GGC CCG AGT 
                Y   I   T   T   Y   G   N   N   I   I   A   G   D   Y   K   N   F   G   P   S  
LdIV1_CT       TAC ATA ACT ACA TAT GGA AAT AAT ATT ATA GCT GGT GAT TAT AAA AAT TTT GGC CCG AGT 
                Y   I   T   T   Y   G   N   N   I   I   A   G   D   Y   K   N   F   G   P   S  
KJ629170.1     TAC ATA ACT ACA TAC GGA AAT AAT ATT ATA GCT GGT GAT TAT AAA AAT TTT GGC CCA AGT 
                                =                                                   =   =      


[2741..2760]
                L   M   L   K   C   V   E   E   A   F   N   I   I   M   A   W   Y   E   R   Y  
MT753155.1     TTG ATG TTA AAA TGT GTA GAA GAG GCA TTC AAT ATA ATT ATG GCT TGG TAT GAA CGT TAT 
                L   M   L   K   C   V   E   E   A   F   N   I   I   M   A   W   Y   E   R   Y  
MN938851.1     TTG ATG TTA AAA TGT GTA GAA GAG GCA TTC AAT ATA ATT ATG GCT TGG TAT GAA CGT TAT 
                L   M   L   K   C   V   E   E   A   F   N   I   I   M   A   W   Y   E   R   Y  
LdIV1_JGS      TTG ATG TTA AAA TGT GTA GAA GAG GCA TTC AAT ATA ATT ATG GCT TGG TAT GAA CGT TAT 
                L   M   L   K   C   V   E   E   A   F   N   I   I   M   A   W   Y   E   R   Y  
LdIV1_ZY       TTG ATG TTA AAA TGT GTA GAA GAG GCA TTC AAT ATA ATT ATG GCT TGG TAT GAA CGT TAT 
                L   M   L   K   C   V   E   E   A   F   N   I   I   M   A   W   Y   E   R   Y  
LdIV1_NJ       TTG ATG TTA AAA TGT GTA GAA GAG GCA TTC AAT ATA ATT ATG GCT TGG TAT GAA CGT TAT 
                L   M   L   K   C   V   E   E   A   F   N   I   I   M   A   W   Y   E   R   Y  
LdIV1_CT       TTG ATG TTA AAA TGT GTA GAA GAG GCA TTC AAT ATA ATT ATG GCT TGG TAT GAA CGT TAT 
                L   M   L   K   C   V   E   E   A   F   N   I   I   M   A   W   Y   E   R   Y  
KJ629170.1     TTG ATG TTA AAA TGT GTA GAA GAG GCA TTC AAT ATA ATT ATG GCT TGG TAT GAA CGT TAT 
                                                                                               


[2761..2780]
                D   N   D   A   E   R   Q   R   V   R   R   V   L   L   S   E   I   V   H   A  
MT753155.1     GAC AAC GAT GCT GAA CGA CAG CGT GTG CGT AGA GTA TTG TTG TCA GAA ATT GTT CAT GCC 
                D   N   D   A   E   R   Q   R   V   R   R   V   L   L   S   E   I   V   H   A  
MN938851.1     GAC AAC GAT GCT GAA CGA CAG CGT GTG CGT AGA GTA TTG TTG TCA GAA ATT GTT CAT GCC 
                D   N   N   T   E   R   Q   R   V   R   R   V   L   L   S   E   I   V   H   A  
LdIV1_JGS      GAC AAT AAC ACT GAA CGA CAG CGT GTG CGT AGA GTA TTG TTG TCA GAA ATT GTG CAT GCC 
                D   N   N   T   E   R   Q   R   V   R   R   V   L   L   S   E   I   V   H   A  
LdIV1_ZY       GAC AAT AAC ACT GAA CGA CAG CGT GTG CGT AGA GTA TTG TTG TCA GAA ATT GTG CAT GCC 
                D   N   N   T   E   R   Q   R   V   R   R   V   L   L   S   E   I   V   H   A  
LdIV1_NJ       GAC AAT AAC ACT GAA CGA CAG CGT GTG CGT AGA GTG TTG TTG TCA GAA ATT GTG CAT GCC 
                D   N   N   T   E   R   Q   R   V   R   R   V   L   L   S   E   I   V   H   A  
LdIV1_CT       GAC AAT AAC ACT GAA CGA CAG CGT GTG CGT AGA GTA TTG TTG TCA GAA ATT GTG CAT GCC 
                D   N   N   T   E   R   Q   R   V   R   R   V   L   L   S   E   I   V   H   A  
KJ629170.1     GAC AAC AAC ACT GAA CGA CAG CGT GTG CGT AGA GTG TTG TTG TCA GAA ATT GTG CAT GCC 
                    =   ^   ^                               =                       =          


[2781..2800]
                K   H   L   C   L   N   V   V   Y   G   V   P   C   G   I   P   S   G   S   P  
MT753155.1     AAG CAT TTA TGT TTG AAT GTT GTG TAT GGT GTT CCA TGT GGT ATT CCT TCA GGA AGT CCT 
                K   H   L   C   L   N   V   V   Y   G   V   P   C   G   I   P   S   G   S   P  
MN938851.1     AAG CAT TTA TGT TTG AAT GTT GTG TAT GGT GTT CCA TGT GGT ATC CCT TCA GGA AGT CCT 
                K   H   L   C   L   N   V   V   Y   G   V   P   C   G   I   P   S   G   S   P  
LdIV1_JGS      AAG CAT TTA TGT TTG AAT GTT GTG TAT GGT GTT CCA TGT GGT ATC CCT TCA GGA AGT CCT 
                K   H   L   C   L   N   V   V   Y   G   V   P   C   G   I   P   S   G   S   P  
LdIV1_ZY       AAG CAT TTA TGT TTG AAT GTT GTG TAT GGT GTT CCA TGT GGT ATC CCT TCA GGA AGT CCT 
                K   H   L   C   L   N   V   V   Y   G   V   P   C   G   I   P   S   G   S   P  
LdIV1_NJ       AAG CAT TTA TGT TTG AAT GTT GTG TAT GGT GTT CCA TGT GGT ATC CCT TCA GGA AGT CCT 
                K   H   L   C   L   N   V   V   Y   G   V   P   C   G   I   P   S   G   S   P  
LdIV1_CT       AAG CAT TTA TGT TTG AAT GTT GTG TAT GGT GTT CCA TGT GGT ATC CCT TCA GGA AGT CCT 
                K   H   L   C   L   N   V   V   Y   G   V   P   C   G   I   P   S   G   S   P  
KJ629170.1     AAG CAT TTA TGT CTG AAT GTT GTG TAT GGT GTT CCA TGT GGT ATC CCT TCA GGA AGT CCT 
                                =                                       =                      


[2801..2820]
                I   T   T   P   L   N   S   L   V   N   S   L   Y   L   R   C   G   W   K   S  
MT753155.1     ATA ACC ACT CCG TTA AAT AGT TTA GTT AAT TCA TTA TAT CTC AGG TGT GGT TGG AAA AGT 
                I   T   T   P   L   N   S   L   V   N   S   L   Y   L   R   C   G   W   K   S  
MN938851.1     ATA ACC ACT CCG TTA AAT AGT TTA GTT AAT TCA TTA TAT CTC AGG TGT GGT TGG AAA AGT 
                I   T   T   P   L   N   S   L   V   N   S   L   Y   L   R   C   G   W   K   S  
LdIV1_JGS      ATA ACC ACT CCG TTA AAT AGT TTA GTT AAT TCA TTA TAT CTT AGG TGT GGT TGG AAA AGT 
                I   T   T   P   L   N   S   L   V   N   S   L   Y   L   R   C   G   W   K   S  
LdIV1_ZY       ATA ACC ACT CCG TTA AAT AGT TTA GTT AAT TCA TTA TAT CTT AGG TGT GGT TGG AAA AGT 
                I   T   T   P   L   N   S   L   V   N   S   L   Y   L   R   C   G   W   K   S  
LdIV1_NJ       ATA ACC ACT CCG TTA AAT AGT TTA GTT AAT TCA TTA TAT CTT AGG TGT GGT TGG AAA AGT 
                I   T   T   P   L   N   S   L   V   N   S   L   Y   L   R   C   G   W   K   S  
LdIV1_CT       ATA ACC ACT CCG TTA AAT AGT TTA GTT AAT TCA TTA TAT CTT AGG TGT GGT TGG AAA AGT 
                I   T   T   P   L   N   S   L   V   N   S   L   Y   L   R   C   G   W   K   S  
KJ629170.1     ATA ACC ACT CCG TTA AAT AGT TTA GTT AAT TCA TTA TAT CTT AGG TGT GGT TGG AAA AGT 
                                                                    =                          


[2821..2840]
                I   T   G   Q   N   F   S   I   M   H   D   N   I   K   I   L   T   Y   G   D  
MT753155.1     ATA ACA GGG CAA AAT TTT AGT ATA ATG CAT GAT AAT ATT AAG ATT TTA ACA TAT GGT GAT 
                I   T   G   Q   N   F   S   I   M   H   D   N   I   K   I   L   T   Y   G   D  
MN938851.1     ATA ACA GGG CAA AAT TTT AGT ATA ATG CAT GAT AAT ATT AAG ATT TTA ACA TAT GGT GAT 
                I   T   G   Q   N   F   S   V   M   H   D   N   I   K   I   L   T   Y   G   D  
LdIV1_JGS      ATA ACA GGG CAA AAC TTC AGT GTA ATG CAT GAT AAT ATT AAG ATT TTA ACA TAT GGT GAT 
                I   T   G   Q   N   F   S   V   M   H   D   N   I   K   I   L   T   Y   G   D  
LdIV1_ZY       ATA ACA GGG CAA AAC TTC AGT GTA ATG CAT GAT AAT ATT AAG ATT TTA ACA TAT GGT GAT 
                I   T   G   Q   N   F   S   V   M   H   D   N   I   K   I   L   T   Y   G   D  
LdIV1_NJ       ATA ACA GGG CAA AAC TTC AGT GTA ATG CAT GAT AAT ATT AAG ATT TTA ACA TAT GGT GAT 
                I   T   G   Q   N   F   S   V   M   H   D   N   I   K   I   L   T   Y   G   D  
LdIV1_CT       ATA ACA GGG CAA AAC TTC AGT GTA ATG CAT GAT AAT ATT AAG ATT TTA ACA TAT GGT GAT 
                I   T   G   Q   N   F   S   V   M   H   D   N   I   K   I   L   T   Y   G   D  
KJ629170.1     ATA ACA GGG CAA AAC TTC AGT GTA ATG CAT GAT AAT ATT AAG ATT TTA ACA TAT GGT GAT 
                                =   =       ^                                                  


[2841..2860]
                D   V   C   V   N   V   S   D   E   F   K   N   I   Y   N   T   E   S   L   S  
MT753155.1     GAT GTA TGT GTG AAT GTA AGT GAT GAA TTT AAG AAT ATA TAT AAC ACA GAA TCT TTG TCT 
                D   V   C   V   N   V   S   D   E   F   K   N   I   Y   N   T   E   S   L   S  
MN938851.1     GAT GTA TGT GTG AAT GTA AGT GAT GAA TTT AAG AAT ATA TAT AAC ACA GAA TCT TTG TCT 
                D   V   C   V   N   V   S   D   E   F   K   N   I   Y   N   T   E   S   L   S  
LdIV1_JGS      GAT GTA TGT GTG AAT GTA AGT GAT GAA TTT AAG AAT ATA TAT AAT ACA GAA TCT TTA TCT 
                D   V   C   V   N   V   S   D   E   F   K   N   I   Y   N   T   E   S   L   S  
LdIV1_ZY       GAT GTA TGT GTG AAT GTA AGT GAT GAA TTT AAG AAT ATA TAT AAT ACA GAA TCT TTA TCT 
                D   V   C   V   N   V   S   D   E   F   K   N   I   Y   N   T   E   S   L   S  
LdIV1_NJ       GAT GTA TGT GTG AAT GTA AGC GAT GAA TTT AAG AAT ATA TAT AAT ACA GAA TCT TTA TCT 
                D   V   C   V   N   V   S   D   E   F   K   N   I   Y   N   T   E   S   L   S  
LdIV1_CT       GAT GTA TGT GTG AAT GTA AGC GAT GAA TTT AAG AAT ATA TAT AAT ACA GAA TCT TTA TCT 
                D   V   C   V   N   V   S   D   E   F   K   N   I   Y   N   T   E   S   L   S  
KJ629170.1     GAT GTA TGT GTG AAT GTA AGT GAT GAA TTT AAG AAT ATA TAT AAC ACA GAA TCT TTG TCT 
                                        =                               =               =      


[2861..2880]
                L   F   F   K   N   Y   N   I   V   F   T   D   I   D   K   S   D   R   L   I  
MT753155.1     CTA TTT TTT AAA AAT TAT AAT ATT GTA TTT ACT GAT ATT GAT AAA AGT GAT AGG TTA ATT 
                L   F   F   K   N   Y   N   I   V   F   T   D   I   D   K   S   D   R   L   I  
MN938851.1     CTA TTT TTT AAA AAT TAT AAT ATT GTA TTT ACT GAT ATT GAT AAA AGT GAT AGG TTA ATT 
                L   F   F   K   K   Y   N   I   V   F   T   D   I   D   K   S   D   R   L   I  
LdIV1_JGS      TTA TTT TTT AAA AAA TAT AAT ATT GTA TTT ACT GAT ATT GAT AAA AGT GAT AGG TTA ATT 
                L   F   F   K   K   Y   N   I   V   F   T   D   I   D   K   S   D   R   L   I  
LdIV1_ZY       TTA TTT TTT AAA AAA TAT AAT ATT GTA TTT ACT GAT ATT GAT AAA AGT GAT AGG TTA ATT 
                L   F   F   K   K   Y   N   I   V   F   T   D   I   D   K   S   D   R   L   I  
LdIV1_NJ       TTA TTT TTT AAA AAA TAT AAT ATT GTA TTT ACT GAT ATT GAT AAA AGT GAT AGG TTA ATT 
                L   F   F   K   K   Y   N   I   V   F   T   D   I   D   K   S   D   R   L   I  
LdIV1_CT       TTA TTT TTT AAA AAA TAT AAT ATT GTA TTT ACT GAT ATT GAT AAA AGT GAT AGG TTA ATT 
                L   F   F   K   N   Y   D   I   V   F   T   D   I   D   K   S   D   R   L   I  
KJ629170.1     TTA TTT TTT AAA AAT TAT GAT ATT GTA TTT ACT GAT ATT GAT AAA AGC GAT AGG TTA ATT 
                =               ^       ^                                   =                  


[2881..2900]
                N   Y   R   S   L   E   T   C   S   F   L   K   R   N   F   R   L   H   P   N  
MT753155.1     AAT TAT AGA TCG TTA GAA ACG TGT TCA TTT TTA AAA AGA AAT TTT AGA TTA CAT CCC AAC 
                N   Y   R   S   L   E   T   C   S   F   L   K   R   N   F   R   L   H   P   N  
MN938851.1     AAT TAT AGA TCG TTA GAA ACG TGT TCA TTT TTA AAA AGA AAT TTT AGA TTA CAT CCC AAC 
                N   Y   R   S   L   E   T   C   S   F   L   K   R   N   F   R   L   H   P   N  
LdIV1_JGS      AAT TAT AGA TCA TTA GAA ACG TGT TCA TTT TTA AAA AGA AAT TTT AGA TTA CAT CCC AAC 
                N   Y   R   S   L   E   T   C   S   F   L   K   R   N   F   R   L   H   P   N  
LdIV1_ZY       AAT TAT AGA TCA TTA GAA ACG TGT TCA TTT TTA AAA AGA AAT TTT AGA TTA CAT CCC AAC 
                N   Y   R   S   L   E   T   C   S   F   L   K   R   N   F   R   L   H   P   N  
LdIV1_NJ       AAT TAT AGA TCA TTA GAA ACG TGT TCA TTT TTA AAA AGA AAT TTT AGA TTA CAT CCC AAC 
                N   Y   R   S   L   E   T   C   S   F   L   K   R   N   F   R   L   H   P   N  
LdIV1_CT       AAT TAT AGA TCA TTA GAA ACG TGT TCA TTT TTA AAA AGA AAT TTT AGA TTA CAT CCC AAC 
                N   Y   R   S   L   E   T   C   S   F   L   K   R   N   F   R   L   H   P   N  
KJ629170.1     AAT TAT AGA TCA TTA GAA ACG TGT TCA TTT TTA AAA AGA AAT TTT AGA TTA CAT CCC AAC 
                            =                                                                  


[2901..2920]
                S   N   A   I   F   L   A   P   I   E   L   Q   S   I   R   K   C   V   N   W  
MT753155.1     AGT AAT GCT ATT TTT CTA GCA CCT ATC GAA CTC CAG AGT ATT CGA AAA TGT GTT AAT TGG 
                S   N   A   I   F   L   A   P   I   E   L   Q   S   I   R   K   C   V   N   W  
MN938851.1     AGT AAT GCT ATT TTT CTA GCA CCT ATC GAA CTC CAG AGT ATT CGA AAA TGT GTT AAT TGG 
                S   N   A   I   F   L   A   P   I   E   L   Q   S   I   R   K   C   V   N   W  
LdIV1_JGS      AGT AAT GCT ATT TTT CTA GCA CCT ATC GAA CTC CAG AGT ATT CGA AAA TGT GTC AAT TGG 
                S   N   A   I   F   L   A   P   I   E   L   Q   S   I   R   K   C   V   N   W  
LdIV1_ZY       AGT AAT GCT ATT TTT CTA GCA CCT ATC GAA CTC CAG AGT ATT CGA AAA TGT GTC AAT TGG 
                S   N   A   I   F   L   A   P   I   E   L   Q   S   I   R   K   C   V   N   W  
LdIV1_NJ       AGT AAT GCT ATT TTT CTA GCA CCT ATC GAA CTC CAG AGT ATT CGA AAA TGT GTC AAT TGG 
                S   N   A   I   F   L   A   P   I   E   L   Q   S   I   R   K   C   V   N   W  
LdIV1_CT       AGT AAT GCT ATT TTT CTA GCA CCT ATC GAA CTC CAG AGT ATT CGA AAA TGT GTC AAT TGG 
                S   N   A   I   F   L   A   P   I   D   L   Q   S   I   R   K   C   V   N   W  
KJ629170.1     AGT AAT GCT ATT TTT CTA GCA CCT ATC GAC CTC CAG AGT ATT CGA AAA TGT GTC AAT TGG 
                                                    ^                               =          


[2921..2940]
                M   T   R   K   G   D   P   K   S   N   T   L   E   N   C   K   Q   A   C   E  
MT753155.1     ATG ACG CGA AAA GGA GAT CCA AAA AGT AAT ACT CTA GAG AAT TGT AAG CAA GCA TGT GAA 
                M   T   R   K   G   D   P   K   S   N   T   L   E   N   C   K   Q   A   C   E  
MN938851.1     ATG ACG CGA AAA GGA GAT CCA AAA AGT AAT ACT CTA GAG AAT TGT AAG CAA GCA TGT GAA 
                M   T   R   K   G   D   P   K   S   N   T   L   E   N   C   K   Q   A   C   E  
LdIV1_JGS      ATG ACG CGT AAA GGA GAT CCA AAA AGT AAT ACT CTA GAG AAT TGT AAG CAA GCA TGT GAA 
                M   T   R   K   G   D   P   K   S   N   T   L   E   N   C   K   Q   A   C   E  
LdIV1_ZY       ATG ACG CGT AAA GGA GAT CCA AAA AGT AAT ACT CTA GAG AAT TGT AAG CAA GCA TGT GAA 
                M   T   R   K   G   D   P   K   S   N   T   L   E   N   C   K   Q   A   C   E  
LdIV1_NJ       ATG ACG CGT AAA GGA GAT CCA AAA AGT AAT ACT CTA GAG AAT TGT AAG CAA GCA TGT GAA 
                M   T   R   K   G   D   P   K   S   N   T   L   E   N   C   K   Q   A   C   E  
LdIV1_CT       ATG ACG CGT AAA GGA GAT CCA AAA AGT AAT ACT CTA GAG AAT TGT AAG CAA GCA TGT GAA 
                M   T   R   K   G   D   P   K   S   N   T   L   E   N   C   K   Q   A   C   E  
KJ629170.1     ATG ACG CGT AAA GGA GAT CCA AAA AGT AAT ACT CTA GAG AAT TGT AAG CAA GCA TGC GAA 
                        =                                                               =      


[2941..2960]
                L   A   F   G   H   G   P   E   Y   Y   T   E   V   R   E   F   L   Q   R   A  
MT753155.1     CTT GCT TTT GGT CAC GGA CCA GAA TAC TAT ACC GAA GTA CGT GAG TTT CTC CAG CGA GCG 
                L   A   F   G   H   G   P   E   Y   Y   T   E   V   R   E   F   L   Q   R   A  
MN938851.1     CTT GCT TTT GGT CAC GGA CCA GAA TAC TAT ACC GAA GTA CGT GAG TTT CTC CAG CGA GCG 
                L   A   F   G   H   G   P   E   Y   Y   T   E   V   R   E   F   L   Q   R   A  
LdIV1_JGS      CTT GCT TTT GGT CAC GGA CCA GAA TAC TAT ACC GAA GTA CGT GAG TTT CTC CAG CGA GCG 
                L   A   F   G   H   G   P   E   Y   Y   T   E   V   R   E   F   L   Q   R   A  
LdIV1_ZY       CTT GCT TTT GGT CAC GGA CCA GAA TAC TAT ACC GAA GTA CGT GAG TTT CTC CAG CGA GCG 
                L   A   F   G   H   G   P   E   Y   Y   T   E   V   R   E   F   L   Q   R   A  
LdIV1_NJ       CTT GCT TTT GGT CAC GGA CCA GAA TAC TAT ACC GAA GTA CGT GAG TTT CTC CAG CGA GCG 
                L   A   F   G   H   G   P   E   Y   Y   T   E   V   R   E   F   L   Q   R   A  
LdIV1_CT       CTT GCT TTT GGT CAC GGA CCA GAA TAC TAT ACC GAA GTA CGT GAG TTT CTC CAG CGA GCG 
                L   A   F   G   H   G   P   E   Y   Y   T   E   V   R   E   F   L   Q   R   T  
KJ629170.1     CTT GCT TTT GGT CAC GGA CCA GAA TAC TAT ACC GAA GTA CGT GAG TTT CTC CAG CGA ACG 
                                                                                            ^  


[2961..2980]
                C   M   R   Q   L   G   C   S   F   T   A   P   R   W   Y   E   K   S   E   I  
MT753155.1     TGT ATG CGT CAA TTG GGA TGC AGT TTT ACA GCT CCA CGA TGG TAT GAG AAA TCC GAA ATT 
                C   M   R   Q   L   G   C   S   F   T   A   P   R   W   Y   E   K   S   E   I  
MN938851.1     TGT ATG CGT CAA TTG GGA TGC AGT TTT ACA GCT CCA CGA TGG TAT GAG AAA TCC GAA ATT 
                C   M   R   Q   L   G   C   S   F   T   A   P   R   W   Y   E   K   S   E   I  
LdIV1_JGS      TGT ATG CGT CAA TTG GGA TGC AGT TTT ACA GCT CCA CGA TGG TAT GAG AAA TCC GAA ATT 
                C   M   R   Q   L   G   C   S   F   T   A   P   R   W   Y   E   K   S   E   I  
LdIV1_ZY       TGT ATG CGT CAA TTG GGA TGC AGT TTT ACA GCT CCA CGA TGG TAT GAG AAA TCC GAA ATT 
                C   M   R   R   L   G   C   S   F   T   A   P   R   W   Y   E   K   S   E   I  
LdIV1_NJ       TGT ATG CGT CGA TTG GGA TGC AGT TTT ACA GCT CCA CGA TGG TAT GAG AAA TCC GAA ATT 
                C   M   R   Q   L   G   C   S   F   T   A   P   R   W   Y   E   K   S   E   I  
LdIV1_CT       TGT ATG CGT CAA TTG GGA TGC AGT TTT ACA GCT CCA CGA TGG TAT GAG AAA TCC GAA ATT 
                C   M   R   Q   L   G   C   S   F   T   A   P   R   W   Y   E   K   S   E   I  
KJ629170.1     TGT ATG CGT CAA TTG GGA TGC AGT TTT ACA GCT CCA CGA TGG TAT GAG AAA TCC GAA ATT 
                            ^                                                                  


[2981..2984]
                C   Y   G   I  
MT753155.1     TGT TAT GGT ATA 
                C   Y   G   I  
MN938851.1     TGT TAT GGT ATA 
                C   Y   G   I  
LdIV1_JGS      TGT TAT GGT ATA 
                C   Y   G   I  
LdIV1_ZY       TGT TAT GGT ATA 
                C   Y   G   I  
LdIV1_NJ       TGT TAT GGT ATA 
                C   Y   G   I  
LdIV1_CT       TGT TAT GGT ATA 
                C   Y   G   I  
KJ629170.1     TGT TAT GGT ATA
```
