# Supplementary figures and images for: Lymantria Dispar Iflavirus 1 RNA Comprises a Large Proportion of RNA in Adult L. dispar Moths
Source: Insects. 2023 May 15;14(5):466. doi: 10.3390/insects14050466 (PMC10231084; doi:10.3390/insects14050466)

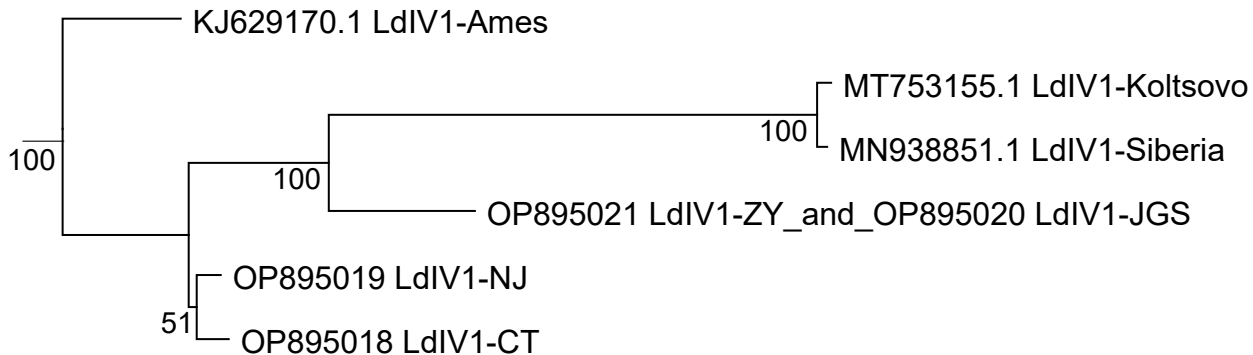

Figure S2. Subtree of Figure 2 polyprotein ORF tree, showing relationships of LdIV1 isolates

Supplement: Supplementary file 1 [file insects-14-00466-s001.zip › Figure S2.pdf]
